# Gene-environment interactions in the influence of maternal education on adolescent neurodevelopment using ABCD study

**DOI:** 10.1126/sciadv.adp3751

**Published:** 2024-11-15

**Authors:** Runye Shi, Xiao Chang, Tobias Banaschewski, Gareth J. Barker, Arun L. W. Bokde, Sylvane Desrivières, Herta Flor, Antoine Grigis, Hugh Garavan, Penny Gowland, Andreas Heinz, Rüdiger Brühl, Jean-Luc Martinot, Marie-Laure Paillère Martinot17,, Eric Artiges, Frauke Nees, Dimitri Papadopoulos Orfanos, Luise Poustka, Sarah Hohmann, Nathalie Holz, Michael N. Smolka, Nilakshi Vaidya, Henrik Walter, Robert Whelan, Gunter Schumann, Xiaolei Lin, Jianfeng Feng, IMAGEN Consortium

**Affiliations:** ^1^ School of Data Science, Fudan University, Shanghai, China.; ^2^ Institute of Science and Technology for Brain-Inspired Intelligence, Fudan University, Shanghai, China.; ^3^ Key Laboratory of Computational Neuroscience and Brain-Inspired Intelligence (Fudan University), Ministry of Education, Shanghai, China.; ^4^ MOE Frontiers Center for Brain Science, Fudan University, Shanghai, China.; ^5^ Zhangjiang Fudan International Innovation Center, Shanghai, China.; ^6^ Department of Child and Adolescent Psychiatry and Psychotherapy, Central Institute of Mental Health, Medical Faculty Mannheim, Heidelberg University, Square J5, 68159 Mannheim, Germany.; ^7^ Department of Neuroimaging, Institute of Psychiatry, Psychology & Neuroscience, King’s College London, London, UK.; ^8^ Discipline of Psychiatry, School of Medicine and Trinity College Institute of Neuroscience, Trinity College Dublin, Dublin, Ireland.; ^9^ Social, Genetic and Developmental Psychiatry Centre, Institute of Psychiatry, Psychology & Neuroscience, King’s College London, London, UK.; ^10^ Institute of Cognitive and Clinical Neuroscience, Central Institute of Mental Health, Medical Faculty Mannheim, Heidelberg University, Square J5, Mannheim, Germany.; ^11^ Department of Psychology, School of Social Sciences, University of Mannheim, 68131 Mannheim, Germany.; ^12^ NeuroSpin, CEA, Université Paris-Saclay, F-91191 Gif-sur-Yvette, France.; ^13^ Departments of Psychiatry and Psychology, University of Vermont, 05405 Burlington, VT, USA.; ^14^ Sir Peter Mansfield Imaging Centre School of Physics and Astronomy, University of Nottingham, University Park, Nottingham, UK.; ^15^ Department of Psychiatry and Psychotherapy CCM, Charité–Universitätsmedizin, Berlin, corporate member of Freie Universität Berlin, Humboldt-Universität zu Berlin, and Berlin Institute of Health, Berlin, Germany.; ^16^ Physikalisch-Technische Bundesanstalt (PTB), Braunschweig and Berlin, Germany.; ^17^ Institut National de la Santé et de la Recherche Médicale, INSERM U A10 “Trajectoires développementales en psychiatrie”, Université Paris-Saclay, Ecole Normale supérieure Paris-Saclay, CNRS, Centre Borelli, Gif-sur-Yvette, France.; ^18^ AP-HP, Sorbonne Université, Department of Child and Adolescent Psychiatry, Pitié-Salpêtrière Hospital, Paris, France.; ^19^ Psychiatry Department, EPS Barthélémy Durand, Etampes, France.; ^20^ Institute of Medical Psychology and Medical Sociology, University Medical Center Schleswig-Holstein Kiel University, Kiel, Germany.; ^21^ Department of Child and Adolescent Psychiatry and Psychotherapy, University Medical Centre Göttingen, von-Siebold-Str. 5, 37075 Göttingen, Germany.; ^22^ Department of Child and Adolescent Psychiatry and Psychotherapy, University Medical Center Hamburg-Eppendorf, Hamburg, Germany.; ^23^ Department of Psychiatry and Psychotherapy, Technische Universität Dresden, Dresden, Germany.; ^24^ Centre for Population Neuroscience and Stratified Medicine (PONS), Department of Psychiatry and Psychotherapy, Charité Universitätsmedizin Berlin, Germany.; ^25^ School of Psychology and Global Brain Health Institute, Trinity College Dublin, Dublin, Ireland.; ^26^ Centre for Population Neuroscience and Precision Medicine (PONS), Institute for Science and Technology of Brain-inspired Intelligence (ISTBI), Fudan University, Shanghai, China.; ^27^ Department of Computer Science, University of Warwick, Coventry, UK.

## Abstract

Maternal education was strongly correlated with adolescent brain morphology, cognitive performances, and mental health. However, the molecular basis for the effects of maternal education on the structural neurodevelopment remains unknown. Here, we conducted gene-environment–wide interaction study using the Adolescent Brain Cognitive Development cohort. Seven genomic loci with significant gene-environment interactions (G×E) on regional gray matter volumes were identified, with enriched biological functions related to metabolic process, inflammatory process, and synaptic plasticity. Additionally, genetic overlapping results with behavioral and disease-related phenotypes indicated shared biological mechanism between maternal education modified neurodevelopment and related behavioral traits. Finally, by decomposing the multidimensional components of maternal education, we found that socioeconomic status, rather than family environment, played a more important role in modifying the genetic effects on neurodevelopment. In summary, our study provided analytical evidence for G×E effects regarding adolescent neurodevelopment and explored potential biological mechanisms as well as social mechanisms through which maternal education could modify the genetic effects on regional brain development.

## Teaser

The interplay between gene and maternal education on adolescent brain volumes was studied in both biological and social contexts.

## INTRODUCTION

Substantial brain development occurs during childhood, which continues up to early adolescence. While previous studies revealed high heritability regarding structural adolescent brain (*1*), the role of variable environmental factors, especially family socioeconomic status (SES), during adolescent neurodevelopment remains one of the most important questions in neuroscience. Neuroimaging studies have demonstrated that maternal education (ME), a central aspect of SES, is positively correlated with cortical surface areas in regions (middle and inferior temporal gyrus, superior frontal, inferior parietal, and postcentral) related to language, reading, various executive functions, and spatial skills (*2*), gray matter volume (GMV) of amygdala and hippocampal (*3*), and variability of regional growth patterns (*4*). Studies examining composite measures of SES and its different components suggested ME as one of the strongest predictors of children’s cognitive development (*5*–*7*), mental/physical health (*8*–*11*), and educational outcomes (*7*, *12*).

From the social perspective, ME is strongly associated with human capital and household wealth (*13*, *14*), stable family structure (*15*), and supportive parenting behaviors (*16*), including cognitive stimulation, selection of academically advantageous childcare arrangements, and high-quality child-directed speech (*17*, *18*). However, from a biological perspective, it remains unknown how ME could interplay with genetic components in structuring adolescent neurodevelopment.

Gene-environment–wide interaction study (GEWIS) is a widely used approach for identifying genetic loci with differential effects on the phenotype stratified by the levels of environmental exposure. It has been previously used to characterize the molecular basis of posttraumatic stress on the risk of suicidal behaviors, and identified extracellular matrix biology and synaptic plasticity as biological interactors on the genetic risk of suicidality (*19*). Here, by leveraging the genetic and neuroimaging data from a prospective multicenter adolescent cohort, we aim to investigate the interaction effects between ME and single-nucleotide polymorphisms (SNPs) on adolescent neurodevelopment by conducting GEWIS using additive genetic model. Multiple variants were identified to achieve significant main and interaction effects on neurodevelopment, with enriched biological functions relating to metabolic process, inflammatory process, and synaptic plasticity. Results were validated using an independent adolescent cohort and via gene-based and gene-set analyses. Additionally, genetic correlation analysis was conducted between genetic variants with significant gene environment interactions and those associated with multiple behavioral and disease-related phenotypes to examine potentially shared biological mechanism. Finally, conditional analyses indicated that, compared to family environment, ME is more likely to modify the genetic effects on neurodevelopment through socioeconomic status.

## RESULTS

### ME exhibited strong correlation with regional GMV

**Fig. 1.**
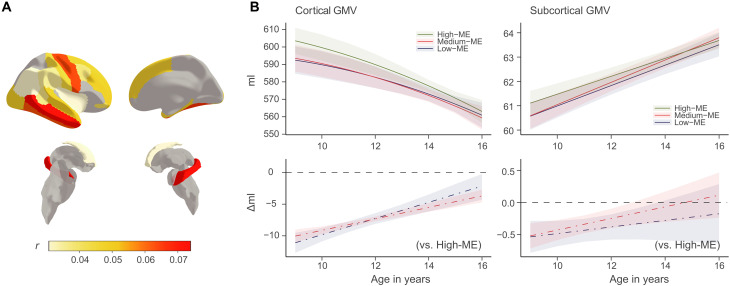
The influence of ME on regional GMVs. (**A**) Spearman correlations between ME and regional GMVs, adjusting for age, site, handedness, sex, and estimated intracranial volume. Regions in gray indicate nonsignificant correlations after BH-FDR correction. (**B**) Longitudinal effects of ME on cortical (left) and subcortical brain GMVs. Both GMVs (top) and GMV change rate (bottom) for participants with high, medium, and low levels of ME are estimated. The bands indicate 95% confidence intervals for predicted GMV at the top and relative regression coefficients at the bottom.

To facilitate comparisons across different educational systems, adolescents from the Adolescent Brain Cognitive Development (ABCD) Study and IMAGEN study were grouped into three categories based on their ME level: mother completed a university education level, the equivalent, or above (High-ME); mother completed a high school education and the equivalent (Medium-ME); and mother completed less than a high school education (Low-ME). Baseline characteristics of these 13,862 adolescents stratified by ME levels are shown in Table 1. Neither study showed significant sex differences in the distributions of ME (*X*^2^ = 4.08, *P* = 0.130 for ABCD; *X*^2^ = 0.35, *P* = 0.840 for IMAGEN). Higher ME was significantly associated with higher total GMV, adjusting for age, site, handedness, sex, and estimated intracranial volume (*r* = 0.09, *P* < 0.001). Brain regions exhibiting the strongest correlation with ME include middle temporal (*r* = 0.07, *P*_adj_ < 0.001), fusiform (*r* = 0.07, *P*_adj_ < 0.001), precentral (*r* = 0.06, *P*_adj_ < 0.001), inferior temporal (*r* = 0.06, *P*_adj_ < 0.001), and frontal pole (*r* = 0.05, *P*_adj_ < 0.001) [Fig. 1A and table S1; Benjamini-Yekutieli false discovery rate (BY-FDR) method]. We also examined the longitudinal associations between ME and structural neurodevelopment, and found that compared to High-ME, the effects of Medium-ME and Low-ME on total GMV remained significant until mid-adolescence, although weakening over time (Fig. 1B and table S2).

**Table 1.** Baseline characteristics for adolescents in the ABCD and IMAGEN cohorts, stratified by ME. Continuous characteristics were described using mean and SD, and categorical characteristics are presented as frequency and percentages per category. *P* values were obtained by one-way analysis of variance (ANOVA) test for continuous variables and chi-square test for categorical variables. ns: *P* ≥ 0.05, *P < 0.05, **P < 0.01, ***P < 0.001. High-ME, high maternal education, defined as mother completed a university education level, the equivalent, or above; Medium-ME, medium maternal education, defined as mother completed a high school education and the equivalent; Low-ME, low maternal education, defined as mother completed less than a high school education.

ABCD study					
	All participants (*N* = 11,780)	High-ME (*N* = 9567)	Medium-ME (*N* = 1234)	Low-ME (*N* = 763)	*P* value
Age (years), mean (SD)	9.93 ± 0.64	9.93 ± 0.65	9.90 ± 0.64	9.89 ± 0.63	ns
Male, *N* (%)	6147 (52.2%)	5000 (52.3%)	646 (52.4%)	370 (48.5%)	ns
Ethnic, *N* (%)					***
White	7459 (63.3%)	6571 (68.7%)	517 (41.9%)	276 (36.2%)	
Black	1840 (15.6%)	1156 (12.1%)	431 (34.9%)	199 (26.1%)	
Other	2480 (21.1%)	1840 (19.2%)	286 (23.2%)	288 (37.7%)	
Paternal education, *N* (%)					***
University, the equivalent, or above	7313 (77.1%)	6841 (85.6%)	275 (33.6%)	83 (16.5%)	
High school and the equivalent	1418 (14.9%)	879 (11.0%)	383 (46.8%)	122 (24.2%)	
Less than high school	754 (7.9%)	271 (3.4%)	160 (19.6%)	299 (59.3%)	
Socioeconomic condition, *N* (%)					***
≥$100K	4529 (42.0%)	4414 (49.3%)	73 (7.0%)	7 (1.2%)	
$50K–$100K	3049 (28.3%)	2682 (30.0%)	257 (24.6%)	65 (11.0%)	
<$50K	3193 (29.6%)	1858 (20.8%)	714 (68.4%)	519 (87.8%)	
Intracranial volume (liters), mean (SD)	1.49 ± 0.14	1.50 ± 0.14	1.46 ± 0.14	1.43 ± 0.14	***
Total brain volume (liters), mean (SD)	0.66 ± 0.06	0.66 ± 0.06	0.64 ± 0.06	0.63 ± 0.06	***
IMAGEN study					
	**All participants (*N* = 2082)**	**High-ME (*N* = 1056)**	**Medium-ME (*N* = 692)**	**Low-ME (*N* = 294)**	***P* value**
Age (years), mean (SD)	14.39 ± 0.40	14.39 ± 0.40	14.40 ± 0.41	14.40 ± 0.40	ns
Male, *N* (%)	1021 (49.0%)	522 (49.4%)	335 (48.4%)	148 (50.3%)	ns
Ethnic, *N* (%)					ns
White	1859 (89.3%)	943 (89.3%)	626 (90.5%)	260 (88.4%)	
Black	24 (1.2%)	14 (1.3%)	4 (0.6%)	4 (1.4%)	
Other	199 (9.6%)	99 (9.4%)	62 (9.0%)	30 (10.2%)	
Paternal education, *N* (%)					***
University, the equivalent, or above	1062 (52.3%)	808 (77.2%)	197 (28.9%)	51 (18.0%)	
High school and the equivalent	557 (27.4%)	155 (14.8%)	339 (49.8%)	57 (20.1%)	
Less than high school	411 (20.2%)	83 (7.9%)	145 (21.3%)	175 (61.8%)	
Socioeconomic condition	0.72 ± 1.03	0.58 ± 0.95	0.81 ± 1.07	0.96 ± 1.12	***
Intracranial volume (liters), mean (SD)	1.54 ± 0.15	1.55 ± 0.15	1.53 ± 0.14	1.51 ± 0.15	***
Total brain volume (liters), mean (SD)	0.60 ± 0.06	0.61 ± 0.06	0.60 ± 0.06	0.59 ± 0.06	***

### ME modified the genetic effects on neurodevelopment via metabolic process, inflammatory process, and synaptic plasticity

**Fig. 2.**
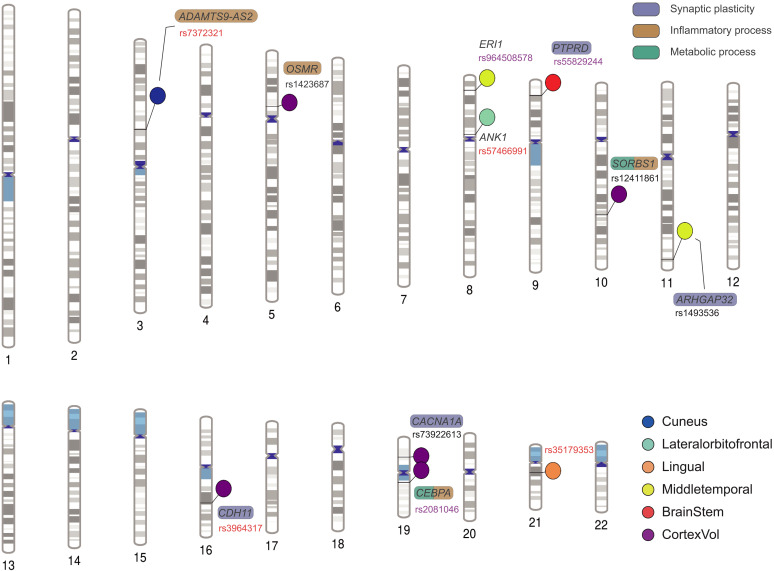
Genomic loci with genome-wide significant main and interaction effects with ME on regional brain GMVs. SNPs in red indicate interaction effects only, while those in purple indicate both main and interaction effects. These SNPs are then mapped to functional genes using position, molecular phenotype quantitative trait loci, chromatin interaction, and in silico functional prediction mapping methods. Genes involved in synaptic plasticity, inflammatory process, and metabolic process are highlighted in purple, brown, and green, respectively. Plot created using PhenoGram (http://visualization.ritchielab.org).

To investigate the role of ME on the genetic effects on structural neurodevelopment, we conducted GEWIS using 7662 adolescents in the ABCD study. To reduce the false-positive rate, Bonferroni-corrected genome-wide significance levels considering the number of independent traits were applied (Materials and Methods). Eleven independent loci were identified with genome-wide significant main or interaction effects (fig. S1 and Fig. 2). Among the seven loci with significant interaction effects where SNP effects vary by levels of ME, three of them also showed significant main effects on regional brain GMV. Absolute effect sizes of these loci on regional GMVs were relatively larger and more significant in adolescents with Low-ME, and considerably weaker among those with Medium-ME and High-ME (Table 2). These results, especially those found in loci with significant main effects and effects in adolescents with Low-ME, suggested a possible role of the genotype as a diathesis and low ME as a stressor, while the results found in loci with nonsignificant main effects were more likely to indicate the differential susceptibility to ME levels. Detailed annotations for all 11 significant loci are listed in table S3.

**Table 2.** Genomic loci showing genome-wide significant main or interaction effects with ME on brain GMVs. SNPs with significant main or interaction effects were clumped using PLINK, and only those with the smallest *P* values in each locus are shown in the table. The position of SNP was built upon GRCh38. BETA indicates the effect of each SNP on regional GMV among participants with high, medium, and low levels of ME. As ME was treated as a continuous variable, the genetic effects were estimated using a horizontal shift of ME. MAF, minor allele frequency.

Phenotype	SNP (Chr:BP)	Allele	MAF	High-ME		Medium-ME		Low-ME		G×ME effect	
				BETA	*P*	BETA	*P*	BETA	*P*	BETA	*P*
Cuneus	rs7372321 (3:64728139)	C/T	0.09	0.10	0.001	−0.28	1.07 × 10−5	−0.65	1.84 × 10^−7^	0.38	9.22 × 10^−9^
Lateral orbitofrontal	rs57466991 (8:41762594)	A/G	0.17	0.03	0.112	−0.20	2.29 × 10^−7^	−0.42	2.05 × 10^−8^	0.22	1.02 × 10^−8^
Lingual	rs35179353 (21:19854073)	C/T	0.11	0.06	0.016	−0.27	1.13 × 10^−6^	−0.59	5.27 × 10^−8^	0.32	9.14 × 10^−9^
Middle temporal	rs964508578 (8:9081668)	C/T	0.53	0.03	0.018	−0.13	3.81 × 10^−7^	−0.29	2.13 × 10^−9^	0.16	1.68 × 10^−10^
	rs1493536 (11:129307277)	C/T	0.44	0.00	0.897	0.14	8.71 × 10^−9^	0.29	5.42 × 10^−9^	−0.14	3.87 × 10^−8^
Brain stem	rs55829244 (9:9533520)	T/G	0.12	0.02	0.310	−0.22	4.91 × 10^−8^	−0.46	1.01 × 10^−8^	0.24	1.10 × 10^−8^
Cortex	rs1423687 (5:39103314)	A/G	0.35	1.2 × 10^−3^	0.913	0.12	1.11 × 10^−8^	0.23	7.18 × 10^−9^	−0.12	5.21 × 10^−8^
	rs12411861 (10:95539609)	G/A	0.18	−3.0 × 10^−3^	0.824	0.12	2.36 × 10^−8^	0.25	7.56 × 10^−9^	−0.13	4.62 × 10^−8^
	rs3964317 (16:65497034)	A/G	0.38	−0.02	0.164	0.10	2.48 × 10^−7^	0.22	1.50 × 10^−8^	−0.12	1.03 × 10^−8^
	rs73922613 (19:13549883)	A/G	0.05	0.01	0.617	−0.28	1.68 × 10^−8^	−0.57	5.54 × 10^−9^	0.29	1.26 × 10^−8^
	rs2081046 (19:33262430)	T/C	0.28	−0.04	0.003	0.10	6.27 × 10^−7^	0.23	9.34 × 10^−10^	−0.13	2.87 × 10^−11^

One intergenic SNP, rs2081046 on chromosome 19, was found to be functionally associated with CCAAT enhancer binding protein α (*CEBPA*), a transcription factor involved in glucose and lipid metabolism (*20*). This gene was also found to have immunomodulatory effects, such as regulating proinflammatory cytokines (*21*), and its deficiency or overexpression could abrogate granulocyte differentiation (*22*) or induce monocytic differentiation in mixed lineage leukemia (MLL) fusion protein-mediated leukemias (*23*). Three other SNPs were mapped to genes involved in glucose/lipid metabolic and inflammatory processes as well. Specifically, rs12411861 was an intron variant of *SORBS1*, which encodes a Casitas B-lineage lymphoma (CBL)–associated protein involved in the insulin signaling, insulin-stimulated glucose transport (*24*, *25*), and lipid biosynthetic process (*26*), while a few studies have also suggested a potential regulatory role of *SORBS1* in immune system through the nuclear factor κB (NF-κB) pathway (*27*). For another two SNPs, rs1423687 was functionally mapped to *OSMR* involved in cytokine signaling, especially the interleukin-6 (IL-6) family (*28*, *29*), and rs7372321 was an intro variant of *ADAMTS9-AS2*, which was widely studied in human cancers due to its relationship with the phosphatidylinositol 3-kinase (PI3K)/AKT signaling pathway (*30*, *31*) implicated in inflammation (*32*). In addition, the remaining SNPs showed evidence in synaptic activity of the brain, including an intron variant of *CACNA1A* (rs73922613), which encoded a subunit of neuronal calcium channel (*33*) and was involved in a broad phenotypic spectrum of early developmental delay (*34*) and neuropsychiatric disorders (*35*), an intron variant of *PTPRD* (rs55829244) encoding a neuronal cell adhesion molecule and synaptic specifier, and a variant mapped to *CDH11* (rs3964317) that correlated with altered dendritic complexity and neuronal/synaptic activity (*36*).

Note that, although loci identified with significant SNP×ME effects exhibit more significant effects on brain development in population with Low-ME, this is not the case using a whole-genome evaluation (fig. S2). On the basis of external genome-wide association study (GWAS) results for regional brain GMV (*37*, *38*), we found an increase of heritability among participants with High-ME. Moreover, following previous studies to control for potential confounding (*39*), we included both the covariate-by-environment and the covariate-by-gene interaction term in the GEWIS model as a sensitive analysis. Sixteen of 17 SNPs identified with significant interaction effects exhibited Benjamini–Hochberg false discovery rate (BH-FDR)–corrected significance (*P*_adj_ < 0.05) (table S4), although none of them passed the stringent genome-wide significance due to small sample size and increased model complexity. The inclusion of multiple interaction effects also complicates the interpretation of the G×E results, making it challenging to compare with the original GEWIS findings.

### GEWIS findings were successfully validated

**Fig. 3.**
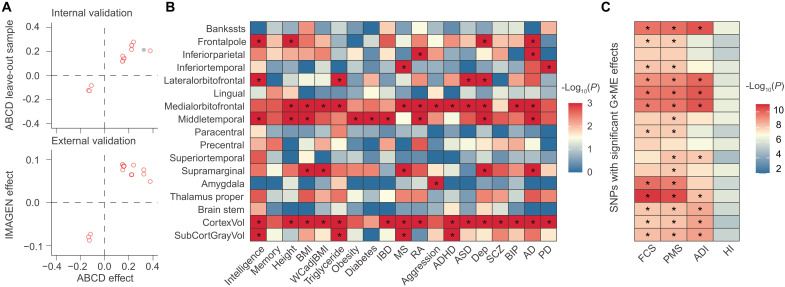
Validation of G×ME and the interpretation of the influence of ME on neurodevelopment. (**A**) Validation of significant GEWIS results obtained from ABCD using the leave-out samples as an internal validation (top) and independent adolescent study IMAGEN as an external validation (bottom). (**B**) Genetic overlap between SNPs showing G×ME effects on regional brain GMVs and those showing strong associations with multiple diseases and related traits. * indicates a significant overlap after BY-FDR correction. BMI, body mass index; WCadjBMI, waist circumference–adjusted BMI; IBD, inflammatory bowel disease; MS, multiple sclerosis; RA, rheumatoid arthritis; ADHD, attention-deficit/hyperactivity disorder; ASD, autism; Dep, depression; SCZ, schizophrenia; BIP, bipolar disorder; AD, Alzheimer’s disease; PD, Parkinson’s disease. (**C**) Significant G×ME effects after adjusting for family or social environmental factors. * indicates remained genome-wide significant G×ME effects. FCS, family conflict score; PMS, parenting monitoring score; ADI, area deprivation index; HI, household income.

Both internal and external validation were performed (Fig. 3A). First, the leave-out ABCD samples with their siblings were used as an internal validation set, where 15 of 17 SNPs (88%) identified with significant interaction effects were also found to have BH-FDR–adjusted significant interaction effects (fig. S3), although not reaching the genome-wide significance. Next, an independent external sample, IMAGEN cohort (*n* = 1982), was adopted to replicate the GEWIS findings. Because of the small sample size of IMAGEN and possible population heterogeneity due to age differences, study sites, etc., we examined the sign concordance of the SNP×ME effects between the two studies and reduction of *P* values after meta-analysis with ABCD (*40*). All significant SNP×ME effects identified in ABCD had the same effect direction with IMAGEN and smaller *P* values after meta-analysis with IMAGEN (table S5).

### Gene-based and gene-set analyses indicated similar mechanisms underlying the G×ME interactions on neurodevelopment

To reveal the mechanisms of ME on neurodevelopment at a higher biological level, we performed gene-based analysis using summary statistics from our GEWIS in MAGMA. Eleven genes were identified to achieve Bonferroni-corrected genome-wide significant interactions with ME (G×ME) on regional GMVs (table S6). Notably, we observed significant G×ME interactions for *CSMD1* on the whole cortical (*P* = 3.84 × 10^−12^), subcortical (*P* = 2.36 × 10^−7^), and middle temporal (*P* = 5.39 × 10^−7^) GMV, with suggestive effects on lateral orbitofrontal (*P* = 1.10 × 10^−5^), medial orbitofrontal (*P* = 3.48 × 10^−6^), and thalamus (*P* = 2.40 × 10^−6^) GMV. *CSMD1* encodes a complement pathway inhibitor in regulating C3/CR3-dependent axonal pruning (*41*) involved in the development of the central nervous system. Abnormal axonal pruning may induce both neurodegenerative and psychiatric disorders (*42*–*44*), and *CSMD1* has been reported to be correlated with cognitive functions including working memory and episodic memory (*45*, *46*). Other significant genes included *CTNNA2*, which encodes a cell adhesion protein related to dendritic spine and synaptic connection stability (*47*–*49*), and *ANK2*, which encodes a major ankyrin-B polypeptides required for normal structural connectivity in the central nervous system (CNS) (*50*). In addition, *SORBS1* (*P* = 5.32 × 10^−6^), with suggestive gene-based interaction effects, was also implicated in the SNP-based GEWIS.

To examine whether the identified genes with significant G×ME interaction effects converged on functional gene sets and pathways, we conducted gene-set analysis using MAGMA. Eight significant gene sets (table S7) were identified to be involved in neurodevelopment, including four sets related to inflammation regulation and synaptogenesis: GOBP_regulation_of_mast_cell_activation_involved_in_immune_response, GOBP_tachykinin_receptor_signaling_pathway, GOBP_chemorepulsion_of_axon, and Curated_gene_sets_gery_cebp_targets. These gene sets were found to have differential effects on accumbens (*P* = 1.21 × 10^−6^), lateral occipital (*P* = 9.47 × 10^−7^), caudal middle frontal (*P* = 1.03 × 10^−6^), and posterior cingulate (*P* = 3.15 × 10^−7^) GMVs separately, stratified by the levels of ME.

### Significant genetic overlaps were observed between G×ME effects on regional neurodevelopment and related traits

Independent SNP effect concordance analyses (iSECAs) were conducted to investigate the overlap between our GEWIS summary statistics and related traits, including neuropsychiatric and neurological disorders, and physical, psychological and disease-related phenotypes. Significant genomic overlaps (*P* < 1.0 × 10^−3^) were observed between variants with significant SNP×ME effects on medial orbitofrontal/middle temporal and related traits (Fig. 3B). These variants were also reported to be related to height, body mass index (BMI), rheumatoid arthritis (RA), depression, and Alzheimer’s disease (AD). These results indicated that the biological mechanisms through which ME could modify the genetic effects on specific brain regions are likely to overlap with behavioral and disease-related phenotypes. While significant pleiotropy was observed between G×ME effects on regional brain GMVs and GWAS of related traits, we did not find significant evidence for concordance or discordance effect directions (fig. S4).

### ME is more likely to modify the genetic effects on neurodevelopment through socioeconomic status rather than family environment

To investigate whether the influence of ME on neurodevelopment could be explained by social/family environments or genetic pathways (*51*), we first examined the association between SNPs with significant SNP×ME effects and educational attainment/ME in a large meta-analysis (*52*). Neither significant associations were observed between these SNPs and educational attainment (table S8) nor ME as well as other related environmental variables, adjusting for age, gender, and population stratification (table S9). We also found no differences in both the SNP×ME effects and the SNP-ME correlations between genetically related and unrelated mother and child dyads (fig. S5). These results suggested that ME is more likely to modify the genetic effects on neurodevelopment through environmental inheritance rather than genetic inheritance. To decompose the multidimensional components of ME, we separately adjusted for socioeconomic and family environmental factors in our GEWIS analysis. We found that most$ of SNPs with significant SNP×ME effects remained significant when adjusting for family environmental factors including family conflict score and parenting monitoring score, while few SNPs remained significant when adjusting for household (household income) and neighborhood socioeconomic status [area deprivation index (ADI)] (Fig. 3C). These results jointly suggested that, compared to micro-family environment, macro-SES may play a more manifested role in modifying the genetic effects on neurodevelopment.

## DISCUSSION

Here, we investigated the interaction effects between SNP/gene and ME on structural brain development using large-scale adolescent cohorts. Eleven independent SNPs with genome-wide significant main or interaction effects were identified, with middle temporal most susceptible to the effect modification by ME. Most loci showing significant interactions with ME also achieved genome-wide significance for main effects on neurodevelopment. Considering that the impacts of these loci on regional GMVs were larger and more significant with lower levels of ME, the diathesis-stress model (*53*, *54*) may be considered in interpreting these G×ME interaction results, especially for those loci with significant main effects. Specific genotypes may work as diatheses by increasing the vulnerability of individuals’ brain development in low ME environments, while higher ME may be interpreted as a protective factor for child’s brain development. These loci were mapped to functional genes involved in synaptic plasticity (i.e., *CACNA1A* and *PTPRD*), metabolic process (i.e., *SORBS1*), and immune process (i.e., *CEBPA* and *OSMR*). Synaptic plasticity refers to the dynamic modulation of the strength of synaptic connections, enabling neural activities generated by the external environment to shape brain morphology and functions (*55*). Previous studies have reported that metabolism played an important role in neuronal development and neural activities. Specifically, glucoses work as a main energy source for neuronal differentiation, morphogenesis, and synaptic function (*56*), while lipids, such as myelin, work as components of cellular structural machinery (*57*) and bioactive molecules (*58*) implicated in neuronal signaling processes (*59*). Meanwhile, immune system is crucial to the production of brain-derived neurotrophic factor (*60*, *61*), survival of multiple neural cells (*62*, *63*), regulation of myelination (*64*, *65*), and formation and pruning of the synapses (*66*, *67*) via diverse pathways including phagocytosis and complement cascade. Other mapped genes were reported to be associated with related phenotypes, including CNS development, neurocognition (such as math capability and memory), psychiatric disorders (such as bipolar and depression), and neurodegenerative disorders (such as AD and Parkinson’s disease).

The GEWIS findings obtained from the ABCD cohort were further validated in both the leave-out sample of ABCD and IMAGEN, an independent and large-scale cohort of adolescents. Eighty-eight percent of the SNPs with significant interaction effects were validated in the internal validation using the leave-out ABCD sample. Because of limited sample size of IMAGEN and potential heterogeneity between the two studies, we adopted a robust validation approach by checking the concordance of effect directions and reduction of *P* values after meta-analyzing both cohorts (*40*). The external validation yielded concordant directions for all significant SNP×ME effects between ABCD and IMAGEN, suggesting replicability of the original GEWIS results. It is important to note that age is crucial for the reproducibility of the GEWIS results: Adolescent GMV changes during this critical period follow a quadratic pattern (*68*); in addition, moderation effects of age in the study of both genetic and environmental influences were highlighted in previous studies (*69*). Besides, gene-based analysis also confirmed the role of *SORBS1* in interacting with ME during brain development. Previous studies suggested that *CSMD1* play critical roles in regulating axonal pruning through phagocytosis (*41*), and consistently, we observed differential effects of *CSMD1* on cortical and regional GMVs (including middle temporal, thalamus, lateral orbitofrontal, and medial orbitofrontal) among adolescents with high ME compared to those with low ME. Notably, these brain regions form the default mode network (DMN) (*70*) and are largely activated in tasks requiring participants to understand and interact with others (*71*). Our results confirmed previous findings that environmental factors, including high levels of income (*72*), ME (*72*), and supportive parental practices (*73*, *74*), could interact with genes in inducing high within-DMN connectivity in children and adolescents (*75*). Further, gene-set analysis strengthened the findings of metabolic and immune alterations as potential interactive pathways where ME could modify structural brain development.

Next, inspired by the Scarr-Rowe hypothesis (*76*), which referred to a better realization of children’s genetic predisposition in resource-rich family environments, we conducted whole-genome analysis to calculate the heritability across different ME levels and found an increase of heritability in high compared to low ME. As previous work has stressed out the importance of using the entire genome to entangle the contribution of gene-environment interactions (*54*), it should be cautious that significant results found in participants with Low-ME cannot be interpreted as higher heritability.

Considerable overlap was observed between genetic variants interacting with ME and those associated with neuropsychiatric/neurological disorders and physical/psychological traits. This indicated that ME could affect neuropsychiatric/neurological disorders and related traits via similar pathways as in the neurodevelopment of corresponding brain regions, highlighting the consideration of environmental background when studying genetic relationships between phenotypes. It has been called genetic correlation–by–environment interactions in previous studies (*77*), referred to changes in pleiotropic effects across different environments. Although marginal associations have been reported between ME and neuropsychiatric/neurological disorders (*10*, *78*–*82*), focusing on brain regions with significant interactions revealed consistent findings with previous studies that these common genetic variants are linked to pathways involved in metabolic and inflammatory disorders, neuropsychiatric disorders, and intelligence.

From the social context, ME reflects multiple environmental dimensions, summarized by family environment and SES. Higher levels of ME demonstrate significant associations with higher family income (*14*, *83*), maternal psychological well-being (*84*), stable family structure, as well as better parenting patterns (*18*). Results from our analysis suggested that SES (household and neighborhood conditions), rather than micro-family environment (such as family conflict and parenting patterns), played a more important role in modifying the genetic effects on neurodevelopment. This finding has important implications for welfare state policy and interventions aimed at addressing the negative effects of social inequality during adolescent brain development. Specifically, adequate efforts could be allocated in improving family income and social housing assistance. However, it did not mean that the role of parent-child communication should be negated. The decomposition of the interaction effects between gene and ME into different environment pathways could be much more complex as shown in previous studies (*51*, *85*, *86*).

There are several limitations associated with our study. First, due to limited availability of adolescent cohorts, sample sizes in both the discovery and validation step of our study remained relatively small compared to existing GWASs, which could lead to decreased statistical power in detecting the true gene-by-environment interaction effects. Moreover, when ME was treated as a continuous variable to increase statistical power, we may overlook the potential departure from linearity. Second, due to our research aim of discovering biological processes that may be influenced by ME, we mainly focused on the SNP-level annotations in this research regardless of the whole-genome information. It should be particularly cautious when interpreting this result in terms of heritability. Third, only genes with the strongest evidence of SNP association were considered in the annotation and interpretation of GEWIS results. Those with moderate or low SNP associations were omitted since the Variant-to-Gene (V2G) evidence score from OpenTarget was mostly derived from a single mapping method, which lacks reliability. Fourth, the overlap between genetic variants that significantly interact with ME on brain development and those significantly correlated with neuropsychiatric/neurological disorders and related traits only demonstrated pleiotropy instead of causal relationships. Future research is needed to explore the causal relationship between these variants and confirm the role of ME on modifying the genetic effects on diseases and related traits. Further, although we did not find any evidence to support correlations between genotypes and ME, the potential existence of gene-environment interactions cannot be ignored (*87*). Trio-based analysis was needed to further elucidate different sources of gene-environment interactions, which was out of our scope. When decomposing components of ME, environment factors in different pathways were adjusted separately in the GEWIS model, ignoring the complex relationships between socioeconomic factors and family environment. More complex covariate adjustment method should be developed to solve this problem.

## MATERIALS AND METHODS

### Data sources

Data from two adolescent cohorts were used throughout this study. The ABCD study was used to identify SNP/gene × ME interaction effects on structural brain development, and the IMAGEN study was used for replication. Detailed descriptions about these two cohorts are described elsewhere (*88*, *89*). Demographic and environmental factors and neuroimaging data from the curated annual ABCD release 2.0 (age 9 to 10 years, *N* = 11,811) and IMAGEN (age at 14 years, *N* = 2082) were used in the analyses. Quality-controlled T1-weighted neuroimaging data were processed using FreeSurfer v6, and regional GMVs were extracted using aparc and aseg atlases. Details on the preprocessing of neuroimaging data can be found in (*90*) for ABCD and at https://github.com/imagen2/imagen_mri for IMAGEN. Participants with regional GMVs beyond 4 Interquartile Ranges (IQRs) were regarded as outliers and excluded from the analyses. Genotype data were quality controlled using PLINK 1.90, where SNPs with call rates <95%, minor allele frequency <1%, and deviation from the Hardy-Weinberg equilibrium with *P* < 1 × 10^−10^ were excluded from the analysis. Because of genetic diversity (*91*) and low linkage disequilibrium (LD) levels of African populations (*92*), a total of 2387 ABCD subjects self-reporting ancestral origins as Black or African American were excluded. Considering that ABCD is oversampled for siblings and twins, and thereby has a nested structure, we randomly selected one participant within a family (the kinship relationship between participants was decided by genetically inferred zygosity status in acspsw03 file). The excluded participants in this step together with their siblings were further used as an internal validation set. Details on preprocessing of the genotype data can be found in (*93*, *94*, *95*). After stringent quality control, a total of 5,020,358 SNPs and 7662 participants in ABCD, and 5,966,316 SNPs and 1982 participants in IMAGEN were included in the final analyses.

### Ethics statement

All the cohort data used in this study comply with relevant ethical regulations. The ABCD study was supported by the National Institutes of Health (NIH), and the IMAGEN study was approved by local ethnical research committees at each research site: King’s College London, University of Nottingham, Trinity College Dublin, University of Heidelberg, Technische Universitat Dresden, Commissariat a l’Energie Atomique et aux Energies Alternatives, and University Medical Center. Informed consent was sought from all participants and a parent/guardian of each participant if under 18 years in all studies.

### Measures of mother-child relationship, SES, and family environment

#### Mother-child relationship

The kinships between mother-child dyads were self-reported. Genetically related mothers were defined by those reported as the child’s biological mother, while non–genetically related mothers were defined by those reported as the child’s adoptive mother, custodial mother, or others.

#### Maternal education

ME was categorized according to the highest degree attained by one’s biological mother. Adolescents whose mother completed a higher professional programs or university programs were coded as High-ME, those whose mother completed a general higher secondary education or equivalent were coded as Medium-ME, and those whose mother was only involved in primary education/lower secondary education were coded as Low-ME. For ABCD, High-ME: some college/associate degree/bachelor’s degree/master’s degree/professional school degree/doctoral degree; Medium-ME: high school graduate/general educational development (GED) or equivalent diploma general; Low-ME: 1st to 12th grade. For IMAGEN, High-ME: professional qualification/bachelor’s degree/advanced diploma; Medium-ME: A levels or a BTEC (Business and Technology Education Council) national diploma/NVQ (National Vocational Qualification) or GNVQ (General National Vocational Qualification); Low-ME: O levels, GCSE (General Certificate of Secondary Education) or CSE (Certificate of Secondary Education)/less than primary school education.

#### Household and neighborhood SES

Household income was defined as the total combined family income for the past 12 months before the investigation. It is recoded as an ordinal variable: 0 for less than 50,000 US dollars, 1 for 50,000 to 100,000 US dollars, and 2 for more than 100,000 US dollars. Neighborhood SES was measured using the ADI, which was calculated based on the participant’s primary residential address. The ADI is based on census data on 17 different factors including income, education, employment, and housing quality and provides rankings of neighborhoods as a national percentile (*96*). Higher ADI reflects greater disadvantages in terms of SES.

#### Family environment

Family conflict score was estimated as the average of nine questions from the ABCD Parent Family Environment Scale-Family Conflict Subscale Modified from PhenX, which assesses conflict between family members, including parents and children (*97*). Participants with more than five missing answers were excluded. Missing values were imputed as the average score across all answered questions. Parental monitoring score was calculated as the average of the five questions from the ABCD Parental Monitoring Survey, which reflects overall high parental monitoring behaviors (*98*).

### Statistical analysis

#### Correlation analysis

To examine the overall impact of ME on brain development, we combined data from ABCD and IMAGEN, and performed linear regression of ME on regional brain GMVs adjusting for age, site, handedness, sex, and estimated intracranial volume. To assess the longitudinal impact of ME on regional GMVs, we adopted a two-stage model fitting approach, where estimated intracranial volume was first estimated using a model linear in age adjusting for sex, site, and handedness, and regional GMVs were then fitted using quadratic term of age and linear age by ME interaction. Model selection was performed using likelihood-ratio test (table S2), and BY-FDR was used to correct for multiple testing.

#### Gene-environment–wide interaction study

To investigate the interactions between genetic variants and ME on brain development, we assumed an additive genetic model where the number of risk alleles was treated as a continuous variable (coded as 0, 1, and 2), and regional brain GMVs were used as the phenotype. The primary analytic model included SNP, ME, and SNP×ME interaction term as variables of interest, and sex, baseline age, estimated intracranial volume, site, handedness, and top *m* principal components of genomic marker variations (*m* = 20) as covariates using PLINK 2 (*99*). To save degrees of freedom and increase the statistical power, ME was also treated as a continuous variable (coded as 0, 1, and 2 for Low-ME, Medium-ME, and High-ME, respectively). Since the genetic effects on brain GMV at Medium-ME (relative to Low-ME) were approximately half of those at High-ME (fig. S6), it is reasonable to treat ME as a continuous variable. A sensitivity analysis for treating ME as a nominal categorical variable was further conducted to test the linearity of genetic effects with varying levels of ME. The main effect of SNP referred to the genetic effects in the population with low-ME (ME = 0). A horizontal shift of ME was applied to estimate the corresponding genetic effects for population with different ME levels. While previous studies have suggested potential confounding effects for G×E terms (*39*), we also included both the covariate-by-environment and the covariate-by-gene interaction term in the GEWIS model as a sensitive analysis. However, the inclusion of multiple interaction effects would make it challenging to interpret both the main genetic effect and G×E results, which cannot be compared with the GEWIS results directly.

Additionally, due to the emphasis on the utilization of whole-genome information (*54*), we calculated the polygenetic score (PGS) using external GWAS (*37*, *38*) [PRSice-2 (*100*)] for ABCD participants. Considering the possible genetic contribution difference in the brain morphology between adolescents and adults, we selected GWAS summary statistics, which was also conducted in adolescents. Thus, only those for subcortical regions found with significant G×ME results and intracranial volume that was used as a measurement for the total brain development were used. The heritability was estimated by the correlation analysis between PGS and the corresponding GMV phenotype adjusted for sex, baseline age, site, handedness, and top 20 principal components of genomic marker variations.

To replicate the GEWIS results, we first conducted an internal validation using sibling pairs in ABCD, where the shared family environment within siblings was modeled as the random effect (lmer 1.1-34 package). The BH-FDR method was used for multiple testing. Next, we performed the same analytic steps in IMAGEN (*m* = 20) as an external replication set and check for the effect concordance on significant interaction effects between ABCD and IMAGEN. Inverse variance weighted meta-analysis was used to detect the concordance of interaction effects between GEWIS_ABCD_ and GEWIS_IMAGEN_. Because the presence of passive gene-environment correlations may influence the estimated G×ME interactions, that is, the child inherits both genotypes and environments from their parents (*87*), Pearson’s correlation analysis was used to test the independence between identified loci with significant G×ME interactions and ME. We also tested for other family environmental factors and socioeconomic factors due to their correlations with ME. The BH-FDR method was used for multiple testing correction.

#### Functional mapping

Significant SNPs in GEWIS were identified based on their *P* values for the main or interaction effects and clumped by LD for independence [*r*^2^ < 0.6 in the 1000 Genomes phase 3 reference (*101*)] within a 250-kb window using PLINK 1.9. SNPs within genes are mapped to genes based on physical positions. For SNPs in intergenic regions, it is challenging to identify possible causal genes underlying association signals requiring a profound understanding of how they alter gene expressions, instead of solely relying on proximity-based approaches. Thus, V2G pipeline from Open Targets Genetics (www.opentargets.org) were used to map them to genes with evidence scores. The V2G model integrates evidence from molecular phenotype quantitative trait loci, chromatin interaction, in silico functional predictions, and distance between the variant and the canonical transcript start site of genes (*102*). Only mapped genes with the strongest evidence score were reported in the interpretation of GEWIS results.

#### Gene-based and gene-set analyses

Gene-based association analyses were performed in MAGMA v1.08 (*103*) in Functional Mapping and Annotation (FUMA) platform (*104*) using G×ME summary statistics from the GEWIS conducted in ABCD. Associations were tested using the SNP-wise mean model, in which the sum of −log (SNP *P* values) for SNPs located within the transcript region was used as the test statistic. LD correction was estimated from the 1000 Genomes phase 3 reference. *P* values from the gene-based association analyses were then used to test whether candidate gene sets belong to specific biological pathways or processes.

#### Multiple testing corrections

As suggested by Vrieze *et al.* (*105*), G×E interactions in association studies were much more difficult to identify than main effects, requiring a sample size of more than 50,000 participants to reach 80% power at the significance threshold of 5 × 10^−8^. Thus, we adopted a two-step approach to increase the statistical power. First, we conducted GEWIS studies in all the brain phenotypes. Then, we applied multiple testing method to only those phenotypes identified with significant G×E effects (*P* < 5 × 10^−8^). As these phenotypes were correlated to some extent, we estimated the effective number of independent variables based on matrix spectral decomposition, using Li and Ji’s method (*106*). Thus, the Bonferroni-adjusted significant thresholds were set at *P* < 1.25 × 10^−8^ (5.0 × 10^−8^/4). For gene-based and gene-set significance, we applied adjusted significance of *P* < 1.25 × 10^−6^ (5.0 × 10^−6^/4) and suggestive significance of *P* < 2.50 × 10^−5^ (1.0 × 10^−4^/4).

#### Genetic overlap

Several important neuropsychiatric and neurological disorders, and physical, psychological, and disease-related phenotypes involved in neurodevelopment were selected according to Brouwer *et al.* (*107*). Summary statistics were obtained from public GWAS for intelligence (*108*), memory (*109*), height (*110*), BMI (*110*), waist circumference–adjusted BMI (*111*), triglyceride (*110*), obesity (*112*), diabetes (*113*), inflammatory bowel disease (*114*), multiple sclerosis (*114*), RA (*115*), aggression (*116*), attention-deficit/hyperactivity disorder (*117*), autism (*118*), depression (*119*), schizophrenia (*120*), bipolar disorder (*121*), AD (*122*), and Parkinson’s disease (*110*). To investigate the genetic overlap between SNPs with significant G×ME effects on regional GMVs and those with strong associations with these traits, we performed iSECA (*123*), which examines pleiotropy and concordance of effect directions between two phenotypes by comparing expected and observed overlap in sets of SNPs with different *P* value thresholds. Binomial exact test was adopted to test for pleiotropy, and Fisher’s exact test was used to test for effect direction concordance. Empirical *P* values were generated through permutation testing over 1000 times, and *P* values for pleiotropy and effect concordance were corrected using the BY-FDR method.

#### Decomposition of ME

As ME could reflect different social and family advantages, we adjusted resource-related factors (ADI and household income) and family relationship–related factors (family conflict score and parenting monitoring score) in the GEWIS model by adding one specific environment factor and the interaction effect with genotype identified in GEWIS_ABCD_. Genome-wide nonsignificance of the G×ME effect after the adjustment of other environmental factors suggests that the moderating effect of ME on genetically influenced brain development could be mediated by the corresponding environmental factor.

## Supplementary Materials

### This PDF file includes:

Figs. S1 to S6 Tables S1 to S9 List of IMAGEN Consortium members References
