# Supplementary material for: Gene-environment interactions in the influence of maternal education on adolescent neurodevelopment using ABCD study
Source: Sci Adv. 2024 Nov 15;10(46):eadp3751. doi: 10.1126/sciadv.adp3751 (PMC11567010; doi:10.1126/sciadv.adp3751)
Supplement: Supplementary file 1 — Figs. S1 to S6 Tables S1 to S9 List of IMAGEN Consortium members References [file sciadv.adp3751_sm.pdf]

Science Advances
AAAS

Supplementary Materials for

Gene-environment interactions in the influence of maternal education on adolescent neurodevelopment using ABCD study

Runye Shi *et al.*

Corresponding author: Xiaolei Lin, xiaoleilin@fudan.edu.cn; Jianfeng Feng, jianfeng64@gmail.com

Sci. Adv. **10**, eadp3751 (2024)
DOI: 10.1126/sciadv.adp3751

This PDF file includes:

Figs. S1 to S6
Tables S1 to S9
List of IMAGEN Consortium members
References

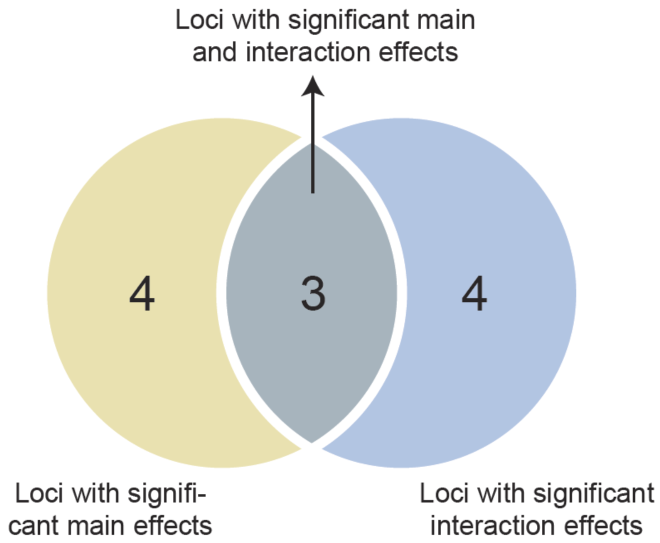

Figure S1. A Venn diagram to illustrate the number of independent loci with significant main/interaction effects and their overlap. A total of 11 independent loci was identified with significant main or interaction effects. There are 7 independent loci with significant main effects and 7 independent loci with significant interaction effects while 3 loci were found with both significant main and interaction effects.

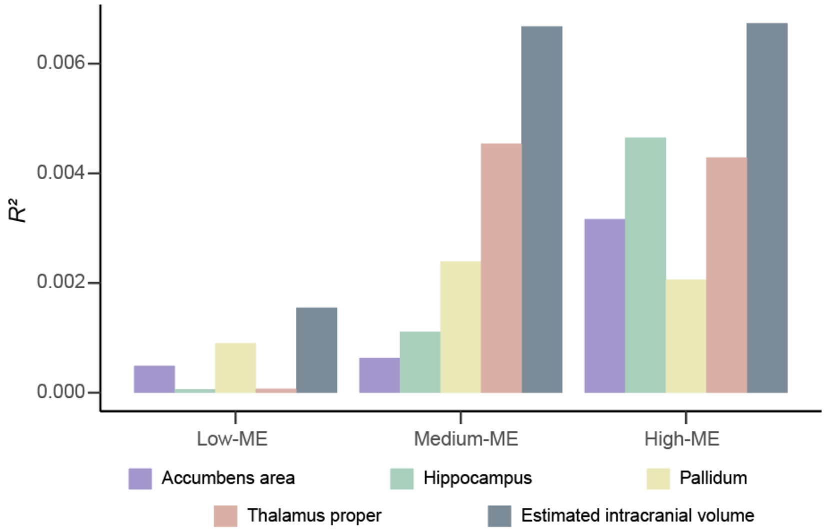

Figure S2. Estimated heritability across different maternal education levels. The R^2 referred to the square of the correlation between PRS and the corresponding brain GMV phenotype.

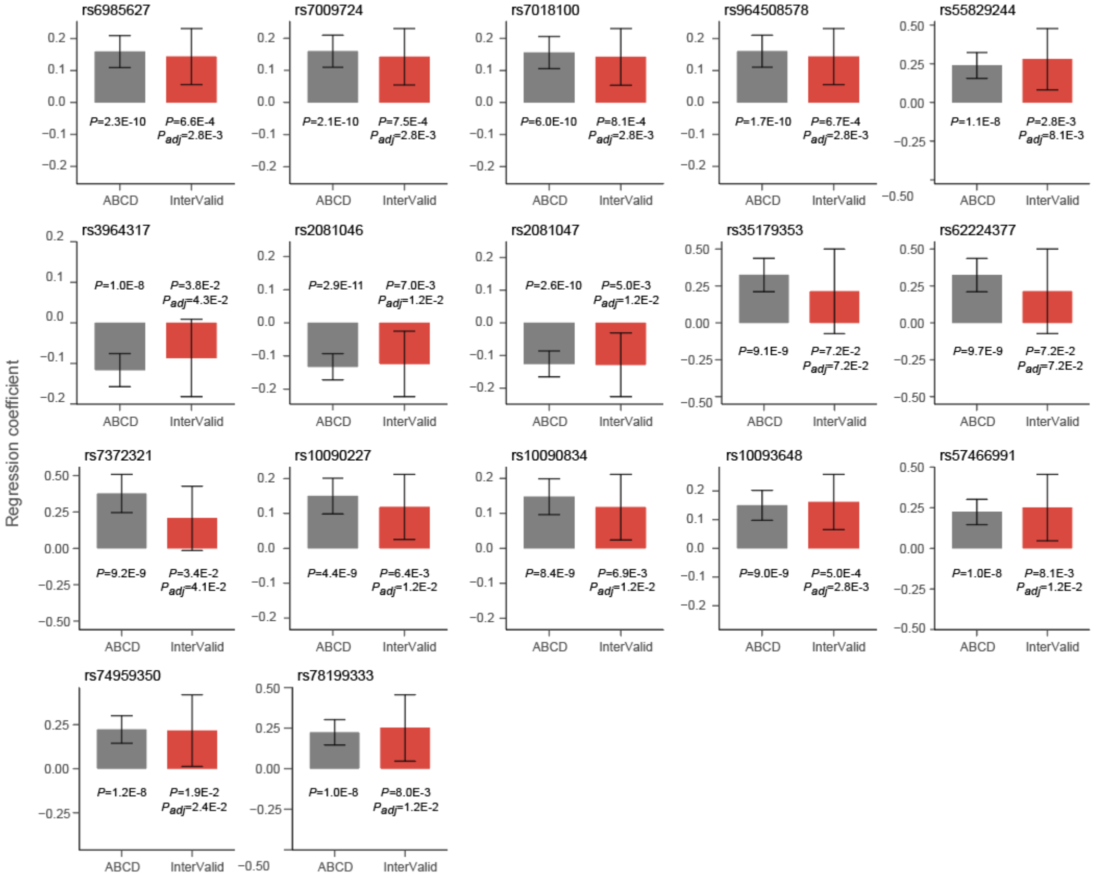

Figure S3. Internal validation of GEWIS results obtained from ABCD using leave-out ABCD samples with their siblings. The error bars indicate 95% confidence intervals of the estimated G×ME interaction effects. BH-FDR method was used for multiple testing correction.

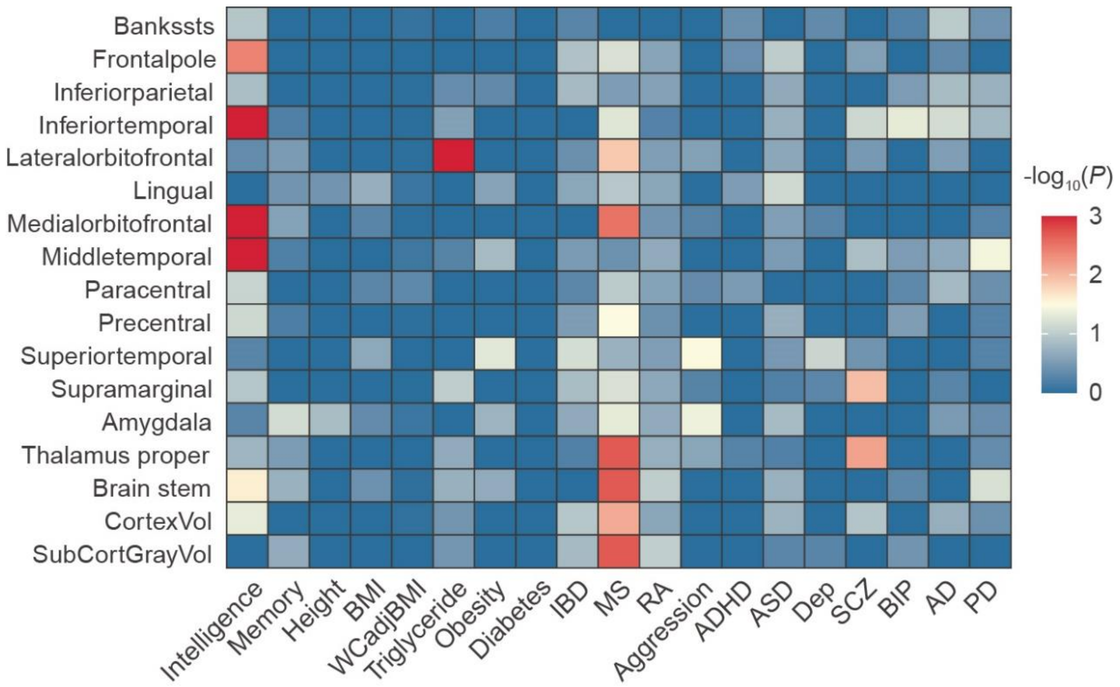

Figure S4. Results from iSECA demonstrating the genetic overlap between the G×ME effects on regional brain GNVs and GWAS summary statistics of multiple phenotypes. BMI, body mass index; WCadjBMI, waist circumference-adjusted BMI; IBD, inflammatory bowel disease; MS, multiple sclerosis; RA, rheumatoid arthritis; ADHD, attention-deficit/hyperactivity disorder; ASD, autism; Dep, depression; SCZ, schizophrenia; BIP, bipolar disorder; AD, Alzheimer’s disease; PD, Parkinson’s disease.

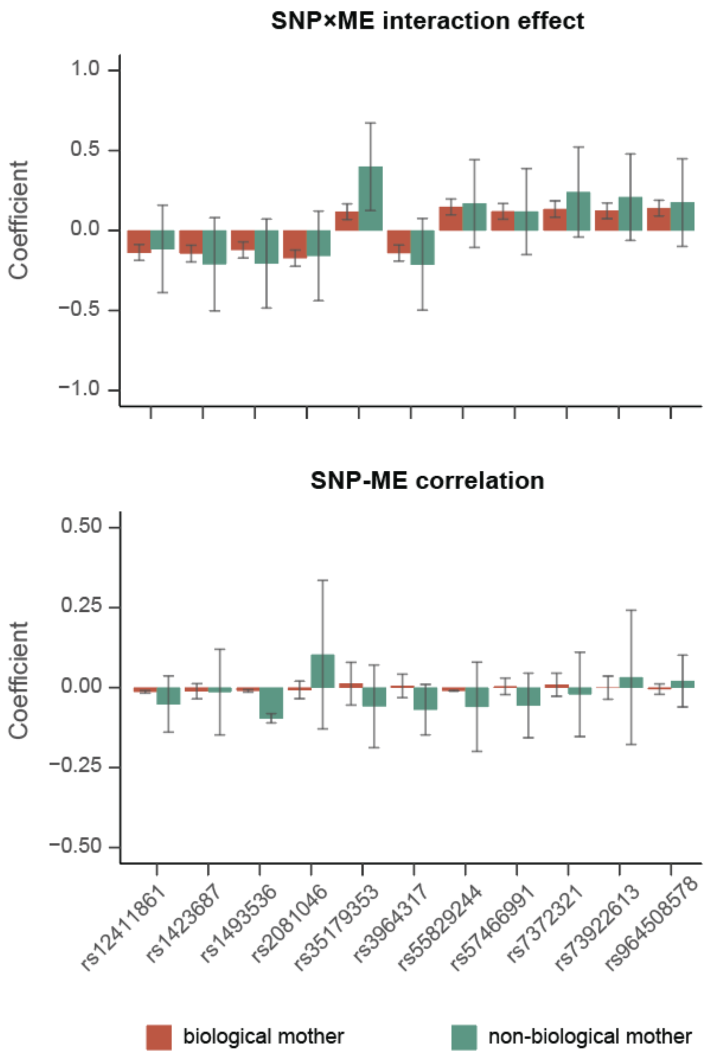

Figure S5. SNP×ME effects and the SNP-ME correlations in genetically related and unrelated mother and child dyads. The error bars indicate 95% confidence intervals of the estimated correlations. No significant differences were found after multiple corrections.

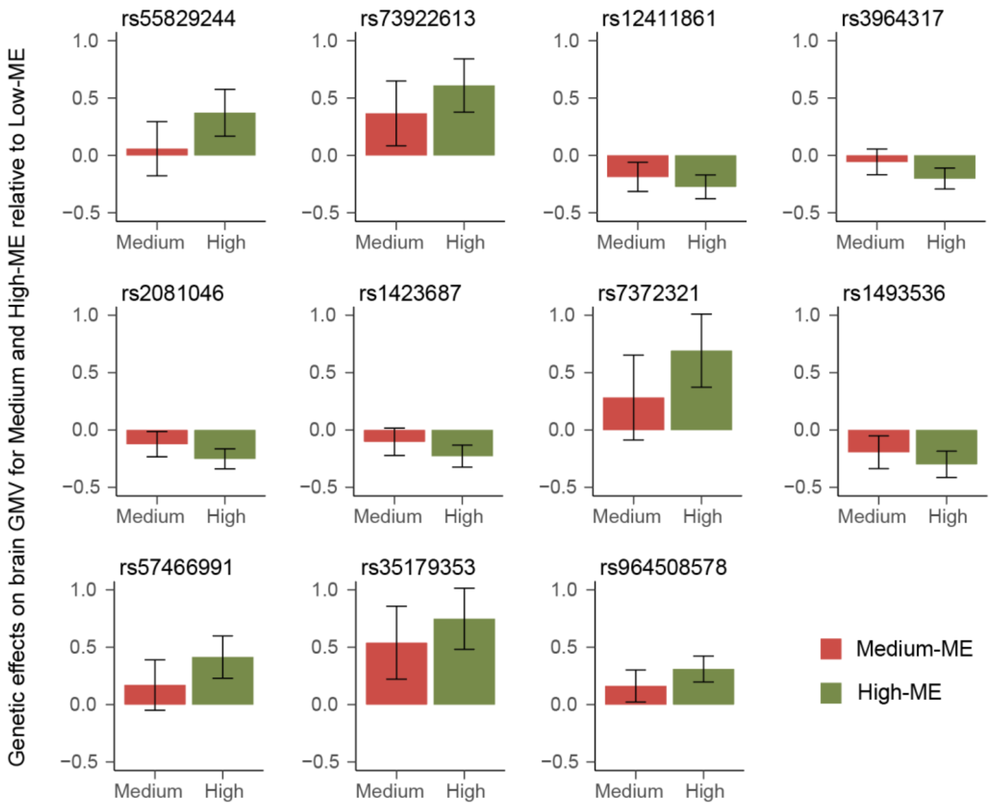

Figure S6. Sensitivity analysis for modeling maternal education as a nominal categorical variable. The error bars indicate 95% confidence intervals of the estimated genetic effects. Loci identified with significant main or interaction effects were selected as examples.

Table S1. Spearman correlation between maternal education and regional brain GMVs.

Brain region	Correlation	P_{adj}
Bankssts	0.037	0.000779***
Caudal anterior cingulate	0.019	1
Caudal middle frontal	0.038	0.000477***
Cuneus	0.019	1
Entorhinal	0.045	5.90E-06***
Fusiform	0.066	7.05E-13***
Inferior parietal	0.031	0.013702*
Inferior temporal	0.062	1.68E-11***
Isthmus cingulate	-0.008	1
Lateral occipital	0.047	1.46E-06***
Lateral orbitofrontal	0.025	0.152179
Lingual	0.025	0.169664
Medial orbitofrontal	0.016	1
Middle temporal	0.074	2.85E-16***
Para hippocampal	0.048	1.32E-06***
Paracentral	0.024	0.235353
Pars opercularis	0.023	0.38425
Pars orbitalis	0.041	7.74E-05***
Pars triangularis	0.008	1
Pericalcarine	0.008	1
Postcentral	0.045	8.44E-06***
Posterior cingulate	0.012	1
Precentral	0.064	4.61E-12***
Precuneus	0.028	0.058982
Rostral anterior cingulate	0.025	0.159312
Rostral middle frontal	0.048	7.26E-07***
Superior frontal	0.046	2.66E-06***
Superior parietal	0.027	0.084431
Superior temporal	0.049	4.90E-07***
Supramarginal	0.035	0.001808**
Frontal pole	0.055	8.43E-09***
Temporal pole	0.047	2.64E-06***
Transverse temporal	0.025	0.178047
Insula	0.031	0.01508*
Accumbens area	0.003	1
Amygdala	0.026	0.121711
Brain stem	0.003	1
Caudate	0.047	2.06E-06***
Pallidum	-0.001	1
Putamen	0.014	1
Hippocampus	0.051	1.14E-07***
Thalamus proper	0.013	1
Cortex	0.085	8.57E-22***
Subcortex	0.047	1.72E-06***

Total brain	0.088	5.03E-23***

All *P* values are adjusted for age, site, handedness, sex and etiv. BY-FDR test was used for multiple testing correction. * $P < 0.05$, ** $P < 0.01$, *** $P < 0.001$

Table S2. Fitting and selection of models examining the longitudinal effect of maternal education on brain development.

**A two-stage model fitting approach**			
Model	AIC	BIC	*P*
The estimation of estimated intracranial volume			
**etiv ~ age + cov**	352539.5	352809.8	
etiv ~ poly(age,2) + cov	352541.1	352818.9	0.514
etiv ~ poly(age,3) + cov	352540.0	352825.3	0.080
The estimation of cortical volume			
CortexVol ~ age + etiv + cov	313736.2	314014.0	
CortexVol ~ age + ME + etiv + cov	313555.9	313848.8	<0.001
CortexVol ~ age + ME + age*ME + etiv + cov	313548.8	313856.6	0.004
**CortexVol ~ poly(age,2) + ME + age*ME + etiv + cov**	313546.7	313863.0	0.035
CortexVol ~ poly(age,2) + ME + poly(age,2)*ME + etiv + cov	313545.9	313876.3	0.058
The estimation of subcortical volume			
Subcortex ~ age + etiv + cov	250681.6	250959.4	
Subcortex ~ age + ME + etiv + cov	250645.4	250938.2	<0.001
**Subcortex ~ age + ME + age*ME + etiv + cov**	250641.9	250949.7	0.023
Subcortex ~ poly(age,2) + ME + age*ME + etiv + cov	250642.5	250954.8	0.356
Subcortex ~ poly(age,2) + ME + poly(age,2)*ME + etiv + cov	250642.1	250959.5	0.075

The default covariates in all the models are handedness, sex and site. *P* value refers to the comparison with the previous model in each part using likelihood-ratio test. **Bold** model refers to the model selected in the fitting process. ME, maternal education.

Table S3. Functional annotations of genomic loci with significant main or interaction effects in GEWIS.

Phenotype	Ind. sig. SNP	Main effect *Beta (P)*	Interaction effect *Beta (P)*	Mapped gene (mapping method)	Annotation
Cuneus	rs7372321	-0.65 (1.84E-07)	0.38 (9.22E-09)	*ADAMTS9-AS2** (position)	*ADAMTS9-AS2* was widely studied in human cancers by suppressing the PI3K/AKT signal pathway(*30, 31*), which was implicated in inflammation(*32*) and the expression of LDHA(*124*), a marker of glycolytic pathway. It was found to be correlated with multiple metabolic markers(*125*), brain morphology(*126*) and neuroticism(*127, 128*).
Lateral orbitofrontal	rs57466991	-0.42 (2.05E-08)	0.22 (1.02E-08)	*ANK1* (position, sQTL, eQTL)	*ANK1* plays an important role in in maintaining the stability of the erythrocyte membrane. Region and disease-specific differential methylation of *ANK1* was observed in multiple neurodegenerative diseases(*129*). It was found to be correlated with type 2 diabetes(*130*) and lymphocyte count(*131*).
Lingual	rs35179353	-0.59 (5.27E-08)	0.32 (9.14E-09)	/	/
Middle temporal	rs964508578	-0.29 (2.13E-09)	0.16 (1.68E-10)	*ERI1** (position)	*ERI1* enables 3'-5' exonuclease activity. It was found to be correlated with BMI-adjusted waist circumference(*132*), BMI(*133*) and neuroticism(*134*).
Middle temporal	rs1493536	0.29 (5.42E-09)	-0.14 (3.87E-08)	*ARHGAP32* (eQTL)	*ARHGAP32*, also known as *RICS*, is a neuron-associated GTPase-activating protein that may regulate dendritic spine morphology and strength by modulating Rho GTPase activity(*135*). It was found to be correlated with height(*136*).
Brain stem	rs55829244	-0.46 (1.01E-08)	0.24 (1.10E-08)	*PTPRD* (position)	*PTPRD* encodes a neuronal cell adhesion molecule and synaptic specifier. Human genetics and mouse models suggested that it was related to addiction, neurofibrillary pathology in Alzheimer's disease, cognitive impairment/intellectual disability, mood lability, and obsessive-compulsive disorder(*137*).
Cortex	rs73922613	-0.57 (5.54E-09)	0.29 (1.26E-08)	*CACNA1A* (position)	This gene encodes the α1A pore-forming subunit of the neuronal calcium channel P/Q(*33*), which are ubiquitous at central synapses and particularly abundant in cerebellar granules and Purkinje cells(*138*). It was found to be correlated with educational attainment(*52*) and brain shape(*139, 140*).
Cortex	rs12411861	0.25 (7.56E-09)	-0.13 (4.62E-08)	*SORBS1* (position)	*SORBS1* encodes a CBL-associated protein which functions in the signaling and stimulation of insulin. It was found to be correlated with IgG glycosylation(*141*) and BMI(*142*).
Cortex	rs3964317	0.22 (1.50E-08)	-0.12 (1.03E-08)	*CDH11* (distance)	*CDH11* encodes a type II classical cadherin that mediate calcium-dependent cell-cell adhesion. It was correlated with altered

					dendritic complexity and neuronal/synaptic activity(*36*). It was found to be correlated with cortical surface area(*143*) and thickness(*144*).
Cortex	rs2081046	0.23 (9.34E-10)	-0.13 (2.87E-11)	*CEBPA* (eQTL, PCHi-C)	*CEBPA* is the CCAAT enhancer binding protein alpha, which encodes a transcription factor that can modulate the expression of genes involved in cell cycle regulation as well as in lipid homeostasis. This gene was found expressed in brain(*145*) and among their target genes are many pro-inflammatory cytokines(*21*).
Cortex	rs1423687	0.23 (7.18E-09)	-0.12 (5.21E-08)	*OSMR* (sQTL)	This gene encodes a member of the type I cytokine receptor family. It was found to be correlated with waist-hip index(*132*) and inflammatory bowel disease(*146*).

Bonferroni-corrected significance threshold was used. We used Variant-to-Gene pipeline (V2G) in Open Targets Genetics to assign variants to genes. / indicates missing data or evidence from Open Target. * indicates that this SNP is not listed in Open Target, but is as an intron variant or within-gene variant in the Single-Nucleotide Polymorphism database (dbSNP). Multiple mapping methods were included in V2G method: *position* = intron variant, *distance* = distance from the variant to the transcription start site of the gene within ±500kb, *pQTL* = linkage to protein abundance in blood plasma, *eQTL* = linkage to changes in gene expression, *PCHi-C* = linkage using Promotor Capture Hi-C in Javierre et al.(*147*). The annotation contains descriptions about function of encoded product and brain-plasticity-related or metabolism/immune-related phenotype associations.

Table S4. The GEWIS results when both the covariates by G and covariates by E terms were included.

SNP	Original GEWIS model		New GEWIS model	
	G (*P*)	G×ME (*P*)	G (P_{adj})	G×ME (P_{adj})
rs6985627	-0.3 (2.6E-9)	0.2 (2.3E-10)	-6E-17 (0.62)	9E-18 (0.04)
rs7009724	-0.3 (2.7E-9)	0.2 (2.1E-10)	4E-17 (0.69)	-9E-18 (0.04)
rs7018100	-0.3 (5.3E-9)	0.2 (6.0E-10)	3E-16 (0.44)	4E-17 (0.05)
rs964508578	-0.3 (2.1E-9)	0.2 (1.7E-10)	1E-16 (0.89)	8E-17 (3.2E-4)
rs55829244	-0.5 (1.0E-8)	0.2 (1.1E-8)	2E-16 (0.44)	-5E-17 (0.02)
rs3964317	0.2 (1.5E-8)	-0.1 (1.0E-8)	-2E-16 (0.44)	-2E-17 (0.04)
rs2081046	0.2 (9.3E-10)	-0.1 (2.9E-11)	-3E-17 (0.90)	4E-17 (1.6E-5)
rs2081047	0.2 (6.3E-9)	-0.1 (2.6E-10)	1E-16 (0.90)	2E-16 (4.1E-5)
rs7372321	-0.7 (1.8E-7)	0.4 (9.2E-9)	-3E-16 (0.62)	1E-16 (1.6E-5)
rs10090227	-0.3 (2.9E-8)	0.1 (4.4E-9)	-9E-16 (0.44)	2E-16 (1.0E-5)
rs10090834	-0.3 (4.5E-8)	0.1 (8.4E-9)	2E-16 (0.61)	-4E-17 (6.8E-4)
rs10093648	-0.3 (1.1E-7)	0.1 (9.0E-9)	-6E-18 (0.94)	2E-17 (0.024)
rs57466991	-0.4 (2.1E-8)	0.2 (1.0E-8)	-4E-17 (0.90)	-3E-17 (8.6E-3)
rs74959350	-0.4 (2.2E-8)	0.2 (1.2E-8)	-1E-16 (0.92)	-1E-16 (2.2E-2)
rs78199333	-0.4 (2.1E-8)	0.2 (1.0E-8)	-3E-16 (0.62)	8E-17 (0.04)
rs35179353	-0.6 (5.3E-8)	0.3 (9.1E-9)	-4E-17 (0.93)	-5E-17 (0.07)
rs62224377	-0.6 (5.6E-8)	0.3 (9.7E-9)	-4E-16 (0.87)	2E-16 (1.4E-3)

Bonferroni-corrected significance thresholds were used for original GEWIS model. *P* values in the new GEWIS model were adjusted using the BH-FDR method.

Table S5. Meta-analysis of GEWIS in ABCD and IMAGEN.

Phenotype	SNP	$GEWIS_{ABCD}$ *Beta (P)*	$GEWIS_{IMAGEN}$ *Beta (P)*	Meta-analysis *Beta (P)*
Cuneus	rs7372321	0.38 (9.22E-09)	0.03 (6.70E-01)	0.20 (7.24E-09)
Lateral orbitofrontal	rs78199333	0.22 (1.04E-08)	0.04 (3.51E-01)	0.15 (3.60E-09)
Lateral orbitofrontal	rs74959350	0.22 (1.21E-08)	0.04 (3.51E-01)	0.15 (4.18E-09)
Lateral orbitofrontal	rs57466991	0.22 (1.02E-08)	0.04 (3.47E-01)	0.15 (3.51E-09)
Lingual	rs35179353	0.32 (9.14E-09)	0.04 (4.33E-01)	0.18 (3.97E-09)
Lingual	rs62224377	0.32 (9.72E-09)	0.06 (3.06E-01)	0.19 (2.98E-09)
Middle temporal	rs10090227	0.15 (4.37E-09)	0.07 (4.37E-02)	0.13 (2.96E-10)
Middle temporal	rs10093648	0.15 (9.00E-09)	0.06 (1.28E-01)	0.12 (1.32E-09)
Middle temporal	rs10090834	0.15 (8.44E-09)	0.08 (3.94E-02)	0.12 (5.43E-10)
Middle temporal	rs7018100	0.16 (6.04E-10)	0.07 (6.33E-02)	0.13 (5.10E-11)
Middle temporal	rs7009724	0.16 (2.09E-10)	0.07 (5.86E-02)	0.13 (1.64E-11)
Middle temporal	rs964508578	0.16 (1.68E-10)	0.07 (5.93E-02)	0.13 (1.33E-11)
Middle temporal	rs6985627	0.16 (2.29E-10)	0.07 (6.37E-02)	0.13 (1.92E-11)
Brain stem	rs55829244	0.24 (1.10E-08)	0.07 (1.27E-01)	0.17 (1.60E-09)
Cortex	rs3964317	-0.12 (1.03E-08)	-0.05 (8.51E-02)	-0.1 (1.10E-09)
Cortex	rs2081047	-0.13 (2.58E-10)	-0.07 (3.45E-02)	-0.11 (1.48E-11)
Cortex	rs2081046	-0.13 (2.87E-11)	-0.06 (9.63E-02)	-0.12 (3.26E-12)

17 SNPs with significant G×ME effects identified in the ABCD study are shown in the table. Bonferroni-corrected significance thresholds were used.

Table S6. Genes showing genome-wide significant interaction effects with maternal education on regional brain GMVs, revealed using gene-based association analysis.

Phenotype	Gene	*P*	Annotation
Caudal middle frontal	*HRC*	1.10E-06	*HRC* encodes a luminal sarcoplasmic reticulum protein. It was found to be correlated with BMI(*142*).
Inferior parietal	*AHSA1*	1.39E-07	*AHSA1* enables ATPase activator activity.
Medial orbitofrontal	*CTNNA2*	7.28E-07	*CTNNA2* encodes a cell adhesion protein known as αN-catenin, which is predominately expressed in the CNS especially the prefrontal cortex. This protein is crucial for the maintenance of dendritic spine and synaptic connection stability(*47-49*). It has been reported to be correlated with attention-deficit/hyperactivity disorder(*148*) and multiple psychiatric disorders such as schizophrenia(*149*) and bipolar disorder(*150*).
Middle temporal	*CSMD1*	5.39E-07	*CSMD1* was correlated with working memory, episodic memory and other cognitive functions(*45, 46*).
Posterior cingulate	*ANK3*	7.48E-07	*ANK3* was originally found at the axonal initial segment and nodes of Ranvier of neurons in the central and peripheral nervous systems. It was found to be correlated with multiple neuropsychiatric disorders(*151-153*).
Superior frontal	*PLEKHG1*	5.14E-07	*PLEKHG1* was predicted to be involved in regulation of small GTPase mediated signal transduction. It was found to be correlated with cortical thickness/surface area(*144*), white matter hyperintensities(*154*) and obesity-related traits(*155*).
Cortex	*CSMD1*	3.99E-12	See above.
Cortex	*ANK2*	1.59E-07	*ANK2* encodes a major ankyrin-B polypeptides required for normal structural connectivity in CNS(*50*)
Cortex	*CTNNA2*	6.89E-07	See above.
Cortex	*UBN2*	7.97E-07	/
Subcortex	*CSMD1*	2.36E-07	See above.

Bonferroni-corrected significance threshold was used. The annotation contains descriptions about function of encoded product and brain-plasticity-related or metabolism/immune-related phenotype associations.

Table S7. Gene set analysis revealed biological pathways involved in the interaction with maternal education on regional brain GMVs.

Phenotype	Gene-set	# genes	*P*	Short description
Caudal middle frontal	GOBP_chemorepulsion_of_axon	6	1.03E-06	The process in which a neuron growth cone is guided to a specific target site in response to a repulsive chemical signal implicated in accurate synaptogenesis.
Inferior parietal	GOBP_glycolipid_transport	7	6.21E-08	The directed movement process of glycolipids.
Inferior temporal	GOBP_go_negative_regulation_of_mhc_class_ii_biosynthetic_process	5	9.66E-07	Any process that stops, prevents, or reduces the frequency, rate or extent of the chemical reactions and pathways resulting in the formation of MHC class II.
Lateral occipital	GOBP_tachykinin_receptor_signaling_pathway	8	9.49E-07	A signaling pathway initiated by the binding of tachykinin to its receptor, which are expressed throughout the nervous and immune systems.
Lingual	GOBP_farnesyl_diphosphate_metabolic_process	6	1.25E-06	Any processes involving farnesyl diphosphate, a key intermediate of sterol metabolism.
Posterior cingulate	Curated_gene_sets: ger1y_cebp_targets	103	3.15E-07	Genes changed in NIH 3T3 cells (embryonic fibroblast) by expression of one or more of C/EBP proteins: CEBPA, CEBPB, CEBPG, and CEBPD.
Putamen	Curated_gene_sets: lindgren_bladder_cancer_high_recurrence	43	1.06E-07	Genes up-regulated among the high recurrence rate urothelial cell carcinoma (UCC) tumors.
Accumbens area	GOBP_regulation_of_mast_cell_activation_involved_in_immune_response	27	1.21E-06	Any process modulating the frequency or extent of mast cell activation in immune response.

The short descriptions of gene set were obtained from Gene Set Enrichment Analysis(*156*) (GSEA) (https://www.gsea-msigdb.org/gsea/index.jsp).

Table S8. Associations between SNPs with significant G×ME effects and education attainment in Lee JJ, et al(*52*).

Phenotype	Sig. SNP	Association	*P*
Cuneus	rs7372321	/	/
Lateral orbitofrontal	rs57466991	1.57E-03	0.468
Lateral orbitofrontal	rs78199333	1.74E-03	0.422
Lateral orbitofrontal	rs74959350	1.67E-03	0.441
Lingual	rs35179353	6.65E-03	0.008
Lingual	rs62224377	6.50E-03	0.009
Middle temporal	rs964508578	/	/
Middle temporal	rs7009724	-3.00E-04	0.860
Middle temporal	rs6985627	-3.00E-04	0.862
Middle temporal	rs7018100	-1.06E-03	0.533
Middle temporal	rs10090227	-8.20E-04	0.630
Middle temporal	rs10090834	-8.30E-04	0.624
Middle temporal	rs10093648	-1.34E-03	0.432
Brain stem	rs55829244	-2.14E-03	0.383
CortexVol	rs2081046	1.93E-03	0.340
CortexVol	rs2081047	1.02E-03	0.605
CortexVol	rs3964317	-3.61E-03	0.046

Table S9. Associations between SNPs (with significant G×ME effects) and maternal education and other environmental factors in ABCD.

Phenotype	Sig. SNP	Correlation (P_{adj})				
		Maternal Education	Family conflict score	Parent monitoring score	Household income	Area Deprivation Index
Cuneus	rs7372321	0.014 (0.874)	0.028 (0.185)	-0.010 (0.606)	-0.020 (0.446)	-0.007 (0.797)
Lateral orbitofrontal	rs57466991	-0.003 (0.874)	-0.023 (0.185)	-0.002 (0.886)	0.009 (0.557)	-0.009 (0.797)
Lateral orbitofrontal	rs78199333	-0.002 (0.874)	-0.023 (0.185)	-0.003 (0.884)	0.010 (0.505)	-0.010 (0.797)
Lateral orbitofrontal	rs74959350	-0.002 (0.874)	-0.023 (0.185)	-0.002 (0.884)	0.010 (0.505)	-0.010 (0.797)
Lingual	rs35179353	0.014 (0.874)	-0.015 (0.388)	0.013 (0.606)	0.006 (0.69)	-0.003 (0.797)
Lingual	rs62224377	0.013 (0.874)	-0.015 (0.388)	0.014 (0.606)	0.005 (0.725)	-0.003 (0.797)
Middle temporal	rs964508578	-0.010 (0.874)	0.006 (0.723)	-0.010 (0.606)	-0.012 (0.505)	-0.006 (0.797)
Middle temporal	rs7009724	-0.010 (0.874)	0.005 (0.723)	-0.010 (0.606)	-0.012 (0.505)	-0.006 (0.797)
Middle temporal	rs6985627	-0.010 (0.874)	0.006 (0.723)	-0.010 (0.606)	-0.012 (0.505)	-0.006 (0.797)
Middle temporal	rs7018100	-0.008 (0.874)	0.006 (0.723)	-0.013 (0.606)	-0.014 (0.505)	-0.007 (0.797)
Middle temporal	rs10090227	-0.008 (0.874)	0.005 (0.723)	-0.016 (0.606)	-0.018 (0.446)	-0.004 (0.797)
Middle temporal	rs10090834	-0.007 (0.874)	0.007 (0.723)	-0.016 (0.606)	-0.019 (0.446)	-0.005 (0.797)
Middle temporal	rs10093648	-0.005 (0.874)	0.004 (0.755)	-0.018 (0.606)	-0.015 (0.505)	-0.009 (0.797)
Brain stem	rs55829244	-0.004 (0.874)	0.006 (0.723)	0.011 (0.606)	0.019 (0.446)	-0.021 (0.501)
CortexVol	rs2081046	-0.004 (0.874)	0.018 (0.364)	0.009 (0.606)	0.004 (0.794)	0.015 (0.668)
CortexVol	rs2081047	-0.006 (0.874)	0.016 (0.388)	0.010 (0.606)	0.002 (0.844)	0.015 (0.668)
CortexVol	rs3964317	0.011 (0.874)	-0.005 (0.723)	0.009 (0.635)	0.015 (0.505)	-0.022 (0.501)

We used Pearson’s correlation analysis to assess the association between maternal education and the identified independent loci with significant G×ME effects, adjusting for sex, handedness, site and top 20 PCA components of genomic marker variations. BH-FDR method was used for multiple testing correction.

Consortium information and authors

A full list of IMAGEN Consortium members: Tobias Banaschewski (Heidelberg University), Gareth J. Barker (King's College London), Arun L.W. Bokde (Trinity College Dublin), Sylvane Desrivières (King's College London), Herta Flor (Heidelberg University; University of Mannheim), Antoine Grigis (Université Paris-Saclay), Hugh Garavan (University of Vermont), Penny Gowland (University of Nottingham), Andreas Heinz (Charité-Universitätsmedizin Berlin), Rüdiger Brühl (Physikalisch-Technische Bundesanstalt), Jean-Luc Martinot (Institut National de la Santé et de la Recherce Médicale; Université Paris-Saclay), Marie-Laure Paillère Martinot (Institut National de la Santé et de la Recherce Médicale; Université Paris-Saclay; Pitié-Salpêtrière Hospital), Eric Artiges (Institut National de la Santé et de la Recherce Médicale; Université Paris-Saclay), Frauke Nees (Heidelberg University), Dimitri Papadopoulos Orfanos (Université Paris-Saclay), Herve Lemaitre (Université Paris-Saclay; Université de Bordeaux), Tomáš Paus (University of Montreal; University of Toronto), Luise Poustka (University Hospital Heidelberg), Sarah Hohman (Heidelberg University), Nathalie Holz (Heidelberg University), Christian Baeuchl (Technische Universität Dresden), Michael N. Smolka (Technische Universität Dresden), Nilakshi Vaidya (Charité Universitätsmedizin Berlin), Henrik Walter (Charité – Universitätsmedizin Berlin), Robert Whelan (Trinity College Dublin), Gunter Schumann (Charité Universitätsmedizin Berlin; Fudan University)

REFERENCES AND NOTES

1. J. S. Peper, H. G. Schnack, R. M. Brouwer, G. C. Van Baal, E. Pjetri, E. Szekely, M. van Leeuwen, S. M. van den Berg, D. L. Collins, A. C. Evans, D. I. Boomsma, R. S. Kahn, H. E. Hulshoff Pol, Heritability of regional and global brain structure at the onset of puberty: A magnetic resonance imaging study in 9-year-old twin pairs. *Hum. Brain Mapp.* **30**, 2184–2196 (2009).

2. K. G. Noble, S. M. Houston, N. H. Brito, H. Bartsch, E. Kan, J. M. Kuperman, N. Akshoomoff, D. G. Amaral, C. S. Bloss, O. Libiger, N. J. Schork, S. S. Murray, B. J. Casey, L. Chang, T. M. Ernst, J. A. Frazier, J. R. Gruen, D. N. Kennedy, P. Van Zijl, S. Mostofsky, W. E. Kaufmann, T. Kenet, A. M. Dale, T. L. Jernigan, E. R. Sowell, Family income, parental education and brain structure in children and adolescents. *Nat. Neurosci.* **18**, 773–778 (2015).

3. K. G. Noble, S. M. Houston, E. Kan, E. R. Sowell, Neural correlates of socioeconomic status in the developing human brain. *Dev. Sci.***15**, 516–527 (2012).

4. Y. Zhou, H. G. Muller, C. Zhu, Y. Chen, J. L. Wang, J. O'Muircheartaigh, M. Bruchhage, S. Deoni; RESONANCE Consortium, Network evolution of regional brain volumes in young children reflects neurocognitive scores and mother's education. *Sci. Rep.* **13**, 2984 (2023).

5. K. Patra, M. M. Greene, A. L. Patel, P. Meier, Maternal education level predicts cognitive, language, and motor outcome in preterm infants in the second year of life. *Am. J. Perinatol.* **33**, 738–744 (2016).

6. R. E. Lean, C. D. Smyser, R. G. Brady, R. L. Triplett, S. Kaplan, J. K. Kenley, J. S. Shimony, T. A. Smyser, J. P. Miller, D. M. Barch, J. L. Luby, B. B. Warner, C. E. Rogers, Prenatal exposure to maternal social disadvantage and psychosocial stress and neonatal white matter connectivity at birth. *Proc. Natl. Acad. Sci. U.S.A.* **119**, e2204135119 (2022).

7. L. C. H. Fernald, P. Kariger, M. Hidrobo, P. J. Gertler, Socioeconomic gradients in child development in very young children: Evidence from India, Indonesia, Peru, and Senegal. *Proc. Natl. Acad. Sci. U.S.A.* **109**, 17273–17280 (2012).

8. L. G. Speyer, H. A. Hall, Y. Hang, C. Hughes, A. L. Murray, Within-family relations of mental health problems across childhood and adolescence. *J. Child Psychol. Psychiatry* **63**, 1288–1296 (2022).

9. L. B. Sherar, T. P. Griffin, U. Ekelund, A. R. Cooper, D. W. Esliger, E. M. van Sluijs, L. Bo Andersen, G. Cardon, R. Davey, K. Froberg, P. C. Hallal, K. F. Janz, K. Kordas, S. Kriemler, R. R. Pate, J. J. Puder, L. B. Sardinha, A. F. Timperio, A. S. Page, Association between maternal education and objectively measured physical activity and sedentary time in adolescents. *J. Epidemiol. Community Health* **70**, 541–548 (2016).

10. D. Fakhrunnisak, B. Patria, The positive effects of parents' education level on children's mental health in Indonesia: A result of longitudinal survey. *BMC Public Health* **22**, 949 (2022).

11. A. K. Meyrose, F. Klasen, C. Otto, G. Gniewosz, T. Lampert, U. Ravens-Sieberer, Benefits of maternal education for mental health trajectories across childhood and adolescence. *Soc. Sci. Med.* **202**, 170–178 (2018).

12. P. Lundborg, A. Nilsson, D.-O. Rooth, Parental education and offspring outcomes: Evidence from the swedish compulsory school reform. *Am. Econ. J. Appl. Econ.* **6**, 253–278 (2014).

13. G. S. Becker, *Human Capital: A Theoretical and Empirical Analysis, with Special Reference to Education* (National Bureau of Economic Research, 1964).

14. N. Krieger, D. R. Williams, N. E. Moss, Measuring social class in US public health research: Concepts, methodologies, and guidelines. *Annu. Rev. Public Health* **18**, 341–378 (1997).

15. M. Jackson, K. Kiernan, S. McLanahan, Maternal education, changing family circumstances, and children's skill development in the United States and UK. *Ann. Am. Acad. Pol. Soc. Sci.* **674**, 59–84 (2017).

16. M. Bornstein, C. S. Hahn, J. Suwalsky, O. Haynes, Socioeconomic status, parenting and child development: The hollingshead fourfactor index of social status and the socioeconomic index of occupations. *Mahwah N J* , 29–82 (2003).

17. R. Hosokawa, T. Katsura, A longitudinal study of socioeconomic status, family processes, and child adjustment from preschool until early elementary school: The role of social competence. *Child Adolesc. Psychiatry Ment. Health* **11**, 62 (2017).

18. J. Belsky, B. Bell, R. H. Bradley, N. Stallard, S. L. Stewart-Brown, Socioeconomic risk, parenting during the preschool years and child health age 6 years. *Eur. J. Public Health* **17**, 508–513 (2007).

19. F. R. Wendt, G. A. Pathak, D. F. Levey, Y. Z. Nunez, C. Overstreet, C. Tyrrell, K. Adhikari, F. De Angelis, D. S. Tylee, A. Goswami, J. H. Krystal, C. G. Abdallah, M. B. Stein, H. R. Kranzler, J. Gelernter, R. Polimanti, Sex-stratified gene-by-environment genome-wide interaction study of trauma, posttraumatic-stress, and suicidality. *Neurobiol. Stress* **14**, 100309 (2021).

20. L. E. Olofsson, M. Orho-Melander, L. William-Olsson, K. Sjoholm, L. Sjostrom, L. Groop, B. Carlsson, L. M. Carlsson, B. Olsson, CCAAT/enhancer binding protein alpha (C/EBPalpha) in adipose tissue regulates genes in lipid and glucose metabolism and a genetic variation in C/EBPalpha is associated with serum levels of triglycerides. *J. Clin. Endocrinol. Metab.* **93**, 4880–4886 (2008).

21. D. P. Ramji, P. Foka, CCAAT/enhancer-binding proteins: Structure, function and regulation. *Biochem. J.* **365**, 561–575 (2002).

22. D. E. Zhang, P. Zhang, N. D. Wang, C. J. Hetherington, G. J. Darlington, D. G. Tenen, Absence of granulocyte colony-stimulating factor signaling and neutrophil development in CCAAT enhancer binding protein alpha-deficient mice. *Proc. Natl. Acad. Sci. U.S.A.* **94**, 569–574 (1997).

23. H. Matsushita, H. Nakajima, Y. Nakamura, H. Tsukamoto, Y. Tanaka, G. Jin, M. Yabe, S. Asai, R. Ono, T. Nosaka, K. Sugita, A. Morimoto, Y. Hayashi, T. Hotta, K. Ando, H. Miyachi, C/EBPalpha and C/EBPvarepsilon induce the monocytic differentiation of myelomonocytic cells with the MLL-chimeric fusion gene. *Oncogene* **27**, 6749–6760 (2008).

24. T.-J. Chang, W.-C. Wang, C. A. Hsiung, C.-T. He, M. W. Lin, W. H.-H. Sheu, Y.-C. Chang, T. Quertermous, Y.-I. Chen, J. I. Rotter, L.-M. Chuang; SAPPHIRe Study Group, Genetic variation of SORBS1 gene is associated with glucose homeostasis and age at onset of diabetes: A SAPPHIRe Cohort Study. *Sci. Rep.* **8**, 10574 (2018).

25. M. R. Irvin, D. Zhi, S. Aslibekyan, S. A. Claas, D. M. Absher, J. M. Ordovas, H. K. Tiwari, S. Watkins, D. K. Arnett, Genomics of post-prandial lipidomic phenotypes in the Genetics of Lipid lowering Drugs and Diet Network (GOLDN) study. *PLOS ONE* **9**, e99509 (2014).

26. N. Wang, Y. Gu, L. Li, J. Chi, X. Liu, Y. Xiong, C. Zhong, Development and validation of a prognostic classifier based on lipid metabolism-related genes for breast cancer. *J. Inflamm. Res.* **15**, 3477–3499 (2022).

27. D. Vdovenko, M. Bachmann, W. J. Wijnen, M. O. Hottiger, U. Eriksson, A. Valaperti, The adaptor protein c-Cbl-associated protein (CAP) limits pro-inflammatory cytokine expression by inhibiting the NF-κB pathway. *Int. Immunopharmacol.* **87**, 106822 (2020).

28. F. Lantieri, T. Bachetti, OSM/OSMR and interleukin 6 family cytokines in physiological and pathological condition. *Int. J. Mol. Sci.* **23**, 11096 (2022).

29. G. Dey, A. Radhakrishnan, N. Syed, J. K. Thomas, A. Nadig, K. Srikumar, P. P. Mathur, A. Pandey, S.-K. Lin, R. Raju, T. S. K. Prasad, Signaling network of Oncostatin M pathway. *J. Cell Commun. Signal.* **7**, 103–108 (2013).

30. S. Xie, X. Yu, Y. Li, H. Ma, S. Fan, W. Chen, G. Pan, W. Wang, H. Zhang, J. Li, Z. Lin, Upregulation of lncRNA ADAMTS9-AS2 promotes salivary adenoid cystic carcinoma metastasis via PI3K/Akt and MEK/Erk signaling. *Mol. Ther.* **26**, 2766–2778 (2018).

31. W. Xu, B. Wang, Y. Cai, J. Chen, X. Lv, C. Guo, C. Yuan, ADAMTS9-AS2: A functional long non-coding rna in tumorigenesis. *Curr. Pharm. Des.* **27**, 2722–2727 (2021).

32. P. T. Hawkins, L. R. Stephens, PI3K signalling in inflammation. *Biochim. Biophys. Acta* **1851**, 882–897 (2015).

33. W. A. Catterall, Voltage-gated sodium channels at 60: Structure, function and pathophysiology. *J. Physiol.* **590**, 2577–2589 (2012).

34. A. A. Guerin, A. Feigenbaum, E. J. Donner, G. Yoon, Stepwise developmental regression associated with novel CACNA1A mutation. *Pediatr. Neurol.* **39**, 363–364 (2008).

35. E. Indelicato, W. Nachbauer, E. Karner, A. Eigentler, M. Wagner, I. Unterberger, W. Poewe, M. Delazer, S. Boesch, The neuropsychiatric phenotype in CACNA1A mutations: A retrospective single center study and review of the literature. *Eur. J. Neurol.* **26**, 66-e7 (2019).

36. J. A. Frei, R. F. Niescier, M. S. Bridi, M. Durens, J. E. Nestor, M. B. C. Kilander, X. Yuan, D. M. Dykxhoorn, M. W. Nestor, S. Huang, G. J. Blatt, Y.-C. Lin, Regulation of neural circuit development by Cadherin-11 provides implications for autism. *eNeuro* **8**, ENEURO.0066-21.2021 (2021).

37. D. P. Hibar, J. L. Stein, M. E. Renteria, A. Arias-Vasquez, S. Desrivieres, N. Jahanshad, R. Toro, K. Wittfeld, L. Abramovic, M. Andersson, B. S. Aribisala, N. J. Armstrong, M. Bernard, M. M. Bohlken, M. P. Boks, J. Bralten, A. A. Brown, M. M. Chakravarty, Q. Chen, C. R. K. Ching, G. Cuellar-Partida, A. den Braber, S. Giddaluru, A. L. Goldman, O. Grimm, T. Guadalupe, J. Hass, G. Woldehawariat, A. J. Holmes, M. Hoogman, D. Janowitz, T. Jia, S. Kim, M. Klein, B. Kraemer, P. H. Lee, L. M. Olde Loohuis, M. Luciano, C. Macare, K. A. Mather, M. Mattheisen, Y. Milaneschi, K. Nho, M. Papmeyer, A. Ramasamy, S. L. Risacher, R. Roiz-Santianez, E. J. Rose, A. Salami, P. G. Samann, L. Schmaal, A. J. Schork, J. Shin, L. T. Strike, A. Teumer, M. M. J. van Donkelaar, K. R. van Eijk, R. K. Walters, L. T. Westlye, C. D. Whelan, A. M. Winkler, M. P. Zwiers, S. Alhusaini, L. Athanasiu, S. Ehrlich, M. M. H. Hakobjan, C. B. Hartberg, U. K. Haukvik, A. J. G. A. M. Heister, D. Hoehn, D. Kasperaviciute, D. C. M. Liewald, L. M. Lopez, R. R. R. Makkinje, M. Matarin, M. A. M. Naber, D. R. McKay, M. Needham, A. C. Nugent, B. Putz, N. A. Royle, L. Shen, E. Sprooten, D. Trabzuni, S. S. L. van der Marel, K. J. E. van Hulzen, E. Walton, C. Wolf, L. Almasy, D. Ames, S. Arepalli, A. A. Assareh, M. E. Bastin, H. Brodaty, K. B. Bulayeva, M. A. Carless, S. Cichon, A. Corvin, J. E. Curran, M. Czisch, G. I. de Zubicaray, A. Dillman, R. Duggirala, T. D. Dyer, S. Erk, I. O. Fedko, L. Ferrucci, T. M. Foroud, P. T. Fox, M. Fukunaga, J. R. Gibbs, H. H. H. Goring, R. C. Green, S. Guelfi, N. K. Hansell, C. A. Hartman, K. Hegenscheid, A. Heinz, D.

G. Hernandez, D. J. Heslenfeld, P. J. Hoekstra, F. Holsboer, G. Homuth, J.-J. Hottenga, M. Ikeda, C. R. Jack, Jr., M. Jenkinson, R. Johnson, R. Kanai, M. Keil, J. W. Kent Jr., P. Kochunov, J. B. Kwok, S. M. Lawrie, X. Liu, D. L. Longo, K. L. McMahon, E. Meisenzahl, I. Melle, S. Mohnke, G. W. Montgomery, J. C. Mostert, T. W. Muhleisen, M. A. Nalls, T. E. Nichols, L. G. Nilsson, M. M. Nothen, K. Ohi, R. L. Olvera, R. Perez-Iglesias, G. B. Pike, S. G. Potkin, I. Reinvang, S. Reppermund, M. Rietschel, N. Romanczuk-Seiferth, G. D. Rosen, D. Rujescu, K. Schnell, P. R. Schofield, C. Smith, V. M. Steen, J. E. Sussmann, A. Thalamuthu, A. W. Toga, B. J. Traynor, J. Troncoso, J. A. Turner, M. C. Valdés Hernández, D. van ’t Ent, M. van der Brug, N. J. van der Wee, M. J. van Tol, D. J. Veltman, T. H. Wassink, E. Westman, R. H. Zielke, A. B. Zonderman, D. G. Ashbrook, R. Hager, L. Lu, F. J. McMahon, D. W. Morris, R. W. Williams, H. G. Brunner, R. L. Buckner, J. K. Buitelaar, W. Cahn, V. D. Calhoun, G. L. Cavalleri, B. Crespo-Facorro, A. M. Dale, G. E. Davies, N. Delanty, C. Depondt, S. Djurovic, W. C. Drevets, T. Espeseth, R. L. Gollub, B. C. Ho, W. Hoffmann, N. Hosten, R. S. Kahn, S. Le Hellard, A. Meyer-Lindenberg, B. Muller-Myhsok, M. Nauck, L. Nyberg, M. Pandolfo, B. W. Penninx, J. L. Roffman, S. M. Sisodiya, J. W. Smoller, H. van Bokhoven, N. E. van Haren, H. Volzke, H. Walter, M. W. Weiner, W. Wen, T. White, I. Agartz, O. A. Andreassen, J. Blangero, D. I. Boomsma, R. M. Brouwer, D. M. Cannon, M. R. Cookson, E. J. de Geus, I. J. Deary, G. Donohoe, G. Fernandez, S. E. Fisher, C. Francks, D. C. Glahn, H. J. Grabe, O. Gruber, J. Hardy, R. Hashimoto, H. E. Hulshoff Pol, E. G. Jonsson, I. Kloszewska, S. Lovestone, V. S. Mattay, P. Mecocci, C. McDonald, A. M. McIntosh, R. A. Ophoff, T. Paus, Z. Pausova, M. Ryten, P. S. Sachdev, A. J. Saykin, A. Simmons, A. Singleton, H. Soininen, J. M. Wardlaw, M. E. Weale, D. R. Weinberger, H. H. Adams, L. J. Launer, S. Seiler, R. Schmidt, G. Chauhan, C. L. Satizabal, J. T. Becker, L. Yanek, S. J. van der Lee, M. Ebling, B. Fischl, W. T. Longstreth, Jr., D. Greve, H. Schmidt, P. Nyquist, L. N. Vinke, C. M. van Duijn, L. Xue, B. Mazoyer, J. C. Bis, V. Gudnason, S. Seshadri, M. A. Ikram; Alzheimer’s Disease Neuroimaging Initiative; CHARGE Consortium; EPIGEN; IMAGEN; SYS, N. G. Martin, M. J. Wright, G. Schumann, B. Franke, P. M. Thompson, S. E. Medland, Common genetic variants influence human subcortical brain structures. *Nature* **520**, 224–229 (2015).

38. H. H. Adams, D. P. Hibar, V. Chouraki, J. L. Stein, P. A. Nyquist, M. E. Renteria, S. Trompet, A. Arias-Vasquez, S. Seshadri, S. Desrivieres, A. H. Beecham, N. Jahanshad, K. Wittfeld, S. J. Van der Lee, L. Abramovic, S. Alhusaini, N. Amin, M. Andersson, K. Arfanakis, B. S.

Aribisala, N. J. Armstrong, L. Athanasiu, T. Axelsson, A. Beiser, M. Bernard, J. C. Bis, L. M. Blanken, S. H. Blanton, M. M. Bohlken, M. P. Boks, J. Bralten, A. M. Brickman, O. Carmichael, M. M. Chakravarty, G. Chauhan, Q. Chen, C. R. Ching, G. Cuellar-Partida, A. D. Braber, N. T. Doan, S. Ehrlich, I. Filippi, T. Ge, S. Giddaluru, A. L. Goldman, R. F. Gottesman, C. U. Greven, O. Grimm, M. E. Griswold, T. Guadalupe, J. Hass, U. K. Haukvik, S. Hilal, E. Hofer, D. Hoehn, A. J. Holmes, M. Hoogman, D. Janowitz, T. Jia, D. Kasperaviciute, S. Kim, M. Klein, B. Kraemer, P. H. Lee, J. Liao, D. C. Liewald, L. M. Lopez, M. Luciano, C. Macare, A. Marquand, M. Matarin, K. A. Mather, M. Mattheisen, B. Mazoyer, D. R. McKay, R. McWhirter, Y. Milaneschi, N. Mirza-Schreiber, R. L. Muetzel, S. M. Maniega, K. Nho, A. C. Nugent, L. M. Loohuis, J. Oosterlaan, M. Papmeyer, I. Pappa, L. Pirpamer, S. Pudas, B. Putz, K. B. Rajan, A. Ramasamy, J. S. Richards, S. L. Risacher, R. Roiz-Santianez, N. Rommelse, E. J. Rose, N. A. Royle, T. Rundek, P. G. Samann, C. L. Satizabal, L. Schmaal, A. J. Schork, L. Shen, J. Shin, E. Shumskaya, A. V. Smith, E. Sprooten, L. T. Strike, A. Teumer, R. Thomson, D. Tordesillas-Gutierrez, R. Toro, D. Trabzuni, D. Vaidya, J. Van der Grond, D. Van der Meer, M. M. Van Donkelaar, K. R. Van Eijk, T. G. Van Erp, D. Van Rooij, E. Walton, L. T. Westlye, C. D. Whelan, B. G. Windham, A. M. Winkler, G. Woldehawariat, C. Wolf, T. Wolfers, B. Xu, L. R. Yanek, J. Yang, A. Zijdenbos, M. P. Zwiers, I. Agartz, N. T. Aggarwal, L. Almasy, D. Ames, P. Amouyel, O. A. Andreassen, S. Arepalli, A. A. Assareh, S. Barral, M. E. Bastin, D. M. Becker, J. T. Becker, D. A. Bennett, J. Blangero, H. van Bokhoven, D. I. Boomsma, H. Brodaty, R. M. Brouwer, H. G. Brunner, R. L. Buckner, J. K. Buitelaar, K. B. Bulayeva, W. Cahn, V. D. Calhoun, D. M. Cannon, G. L. Cavalleri, C. Chen, C. Y. Cheng, S. Cichon, M. R. Cookson, A. Corvin, B. Crespo-Facorro, J. E. Curran, M. Czisch, A. M. Dale, G. E. Davies, E. J. De Geus, P. L. De Jager, G. I. de Zubicaray, N. Delanty, C. Depondt, A. L. DeStefano, A. Dillman, S. Djurovic, G. Donohoe, W. C. Drevets, R. Duggirala, T. D. Dyer, S. Erk, T. Espeseth, D. A. Evans, I. O. Fedko, G. Fernandez, L. Ferrucci, S. E. Fisher, D. A. Fleischman, I. Ford, T. M. Foroud, P. T. Fox, C. Francks, M. Fukunaga, J. R. Gibbs, D. C. Glahn, R. L. Gollub, H. H. Goring, H. J. Grabe, R. C. Green, O. Gruber, V. Gudnason, S. Guelfi, N. K. Hansell, J. Hardy, C. A. Hartman, R. Hashimoto, K. Hegenscheid, A. Heinz, S. Le Hellard, D. G. Hernandez, D. J. Heslenfeld, B. C. Ho, P. J. Hoekstra, W. Hoffmann, A. Hofman, F. Holsboer, G. Homuth, N. Hosten, J. J. Hottenga, H. E. Hulshoff Pol, M. Ikeda, M. K. Ikram, C. R. Jack Jr., M. Jenkinson, R. Johnson, E. G. Jonsson, J. W. Jukema, R. S. Kahn, R. Kanai, I. Kloszewska, D.

S. Knopman, P. Kochunov, J. B. Kwok, S. M. Lawrie, H. Lemaitre, X. Liu, D. L. Longo, W. T. Longstreth Jr., O. L. Lopez, S. Lovestone, O. Martinez, J. L. Martinot, V. S. Mattay, C. McDonald, A. M. McIntosh, K. L. McMahon, F. J. McMahon, P. Mecocci, I. Melle, A. Meyer-Lindenberg, S. Mohnke, G. W. Montgomery, D. W. Morris, T. H. Mosley, T. W. Muhleisen, B. Muller-Myhsok, M. A. Nalls, M. Nauck, T. E. Nichols, W. J. Niessen, M. M. Nothen, L. Nyberg, K. Ohi, R. L. Olvera, R. A. Ophoff, M. Pandolfo, T. Paus, Z. Pausova, B. W. Penninx, G. B. Pike, S. G. Potkin, B. M. Psaty, S. Reppermund, M. Rietschel, J. L. Roffman, N. Romanczuk-Seiferth, J. I. Rotter, M. Ryten, R. L. Sacco, P. S. Sachdev, A. J. Saykin, R. Schmidt, P. R. Schofield, S. Sigurdsson, A. Simmons, A. Singleton, S. M. Sisodiya, C. Smith, J. W. Smoller, H. Soininen, V. Srikanth, V. M. Steen, D. J. Stott, J. E. Sussmann, A. Thalamuthu, H. Tiemeier, A. W. Toga, B. J. Traynor, J. Troncoso, J. A. Turner, C. Tzourio, A. G. Uitterlinden, M. C. Hernandez, M. Van der Brug, A. Van der Lugt, N. J. Van der Wee, C. M. Van Duijn, N. E. Van Haren, T. E. D. Van, M. J. Van Tol, B. N. Vardarajan, D. J. Veltman, M. W. Vernooij, H. Volzke, H. Walter, J. M. Wardlaw, T. H. Wassink, M. E. Weale, D. R. Weinberger, M. W. Weiner, W. Wen, E. Westman, T. White, T. Y. Wong, C. B. Wright, H. R. Zielke, A. B. Zonderman, I. J. Deary, C. DeCarli, H. Schmidt, N. G. Martin, A. J. De Craen, M. J. Wright, L. J. Launer, G. Schumann, M. Fornage, B. Franke, S. Debette, S. E. Medland, M. A. Ikram, P. M. Thompson, Novel genetic loci underlying human intracranial volume identified through genome-wide association. *Nat. Neurosci.* **19**, 1569–1582 (2016).

39. M. C. Keller, Gene × environment interaction studies have not properly controlled for potential confounders: The problem and the (simple) solution. *Biol. Psychiatry* **75**, 18–24 (2014).

40. A. D. Skol, L. J. Scott, G. R. Abecasis, M. Boehnke, Joint analysis is more efficient than replication-based analysis for two-stage genome-wide association studies. *Nat. Genet.* **38**, 209–213 (2006).

41. T. K. Lim, E. S. Ruthazer, Microglial trogocytosis and the complement system regulate axonal pruning in vivo. *eLife* **10**, e62167 (2021).

42. V. M. Steen, C. Nepal, K. M. Ersland, R. Holdhus, M. Naevdal, S. M. Ratvik, S. Skrede, B. Havik, Neuropsychological deficits in mice depleted of the schizophrenia susceptibility gene CSMD1. *PLOS ONE* **8**, e79501 (2013).

43. W. Xu, S. Cohen-Woods, Q. Chen, A. Noor, J. Knight, G. Hosang, S. V. Parikh, V. De Luca, F. Tozzi, P. Muglia, J. Forte, A. McQuillin, P. Hu, H. M. Gurling, J. L. Kennedy, P. McGuffin, A. Farmer, J. Strauss, J. B. Vincent, Genome-wide association study of bipolar disorder in Canadian and UK populations corroborates disease loci including SYNE1 and CSMD1. *BMC Med. Genet.* **15**, 2 (2014).

44. M. Patel, Parkinson disease: CSMD1 gene mutations can lead to familial Parkinson disease. *Nat. Rev. Neurol.* **13**, 641 (2017).

45. A. Hatzimanolis, S. Foteli, P. Stefanatou, A. A. Ntigrintaki, I. Ralli, K. Kollias, C. Nikolaou, M. Gazouli, N. C. Stefanis, Deregulation of complement components C4A and CSMD1 peripheral expression in first-episode psychosis and links to cognitive ability. *Eur. Arch. Psychiatry Clin. Neurosci.* **272**, 1219–1228 (2022).

46. L. Athanasiu, S. Giddaluru, C. Fernandes, A. Christoforou, I. Reinvang, A. J. Lundervold, L. G. Nilsson, K. Kauppi, R. Adolfsson, E. Eriksson, K. Sundet, S. Djurovic, T. Espeseth, L. Nyberg, V. M. Steen, O. A. Andreassen, S. Le Hellard, A genetic association study of CSMD1 and CSMD2 with cognitive function. *Brain Behav. Immun.* **61**, 209–216 (2017).

47. K. Abe, O. Chisaka, F. Van Roy, M. Takeichi, Stability of dendritic spines and synaptic contacts is controlled by alpha N-catenin. *Nat. Neurosci.* **7**, 357–363 (2004).

48. C. Park, W. Falls, J. H. Finger, C. M. Longo-Guess, S. L. Ackerman, Deletion in Catna2, encoding alpha N-catenin, causes cerebellar and hippocampal lamination defects and impaired startle modulation. *Nat. Genet.* **31**, 279–284 (2002).

49. A. Smith, I. Bourdeau, J. Wang, C. A. Bondy, Expression of Catenin family members CTNNA1, CTNNA2, CTNNB1 and JUP in the primate prefrontal cortex and hippocampus. *Brain Res. Mol. Brain Res.* **135**, 225–231 (2005).

50. R. Yang, K. K. Walder-Christensen, N. Kim, D. Wu, D. N. Lorenzo, A. Badea, Y. H. Jiang, H. H. Yin, W. C. Wetsel, V. Bennett, ANK2 autism mutation targeting giant ankyrin-B promotes axon branching and ectopic connectivity. *Proc. Natl. Acad. Sci. U.S.A.* **116**, 15262–15271 (2019).

51. M. A. Isungset, D. Conley, H. D. Zachrisson, E. Ystrom, A. Havdahl, P. R. Njolstad, T. H. Lyngstad, Social and genetic associations with educational performance in a Scandinavian welfare state. *Proc. Natl. Acad. Sci. U.S.A.* **119**, e2201869119 (2022).

52. J. J. Lee, R. Wedow, A. Okbay, E. Kong, O. Maghzian, M. Zacher, T. A. Nguyen-Viet, P. Bowers, J. Sidorenko, R. K. Linner, M. A. Fontana, T. Kundu, C. Lee, H. Li, R. Li, R. Royer, P. N. Timshel, R. K. Walters, E. A. Willoughby, L. Yengo; 23andMe Research Team; COGENT (Cognitive Genomics Consortium); Social Science Genetic Association Consortium, M. Alver, Y. Bao, D. W. Clark, F. R. Day, N. A. Furlotte, P. K. Joshi, K. E. Kemper, A. Kleinman, C. Langenberg, R. Magi, J. W. Trampush, S. S. Verma, Y. Wu, M. Lam, J. H. Zhao, Z. Zheng, J. D. Boardman, H. Campbell, J. Freese, K. M. Harris, C. Hayward, P. Herd, M. Kumari, T. Lencz, J. Luan, A. K. Malhotra, A. Metspalu, L. Milani, K. K. Ong, J. R. B. Perry, D. J. Porteous, M. D. Ritchie, M. C. Smart, B. H. Smith, J. Y. Tung, N. J. Wareham, J. F. Wilson, J. P. Beauchamp, D. C. Conley, T. Esko, S. F. Lehrer, P. K. E. Magnusson, S. Oskarsson, T. H. Pers, M. R. Robinson, K. Thom, C. Watson, C. F. Chabris, M. N. Meyer, D. I. Laibson, J. Yang, M. Johannesson, P. D. Koellinger, P. Turley, P. M. Visscher, D. J. Benjamin, D. Cesarini, Gene discovery and polygenic prediction from a genome-wide association study of educational attainment in 1.1 million individuals. *Nat. Genet.* **50**, 1112–1121 (2018).

53. B. Rokholm, K. Silventoinen, P. Tynelius, M. Gamborg, T. I. A. Sorensen, F. Rasmussen, Increasing genetic variance of body mass index during the Swedish obesity epidemic. *PLOS ONE* **6**, e27135 (2011).

54. J. D. Boardman, B. W. Domingue, C. L. Blalock, B. C. Haberstick, K. M. Harris, M. B. McQueen, Is the gene-environment interaction paradigm relevant to genome-wide studies? The case of education and body mass index. *Demography* **51**, 119–139 (2014).

55. A. Citri, R. C. Malenka, Synaptic plasticity: Multiple forms, functions, and mechanisms. *Neuropsychopharmacology* **33**, 18–41 (2008).

56. S. Rumpf, N. Sanal, M. Marzano, Energy metabolic pathways in neuronal development and function. *Oxf. Open Neurosci.* **2**, kvad004 (2023).

57. T. J. Tracey, F. J. Steyn, E. J. Wolvetang, S. T. Ngo, Neuronal lipid metabolism: Multiple pathways driving functional outcomes in health and disease. *Front. Mol. Neurosci.* **11**, 10 (2018).

58. R. P. Bazinet, S. Layé, Polyunsaturated fatty acids and their metabolites in brain function and disease. *Nat. Rev. Neurosci.* **15**, 771–785 (2014).

59. G. W. Kirschen, S. Ge, Editorial: Changes in metabolic processes affecting brain development. *Front. Neurosci.* **16**, 1002010 (2022).

60. Y. Ziv, N. Ron, O. Butovsky, G. Landa, E. Sudai, N. Greenberg, H. Cohen, J. Kipnis, M. Schwartz, Immune cells contribute to the maintenance of neurogenesis and spatial learning abilities in adulthood. *Nat. Neurosci.* **9**, 268–275 (2006).

61. A. Radjavi, I. Smirnov, J. Kipnis, Brain antigen-reactive $CD4^+$ T cells are sufficient to support learning behavior in mice with limited T cell repertoire. *Brain Behav. Immun.* **35**, 58–63 (2014).

62. C. L. Cunningham, V. Martinez-Cerdeno, S. C. Noctor, Microglia regulate the number of neural precursor cells in the developing cerebral cortex. *J. Neurosci.* **33**, 4216–4233 (2013).

63. M. Ueno, Y. Fujita, T. Tanaka, Y. Nakamura, J. Kikuta, M. Ishii, T. Yamashita, Layer V cortical neurons require microglial support for survival during postnatal development. *Nat. Neurosci.* **16**, 543–551 (2013).

64. Y. Pang, L.-W. Fan, L.-T. Tien, X. Dai, B. Zheng, Z. Cai, R. C. S. Lin, A. Bhatt, Differential roles of astrocyte and microglia in supporting oligodendrocyte development and myelination in vitro. *Brain Behav.* **3**, 503–514 (2013).

65. Y. Shigemoto-Mogami, K. Hoshikawa, J. E. Goldman, Y. Sekino, K. Sato, Microglia enhance neurogenesis and oligodendrogenesis in the early postnatal subventricular zone. *J. Neurosci.* **34**, 2231–2243 (2014).

66. B. Stevens, N. J. Allen, L. E. Vazquez, G. R. Howell, K. S. Christopherson, N. Nouri, K. D. Micheva, A. K. Mehalow, A. D. Huberman, B. Stafford, A. Sher, A. M. Litke, J. D. Lambris, S. J. Smith, S. W. John, B. A. Barres, The classical complement cascade mediates CNS synapse elimination. *Cell* **131**, 1164–1178 (2007).

67. A. Miyamoto, H. Wake, A. W. Ishikawa, K. Eto, K. Shibata, H. Murakoshi, S. Koizumi, A. J. Moorhouse, Y. Yoshimura, J. Nabekura, Microglia contact induces synapse formation in developing somatosensory cortex. *Nat. Commun.* **7**, 12540 (2016).

68. R. A. I. Bethlehem, J. Seidlitz, S. R. White, J. W. Vogel, K. M. Anderson, C. Adamson, S. Adler, G. S. Alexopoulos, E. Anagnostou, A. Areces-Gonzalez, D. E. Astle, B. Auyeung, M. Ayub, J. Bae, G. Ball, S. Baron-Cohen, R. Beare, S. A. Bedford, V. Benegal, F. Beyer, J. Blangero, M. B. Cabez, J. P. Boardman, M. Borzage, J. F. Bosch-Bayard, N. Bourke, V. D. Calhoun, M. M. Chakravarty, C. Chen, C. Chertavian, G. Chetelat, Y. S. Chong, J. H. Cole, A. Corvin, M. Costantino, E. Courchesne, F. Crivello, V. L. Cropley, J. Crosbie, N. Crossley, M. Delarue, R. Delorme, S. Desrivieres, G. A. Devenyi, M. A. Di Biase, R. Dolan, K. A. Donald, G. Donohoe, K. Dunlop, A. D. Edwards, J. T. Elison, C. T. Ellis, J. A. Elman, L. Eyler, D. A. Fair, E. Feczko, P. C. Fletcher, P. Fonagy, C. E. Franz, L. Galan-Garcia, A. Gholipour, J. Giedd, J. H. Gilmore, D. C. Glahn, I. M. Goodyer, P. E. Grant, N. A. Groenewold, F. M. Gunning, R. E. Gur, R. C. Gur, C. F. Hammill, O. Hansson, T. Hedden, A. Heinz, R. N. Henson, K. Heuer, J. Hoare, B. Holla, A. J. Holmes, R. Holt, H. Huang, K. Im, J. Ipser, C. R. Jack Jr., A. P. Jackowski, T. Jia, K. A. Johnson, P. B. Jones, D. T. Jones, R. S. Kahn, H. Karlsson, L. Karlsson, R. Kawashima, E. A. Kelley, S. Kern, K. W. Kim, M. G. Kitzbichler, W. S. Kremen, F. Lalonde, B. Landeau, S. Lee, J. Lerch, J. D. Lewis, J. Li, W. Liao, C. Liston, M. V. Lombardo, J. Lv, C. Lynch, T. T. Mallard, M. Marcelis, R. D. Markello, S. R. Mathias, B. Mazoyer, P. McGuire, M. J. Meaney, A. Mechelli, N. Medic, B. Misic, S. E. Morgan, D. Mothersill, J. Nigg, M. Q. W. Ong, C. Ortinau, R. Ossenkoppele, M. Ouyang, L. Palaniyappan, L. Paly, P. M. Pan, C. Pantelis, M. M. Park, T. Paus, Z. Pausova, D. Paz-Linares, A. P. Binette, K. Pierce, X. Qian, J. Qiu, A. Qiu, A. Raznahan, T. Rittman, A.

Rodrigue, C. K. Rollins, R. Romero-Garcia, L. Ronan, M. D. Rosenberg, D. H. Rowitch, G. A. Salum, T. D. Satterthwaite, H. L. Schaare, R. J. Schachar, A. P. Schultz, G. Schumann, M. Scholl, D. Sharp, R. T. Shinohara, I. Skoog, C. D. Smyser, R. A. Sperling, D. J. Stein, A. Stolicyn, J. Suckling, G. Sullivan, Y. Taki, B. Thyreau, R. Toro, N. Traut, K. A. Tsvetanov, N. B. Turk-Browne, J. J. Tuulari, C. Tzourio, E. Vachon-Presseau, M. J. Valdes-Sosa, P. A. Valdes-Sosa, S. L. Valk, T. van Amelsvoort, S. N. Vandekar, L. Vasung, L. W. Victoria, S. Villeneuve, A. Villringer, P. E. Vertes, K. Wagstyl, Y. S. Wang, S. K. Warfield, V. Warrier, E. Westman, M. L. Westwater, H. C. Whalley, A. V. Witte, N. Yang, B. Yeo, H. Yun, A. Zalesky, H. J. Zar, A. Zettergren, J. H. Zhou, H. Ziauddeen, A. Zugman, X. N. Zuo; 3R-BRAIN; AIBL; Alzheimer's Disease Neuroimaging Initiative; Alzheimer's Disease Repository Without Borders Investigators; CALM Team; Cam-CAN; CCNP; COBRE; cVEDA; ENIGMA Developmental Brain Age Working Group; Developing Human Connectome Project; FinnBrain; Harvard Aging Brain Study; IMAGEN; KNE96; Mayo Clinic Study of Aging; NSPN; POND; PREVENT-AD Research Group; VETSA, E. T. Bullmore, A. F. Alexander-Bloch, Brain charts for the human lifespan. *Nature* **604**, 525–533 (2022).

69. W. T. Boyce, M. B. Sokolowski, G. E. Robinson, Genes and environments, development and time. *Proc. Natl. Acad. Sci. U.S.A.* **117**, 23235–23241 (2020).

70. P. N. Alves, C. Foulon, V. Karolis, D. Bzdok, D. S. Margulies, E. Volle, M. Thiebaut de Schotten, An improved neuroanatomical model of the default-mode network reconciles previous neuroimaging and neuropathological findings. *Commun. Biol.* **2**, 370 (2019).

71. W. Li, X. Mai, C. Liu, The default mode network and social understanding of others: What do brain connectivity studies tell us. *Front. Hum. Neurosci.* **8**, 74 (2014).

72. W. Gao, S. Alcauter, A. Elton, C. R. Hernandez-Castillo, J. K. Smith, J. Ramirez, W. Lin, Functional network development during the first year: Relative sequence and socioeconomic correlations. *Cereb. Cortex* **25**, 2919–2928 (2015).

73. S. Whittle, N. Vijayakumar, J. G. Simmons, M. Dennison, O. Schwartz, C. Pantelis, L. Sheeber, M. L. Byrne, N. B. Allen, Role of positive parenting in the association between neighborhood social disadvantage and brain development across adolescence. *JAMA Psychiatry* **74**, 824–832 (2017).

74. F. Degeilh, A. Bernier, E. Leblanc, V. Daneault, M. H. Beauchamp, Quality of maternal behaviour during infancy predicts functional connectivity between default mode network and salience network 9 years later. *Dev. Cogn. Neurosci.* **34**, 53–62 (2018).

75. D. van der Meer, C. A. Hartman, R. H. R. Pruim, M. Mennes, D. Heslenfeld, J. Oosterlaan, S. V. Faraone, B. Franke, J. K. Buitelaar, P. J. Hoekstra, The interaction between 5-HTTLPR and stress exposure influences connectivity of the executive control and default mode brain networks. *Brain Imaging Behav.* **11**, 1486–1496 (2017).

76. S. Scarr-Salapatek, Race, social class, and IQ. *Science* **174**, 1285–1295 (1971).

77. R. Wedow, M. Zacher, B. M. Huibregtse, K. M. Harris, B. W. Domingue, J. D. Boardman, Education, smoking, and cohort change: Forwarding a multidimensional theory of the environmental moderation of genetic effects. *Am. Sociol. Rev.* **83**, 802–832 (2018).

78. H. O. Flemmen, C. S. Simonsen, L. Broch, C. Brunborg, P. Berg-Hansen, S. M. Moen, H. Kersten, E. G. Celius, Maternal education has significant influence on progression in multiple sclerosis. *Mult. Scler. Relat. Disord.* **53**, 103052 (2021).

79. M. A. Rogers, B. L. Plassman, M. Kabeto, G. G. Fisher, J. J. McArdle, D. J. Llewellyn, G. G. Potter, K. M. Langa, Parental education and late-life dementia in the United States. *J. Geriatr. Psychiatry Neurol.* **22**, 71–80 (2009).

80. P. Cermakova, A. Chlapecka, Z. Csajbok, L. Andryskova, M. Brazdil, K. Mareckova, Parental education, cognition and functional connectivity of the salience network. *Sci. Rep.* **13**, 2761 (2023).

81. E. Jarosz, A. Gugushvili, Parental education, health literacy and children's adult body height. *J. Biosoc. Sci.* **52**, 696–718 (2020).

82. P. A. White, Y. A. Awad, L. Gauvin, N. J. Spencer, J. J. McGrath, S. A. Clifford, B. Nikiema, J. Yang-Huang, J. D. Goldhaber-Fiebert, W. Markham, F. K. Mensah, A. van Grieken, H. Raat, V. W. V. Jaddoe, J. Ludvigsson, T. Faresjo; EPOCH Collaborative Group, Household income and maternal education in early childhood and risk of overweight and obesity in late

childhood: Findings from seven birth cohort studies in six high-income countries. *Int. J. Obes.* **46**, 1703–1711 (2022).

83. C. Huang, H. Li, P. W. Liu, J. Zhang, Why does spousal education matter for earnings? Assortative mating and cross-productivity. *J. Labor Econ.* **27**, 633–652 (2009).

84. S. Berlin, Social causes of psychological distress. *Soc. Serv. Rev.* **65**, 331–333 (1991).

85. E. M. Tucker-Drob, T. C. Bates, Large cross-national differences in gene × socioeconomic status interaction on intelligence. *Psychol. Sci.* **27**, 138–149 (2016).

86. D. N. Figlio, J. Freese, K. Karbownik, J. Roth, Socioeconomic status and genetic influences on cognitive development. *Proc. Natl. Acad. Sci. U.S.A.* **114**, 13441–13446 (2017).

87. J. D. Boardman, J. Daw, J. Freese, Defining the environment in gene-environment research: Lessons from social epidemiology. *Am. J. Public Health* **103** Suppl. 1, S64–S72 (2013).

88. N. R. Karcher, D. M. Barch, The ABCD study: Understanding the development of risk for mental and physical health outcomes. *Neuropsychopharmacology* **46**, 131–142 (2021).

89. L. Mascarell Maricic, H. Walter, A. Rosenthal, S. Ripke, E. B. Quinlan, T. Banaschewski, G. J. Barker, A. L. W. Bokde, U. Bromberg, C. Buchel, S. Desrivieres, H. Flor, V. Frouin, H. Garavan, B. Itterman, J. L. Martinot, M. P. Martinot, F. Nees, D. P. Orfanos, T. Paus, L. Poustka, S. Hohmann, M. N. Smolka, J. H. Frohner, R. Whelan, J. Kaminski, G. Schumann, A. Heinz; IMAGEN consortium, The IMAGEN study: A decade of imaging genetics in adolescents. *Mol. Psychiatry* **25**, 2648–2671 (2020).

90. B. J. Casey, T. Cannonier, M. I. Conley, A. O. Cohen, D. M. Barch, M. M. Heitzeg, M. E. Soules, T. Teslovich, D. V. Dellarco, H. Garavan, C. A. Orr, T. D. Wager, M. T. Banich, N. K. Speer, M. T. Sutherland, M. C. Riedel, A. S. Dick, J. M. Bjork, K. M. Thomas, B. Chaarani, M. H. Mejia, D. J. Hagler Jr., M. Daniela Cornejo, C. S. Sicat, M. P. Harms, N. U. F. Dosenbach, M. Rosenberg, E. Earl, H. Bartsch, R. Watts, J. R. Polimeni, J. M. Kuperman, D. A. Fair, A. M. Dale; ABCD Imaging Acquisition Workgroup, The Adolescent Brain

Cognitive Development (ABCD) study: Imaging acquisition across 21 sites. *Dev. Cogn. Neurosci.* **32**, 43–54 (2018).

91. Y. Y. Teo, K. S. Small, D. P. Kwiatkowski, Methodological challenges of genome-wide association analysis in Africa. *Nat. Rev. Genet.* **11**, 149–160 (2010).

92. N. A. Rosenberg, L. Huang, E. M. Jewett, Z. A. Szpiech, I. Jankovic, M. Boehnke, Genome-wide association studies in diverse populations. *Nat. Rev. Genet.* **11**, 356–366 (2010).

93. the Haplotype Reference Consortium, A reference panel of 64,976 haplotypes for genotype imputation. *Nat. Genet.* **48**, 1279–1283 (2016).

94. Q. Luo, Q. Chen, W. Wang, S. Desrivieres, E. B. Quinlan, T. Jia, C. Macare, G. H. Robert, J. Cui, M. Guedj, L. Palaniyappan, F. Kherif, T. Banaschewski, A. L. W. Bokde, C. Buchel, H. Flor, V. Frouin, H. Garavan, P. Gowland, A. Heinz, B. Ittermann, J. L. Martinot, E. Artiges, M. L. Paillere-Martinot, F. Nees, D. P. Orfanos, L. Poustka, J. H. Frohner, M. N. Smolka, H. Walter, R. Whelan, J. H. Callicott, V. S. Mattay, Z. Pausova, J. F. Dartigues, C. Tzourio, F. Crivello, K. F. Berman, F. Li, T. Paus, D. R. Weinberger, R. M. Murray, G. Schumann, J. Feng; IMAGEN consortium, Association of a schizophrenia-risk nonsynonymous variant with putamen volume in adolescents: A voxelwise and genome-wide association study. *JAMA Psychiatry* **76**, 435–445 (2019).

95. The International HapMap 3 Consortium, Integrating common and rare genetic variation in diverse human populations. *Nature* **467**, 52–58 (2010).

96. G. K. Singh, Area deprivation and widening inequalities in US mortality, 1969-1998. *Am. J. Public Health* **93**, 1137–1143 (2003).

97. R. H. Moos, B. S. Moos, *Family Environment Scale Manual: Development, Applications, Research* (Social Climate Scale, Center for Health Care Evaluation, Department of Veterans Affairs, Stanford University Medical Center, ed. 4, 2009).

98. H. C. Karoly, T. Callahan, S. J. Schmiege, S. W. Ewing, Evaluating the hispanic paradox in the context of adolescent risky sexual behavior: The role of parent monitoring. *J. Pediatr. Psychol.* **41**, 429–440 (2016).

99. C. C. Chang, C. C. Chow, L. C. Tellier, S. Vattikuti, S. M. Purcell, J. J. Lee, Second-generation PLINK: Rising to the challenge of larger and richer datasets. *Gigascience* **4**, s13742-015-0047-8 (2015).

100. S. W. Choi, P. F. O'Reilly, PRSice-2: Polygenic risk score software for biobank-scale data. *Gigascience* **8**, giz082 (2019).

101. The 1000 Genomes Project Consortium, A global reference for human genetic variation. *Nature* **526**, 68–74 (2015).

102. M. Ghoussaini, E. Mountjoy, M. Carmona, G. Peat, E. M. Schmidt, A. Hercules, L. Fumis, A. Miranda, D. Carvalho-Silva, A. Buniello, T. Burdett, J. Hayhurst, J. Baker, J. Ferrer, A. Gonzalez-Uriarte, S. Jupp, M. A. Karim, G. Koscielny, S. Machlitt-Northen, C. Malangone, Z. M. Pendlington, P. Roncaglia, D. Suveges, D. Wright, O. Vrousgou, E. Papa, H. Parkinson, J. A. L. MacArthur, J. A. Todd, J. C. Barrett, J. Schwartzentruber, D. G. Hulcoop, D. Ochoa, E. M. McDonagh, I. Dunham, Open targets genetics: Systematic identification of trait-associated genes using large-scale genetics and functional genomics. *Nucleic Acids Res.* **49**, D1311–D1320 (2021).

103. C. A. de Leeuw, J. M. Mooij, T. Heskes, D. Posthuma, MAGMA: Generalized gene-set analysis of GWAS data. *PLOS Comput. Biol.* **11**, e1004219 (2015).

104. K. Watanabe, E. Taskesen, A. van Bochoven, D. Posthuma, Functional mapping and annotation of genetic associations with FUMA. *Nat. Commun.* **8**, 1826 (2017).

105. S. I. Vrieze, W. G. Iacono, M. McGue, Confluence of genes, environment, development, and behavior in a post genome-wide association study world. *Dev. Psychopathol.* **24**, 1195–1214 (2012).

106. J. Li, L. Ji, Adjusting multiple testing in multilocus analyses using the eigenvalues of a correlation matrix. *Heredity* **95**, 221–227 (2005).

107. R. M. Brouwer, M. Klein, K. L. Grasby, H. G. Schnack, N. Jahanshad, J. Teeuw, S. I. Thomopoulos, E. Sprooten, C. E. Franz, N. Gogtay, W. S. Kremen, M. S. Panizzon, L. M. O. Loohuis, C. D. Whelan, M. Aghajani, C. Alloza, D. Alnaes, E. Artiges, R. Ayesa-Arriola, G. J. Barker, M. E. Bastin, E. Blok, E. Boen, I. A. Breukelaar, J. K. Bright, E. E. L. Buimer, R. Bulow, D. M. Cannon, S. Ciufolini, N. A. Crossley, C. G. Damatac, P. Dazzan, C. L. de Mol, S. M. C. de Zwarte, S. Desrivieres, C. M. Diaz-Caneja, N. T. Doan, K. Dohm, J. H. Frohner, J. Goltermann, A. Grigis, D. Grotegerd, L. K. M. Han, M. A. Harris, C. A. Hartman, S. J. Heany, W. Heindel, D. J. Heslenfeld, S. Hohmann, B. Ittermann, P. R. Jansen, J. Janssen, T. Jia, J. Jiang, C. Jockwitz, T. Karali, D. Keeser, M. Koevoets, R. K. Lenroot, B. Malchow, R. C. W. Mandl, V. Medel, S. Meinert, C. A. Morgan, T. W. Muhleisen, L. Nabulsi, N. Opel, V. O. de la Foz, B. J. Overs, M. L. P. Martinot, R. Redlich, T. R. Marques, J. Repple, G. Roberts, G. V. Roshchupkin, N. Setiaman, E. Shumskaya, F. Stein, G. Sudre, S. Takahashi, A. Thalamuthu, D. Tordesillas-Gutierrez, A. van der Lugt, N. E. M. van Haren, J. M. Wardlaw, W. Wen, H. J. Westeneng, K. Wittfeld, A. H. Zhu, A. Zugman, N. J. Armstrong, G. Bonfiglio, J. Bralten, S. Dalvie, G. Davies, M. Di Forti, L. Ding, G. Donohoe, A. J. Forstner, J. Gonzalez-Penas, J. Guimaraes, G. Homuth, J. J. Hottenga, M. J. Knol, J. B. J. Kwok, S. Le Hellard, K. A. Mather, Y. Milaneschi, D. W. Morris, M. M. Nothen, S. Papiol, M. Rietschel, M. L. Santoro, V. M. Steen, J. L. Stein, F. Streit, R. M. Tankard, A. Teumer, D. van Ent, D. van der Meer, K. R. van Eijk, E. Vassos, J. Vazquez-Bourgon, S. H. Witt; the IMAGEN Consortium, H. H. H. Adams, I. Agartz, D. Ames, K. Amunts, O. A. Andreassen, C. Arango, T. Banaschewski, B. T. Baune, S. I. Belangero, A. L. W. Bokde, D. I. Boomsma, R. A. Bressan, H. Brodaty, J. K. Buitelaar, W. Cahn, S. Caspers, S. Cichon, B. Crespo-Facorro, S. R. Cox, U. Dannlowski, T. Elvsashagen, T. Espeseth, P. G. Falkai, S. E. Fisher, H. Flor, J. M. Fullerton, H. Garavan, P. A. Gowland, H. J. Grabe, T. Hahn, A. Heinz, M. Hillegers, J. Hoare, P. J. Hoekstra, M. A. Ikram, A. P. Jackowski, A. Jansen, E. G. Jonsson, R. S. Kahn, T. Kircher, M. S. Korgaonkar, A. Krug, H. Lemaitre, U. F. Malt, J. L. Martinot, C. McDonald, P. B. Mitchell, R. L. Muetzel, R. M. Murray, F. Nees, I. Nenadic, J. Oosterlaan, R. A. Ophoff, P. M. Pan, B. Penninx, L. Poustka, P. S. Sachdev, G. A. Salum, P. R. Schofield, G. Schumann, P. Shaw, K. Sim, M. N. Smolka, D. J. Stein, J. N. Trollor, L. H. van den Berg, J. H. Veldink, H. Walter, L. T. Westlye, R. Whelan, T. White, M.

J. Wright, S. E. Medland, B. Franke, P. M. Thompson, H. E. Hulshoff Pol, Genetic variants associated with longitudinal changes in brain structure across the lifespan. *Nat. Neurosci.* **25**, 421–432 (2022).

108. J. E. Savage, P. R. Jansen, S. Stringer, K. Watanabe, J. Bryois, C. A. de Leeuw, M. Nagel, S. Awasthi, P. B. Barr, J. R. I. Coleman, K. L. Grasby, A. R. Hammerschlag, J. A. Kaminski, R. Karlsson, E. Krapohl, M. Lam, M. Nygaard, C. A. Reynolds, J. W. Trampush, H. Young, D. Zabaneh, S. Hagg, N. K. Hansell, I. K. Karlsson, S. Linnarsson, G. W. Montgomery, A. B. Munoz-Manchado, E. B. Quinlan, G. Schumann, N. G. Skene, B. T. Webb, T. White, D. E. Arking, D. Avramopoulos, R. M. Bilder, P. Bitsios, K. E. Burdick, T. D. Cannon, O. Chiba-Falek, A. Christoforou, E. T. Cirulli, E. Congdon, A. Corvin, G. Davies, I. J. Deary, P. DeRosse, D. Dickinson, S. Djurovic, G. Donohoe, E. D. Conley, J. G. Eriksson, T. Espeseth, N. A. Freimer, S. Giakoumaki, I. Giegling, M. Gill, D. C. Glahn, A. R. Hariri, A. Hatzimanolis, M. C. Keller, E. Knowles, D. Koltai, B. Konte, J. Lahti, S. Le Hellard, T. Lencz, D. C. Liewald, E. London, A. J. Lundervold, A. K. Malhotra, I. Melle, D. Morris, A. C. Need, W. Ollier, A. Palotie, A. Payton, N. Pendleton, R. A. Poldrack, K. Raikkonen, I. Reinvang, P. Roussos, D. Rujescu, F. W. Sabb, M. A. Scult, O. B. Smeland, N. Smyrnis, J. M. Starr, V. M. Steen, N. C. Stefanis, R. E. Straub, K. Sundet, H. Tiemeier, A. N. Voineskos, D. R. Weinberger, E. Widen, J. Yu, G. Abecasis, O. A. Andreassen, G. Breen, L. Christiansen, B. Debrabant, D. M. Dick, A. Heinz, J. Hjerling-Leffler, M. A. Ikram, K. S. Kendler, N. G. Martin, S. E. Medland, N. L. Pedersen, R. Plomin, T. J. C. Polderman, S. Ripke, S. van der Sluis, P. F. Sullivan, S. I. Vrieze, M. J. Wright, D. Posthuma, Genome-wide association meta-analysis in 269,867 individuals identifies new genetic and functional links to intelligence. *Nat. Genet.* **50**, 912–919 (2018).

109. G. Davies, R. E. Marioni, D. C. Liewald, W. D. Hill, S. P. Hagenaars, S. E. Harris, S. J. Ritchie, M. Luciano, C. Fawns-Ritchie, D. Lyall, B. Cullen, S. R. Cox, C. Hayward, D. J. Porteous, J. Evans, A. M. McIntosh, J. Gallacher, N. Craddock, J. P. Pell, D. J. Smith, C. R. Gale, I. J. Deary, Genome-wide association study of cognitive functions and educational attainment in UK Biobank (N=112 151). *Mol. Psychiatry* **21**, 758–767 (2016).

110. S. Sakaue, M. Kanai, Y. Tanigawa, J. Karjalainen, M. Kurki, S. Koshiba, A. Narita, T. Konuma, K. Yamamoto, M. Akiyama, K. Ishigaki, A. Suzuki, K. Suzuki, W. Obara, K.

Yamaji, K. Takahashi, S. Asai, Y. Takahashi, T. Suzuki, N. Shinozaki, H. Yamaguchi, S. Minami, S. Murayama, K. Yoshimori, S. Nagayama, D. Obata, M. Higashiyama, A. Masumoto, Y. Koretsune, F. Gen, K. Ito, C. Terao, T. Yamauchi, I. Komuro, T. Kadowaki, G. Tamiya, M. Yamamoto, Y. Nakamura, M. Kubo, Y. Murakami, K. Yamamoto, Y. Kamatani, A. Palotie, M. A. Rivas, M. J. Daly, K. Matsuda, Y. Okada, A cross-population atlas of genetic associations for 220 human phenotypes. *Nat. Genet.* **53**, 1415–1424 (2021).

111. D. Shungin, T. W. Winkler, D. C. Croteau-Chonka, T. Ferreira, A. E. Locke, R. Magi, R. J. Strawbridge, T. H. Pers, K. Fischer, A. E. Justice, T. Workalemahu, J. M. W. Wu, M. L. Buchkovich, N. L. Heard-Costa, T. S. Roman, A. W. Drong, C. Song, S. Gustafsson, F. R. Day, T. Esko, T. Fall, Z. Kutalik, J. Luan, J. C. Randall, A. Scherag, S. Vedantam, A. R. Wood, J. Chen, R. Fehrmann, J. Karjalainen, B. Kahali, C. T. Liu, E. M. Schmidt, D. Absher, N. Amin, D. Anderson, M. Beekman, J. L. Bragg-Gresham, S. Buyske, A. Demirkan, G. B. Ehret, M. F. Feitosa, A. Goel, A. U. Jackson, T. Johnson, M. E. Kleber, K. Kristiansson, M. Mangino, I. M. Leach, C. Medina-Gomez, C. D. Palmer, D. Pasko, S. Pechlivanis, M. J. Peters, I. Prokopenko, A. Stancakova, Y. J. Sung, T. Tanaka, A. Teumer, J. V. Van Vliet-Ostaptchouk, L. Yengo, W. Zhang, E. Albrecht, J. Arnlov, G. M. Arscott, S. Bandinelli, A. Barrett, C. Bellis, A. J. Bennett, C. Berne, M. Bluher, S. Bohringer, F. Bonnet, Y. Bottcher, M. Bruinenberg, D. B. Carba, I. H. Caspersen, R. Clarke, E. W. Daw, J. Deelen, E. Deelman, G. Delgado, A. S. Doney, N. Eklund, M. R. Erdos, K. Estrada, E. Eury, N. Friedrich, M. E. Garcia, V. Giedraitis, B. Gigante, A. S. Go, A. Golay, H. Grallert, T. B. Grammer, J. Grassler, J. Grewal, C. J. Groves, T. Haller, G. Hallmans, C. A. Hartman, M. Hassinen, C. Hayward, K. Heikkila, K. H. Herzig, Q. Helmer, H. L. Hillege, O. Holmen, S. C. Hunt, A. Isaacs, T. Ittermann, A. L. James, I. Johansson, T. Juliusdottir, I. P. Kalafati, L. Kinnunen, W. Koenig, I. K. Kooner, W. Kratzer, C. Lamina, K. Leander, N. R. Lee, P. Lichtner, L. Lind, J. Lindstrom, S. Lobbens, M. Lorentzon, F. Mach, P. K. Magnusson, A. Mahajan, W. L. McArdle, C. Menni, S. Merger, E. Mihailov, L. Milani, R. Mills, A. Moayyeri, K. L. Monda, S. P. Mooijaart, T. W. Muhleisen, A. Mulas, G. Muller, M. Muller-Nurasyid, R. Nagaraja, M. A. Nalls, N. Narisu, N. Glorioso, I. M. Nolte, M. Olden, N. W. Rayner, F. Renstrom, J. S. Ried, N. R. Robertson, L. M. Rose, S. Sanna, H. Scharnagl, S. Scholtens, B. Sennblad, T. Seufferlein, C. M. Sitlani, A. V. Smith, K. Stirrups, H. M. Stringham, J. Sundstrom, M. A. Swertz, A. J. Swift, A. C. Syvanen, B. O. Tayo, B. Thorand, G. Thorleifsson, A. Tomaschitz, C. Troffa, F. V. van Oort, N. Verweij, J. M. Vonk, L. L. Waite,

R. Wennauer, T. Wilsgaard, M. K. Wojczynski, A. Wong, Q. Zhang, J. H. Zhao, E. P. Brennan, M. Choi, P. Eriksson, L. Folkersen, A. Franco-Cereceda, A. G. Gharavi, A. K. Hedman, M. F. Hivert, J. Huang, S. Kanoni, F. Karpe, S. Keildson, K. Kiryluk, L. Liang, R. P. Lifton, B. Ma, A. J. McKnight, R. McPherson, A. Metspalu, J. L. Min, M. F. Moffatt, G. W. Montgomery, J. M. Murabito, G. Nicholson, D. R. Nyholt, C. Olsson, J. R. Perry, E. Reinmaa, R. M. Salem, N. Sandholm, E. E. Schadt, R. A. Scott, L. Stolk, E. E. Vallejo, H. J. Westra, K. T. Zondervan; The ADIPOGen Consortium; The CARDIOGRAMplusC4D Consortium; The CKDGen Consortium; The GEFOS Consortium; The GENIE Consortium; The GLGC; The ICBP; The International Endogene Consortium; The LifeLines Cohort Study; The MAGIC Investigators; The MuTHER Consortium; The PAGE Consortium; The ReproGen Consortium, P. Amouyel, D. Arveiler, S. J. Bakker, J. Beilby, R. N. Bergman, J. Blangero, M. J. Brown, M. Burnier, H. Campbell, A. Chakravarti, P. S. Chines, S. Claudi-Boehm, F. S. Collins, D. C. Crawford, J. Danesh, U. de Faire, E. J. de Geus, M. Dorr, R. Erbel, J. G. Eriksson, M. Farrall, E. Ferrannini, J. Ferrieres, N. G. Forouhi, T. Forrester, O. H. Franco, R. T. Gansevoort, C. Gieger, V. Gudnason, C. A. Haiman, T. B. Harris, A. T. Hattersley, M. Heliovaara, A. A. Hicks, A. D. Hingorani, W. Hoffmann, A. Hofman, G. Homuth, S. E. Humphries, E. Hypponen, T. Illig, M. R. Jarvelin, B. Johansen, P. Jousilahti, A. M. Jula, J. Kaprio, F. Kee, S. M. Keinanen-Kiukaanniemi, J. S. Kooner, C. Kooperberg, P. Kovacs, A. T. Kraja, M. Kumari, K. Kuulasmaa, J. Kuusisto, T. A. Lakka, C. Langenberg, L. Le Marchand, T. Lehtimaki, V. Lyssenko, S. Mannisto, A. Marette, T. C. Matise, C. A. McKenzie, B. McKnight, A. W. Musk, S. Mohlenkamp, A. D. Morris, M. Nelis, C. Ohlsson, A. J. Oldehinkel, K. K. Ong, L. J. Palmer, B. W. Penninx, A. Peters, P. P. Pramstaller, O. T. Raitakari, T. Rankinen, D. C. Rao, T. K. Rice, P. M. Ridker, M. D. Ritchie, I. Rudan, V. Salomaa, N. J. Samani, J. Saramies, M. A. Sarzynski, P. E. Schwarz, A. R. Shuldiner, J. A. Staessen, V. Steinthorsdottir, R. P. Stolk, K. Strauch, A. Tonjes, A. Tremblay, E. Tremoli, M. C. Vohl, U. Volker, P. Vollenweider, J. F. Wilson, J. C. Witteman, L. S. Adair, M. Bochud, B. O. Boehm, S. R. Bornstein, C. Bouchard, S. Cauchi, M. J. Caulfield, J. C. Chambers, D. I. Chasman, R. S. Cooper, G. Dedoussis, L. Ferrucci, P. Froguel, H. J. Grabe, A. Hamsten, J. Hui, K. Hveem, K. H. Jockel, M. Kivimaki, D. Kuh, M. Laakso, Y. Liu, W. Marz, P. B. Munroe, I. Njolstad, B. A. Oostra, C. N. Palmer, N. L. Pedersen, M. Perola, L. Perusse, U. Peters, C. Power, T. Quertermous, R. Rauramaa, F. Rivadeneira, T. E. Saaristo, D. Saleheen, J. Sinisalo, P. E. Slagboom, H. Snieder, T.

D. Spector, K. Stefansson, M. Stumvoll, J. Tuomilehto, A. G. Uitterlinden, M. Uusitupa, P. van der Harst, G. Veronesi, M. Walker, N. J. Wareham, H. Watkins, H. E. Wichmann, G. R. Abecasis, T. L. Assimes, S. I. Berndt, M. Boehnke, I. B. Borecki, P. Deloukas, L. Franke, T. M. Frayling, L. C. Groop, D. J. Hunter, R. C. Kaplan, J. R. O'Connell, L. Qi, D. Schlessinger, D. P. Strachan, U. Thorsteinsdottir, C. M. van Duijn, C. J. Willer, P. M. Visscher, J. Yang, J. N. Hirschhorn, M. C. Zillikens, M. I. McCarthy, E. K. Speliotes, K. E. North, C. S. Fox, I. Barroso, P. W. Franks, E. Ingelsson, I. M. Heid, R. J. Loos, L. A. Cupples, A. P. Morris, C. M. Lindgren, K. L. Mohlke, New genetic loci link adipose and insulin biology to body fat distribution. *Nature* **518**, 187–196 (2015).

112. L. Jiang, Z. Zheng, H. Fang, J. Yang, A generalized linear mixed model association tool for biobank-scale data. *Nat. Genet.* **53**, 1616–1621 (2021).

113. R. A. Scott, L. J. Scott, R. Magi, L. Marullo, K. J. Gaulton, M. Kaakinen, N. Pervjakova, T. H. Pers, A. D. Johnson, J. D. Eicher, A. U. Jackson, T. Ferreira, Y. Lee, C. Ma, V. Steinthorsdottir, G. Thorleifsson, L. Qi, N. R. Van Zuydam, A. Mahajan, H. Chen, P. Almgren, B. F. Voight, H. Grallert, M. Muller-Nurasyid, J. S. Ried, N. W. Rayner, N. Robertson, L. C. Karssen, E. M. van Leeuwen, S. M. Willems, C. Fuchsberger, P. Kwan, T. M. Teslovich, P. Chanda, M. Li, Y. Lu, C. Dina, D. Thuillier, L. Yengo, L. Jiang, T. Sparso, H. A. Kestler, H. Chheda, L. Eisele, S. Gustafsson, M. Franberg, R. J. Strawbridge, R. Benediktsson, A. B. Hreidarsson, A. Kong, G. Sigurethsson, N. D. Kerrison, J. Luan, L. Liang, T. Meitinger, M. Roden, B. Thorand, T. Esko, E. Mihailov, C. Fox, C. T. Liu, D. Rybin, B. Isomaa, V. Lyssenko, T. Tuomi, D. J. Couper, J. S. Pankow, N. Grarup, C. T. Have, M. E. Jorgensen, T. Jorgensen, A. Linneberg, M. C. Cornelis, R. M. van Dam, D. J. Hunter, P. Kraft, Q. Sun, S. Edkins, K. R. Owen, J. R. B. Perry, A. R. Wood, E. Zeggini, J. Tajes-Fernandes, G. R. Abecasis, L. L. Bonnycastle, P. S. Chines, H. M. Stringham, H. A. Koistinen, L. Kinnunen, B. Sennblad, T. W. Muhleisen, M. M. Nothen, S. Pechlivanis, D. Baldassarre, K. Gertow, S. E. Humphries, E. Tremoli, N. Klopp, J. Meyer, G. Steinbach, R. Wennauer, J. G. Eriksson, S. Männisto, L. Peltonen, E. Tikkanen, G. Charpentier, E. Eury, S. Lobbens, B. Gigante, K. Leander, O. McLeod, E. P. Bottinger, O. Gottesman, D. Ruderfer, M. Bluher, P. Kovacs, A. Tonjes, N. M. Maruthur, C. Scapoli, R. Erbel, K. H. Jockel, S. Moebus, U. de Faire, A. Hamsten, M. Stumvoll, P. Deloukas, P. J. Donnelly, T. M.

Frayling, A. T. Hattersley, S. Ripatti, V. Salomaa, N. L. Pedersen, B. O. Boehm, R. N. Bergman, F. S. Collins, K. L. Mohlke, J. Tuomilehto, T. Hansen, O. Pedersen, I. Barroso, L. Lannfelt, E. Ingelsson, L. Lind, C. M. Lindgren, S. Cauchi, P. Froguel, R. J. F. Loos, B. Balkau, H. Boeing, P. W. Franks, A. B. Gurrea, D. Palli, Y. T. van der Schouw, D. Altshuler, L. C. Groop, C. Langenberg, N. J. Wareham, E. Sijbrands, C. M. van Duijn, J. C. Florez, J. B. Meigs, E. Boerwinkle, C. Gieger, K. Strauch, A. Metspalu, A. D. Morris, C. N. A. Palmer, F. B. Hu, U. Thorsteinsdottir, K. Stefansson, J. Dupuis, A. P. Morris, M. Boehnke, M. I. McCarthy, I. Prokopenko; DIAbetes Genetics Replication And Meta-analysis (DIAGRAM) Consortium, An expanded genome-wide association study of type 2 diabetes in europeans. *Diabetes* **66**, 2888–2902 (2017).

114. K. P. Glanville, J. R. I. Coleman, P. F. O'Reilly, J. Galloway, C. M. Lewis, Investigating pleiotropy between depression and autoimmune diseases using the UK biobank. *Biol. Psychiatry Glob. Open Sci.* **1**, 48–58 (2021).

115. Y. Okada, D. Wu, G. Trynka, T. Raj, C. Terao, K. Ikari, Y. Kochi, K. Ohmura, A. Suzuki, S. Yoshida, R. R. Graham, A. Manoharan, W. Ortmann, T. Bhangale, J. C. Denny, R. J. Carroll, A. E. Eyler, J. D. Greenberg, J. M. Kremer, D. A. Pappas, L. Jiang, J. Yin, L. Ye, D. F. Su, J. Yang, G. Xie, E. Keystone, H. J. Westra, T. Esko, A. Metspalu, X. Zhou, N. Gupta, D. Mirel, E. A. Stahl, D. Diogo, J. Cui, K. Liao, M. H. Guo, K. Myouzen, T. Kawaguchi, M. J. Coenen, P. L. van Riel, M. A. van de Laar, H. J. Guchelaar, T. W. Huizinga, P. Dieude, X. Mariette, S. L. Bridges Jr., A. Zhernakova, R. E. Toes, P. P. Tak, C. Miceli-Richard, S. Y. Bang, H. S. Lee, J. Martin, M. A. Gonzalez-Gay, L. Rodriguez-Rodriguez, S. Rantapaa-Dahlqvist, L. Arlestig, H. K. Choi, Y. Kamatani, P. Galan, M. Lathrop; the RACI consortium; the GARNET consortium, S. Eyre, J. Bowes, A. Barton, N. de Vries, L. W. Moreland, L. A. Criswell, E. W. Karlson, A. Taniguchi, R. Yamada, M. Kubo, J. S. Liu, S. C. Bae, J. Worthington, L. Padyukov, L. Klareskog, P. K. Gregersen, S. Raychaudhuri, B. E. Stranger, P. L. De Jager, L. Franke, P. M. Visscher, M. A. Brown, H. Yamanaka, T. Mimori, A. Takahashi, H. Xu, T. W. Behrens, K. A. Siminovitch, S. Momohara, F. Matsuda, K. Yamamoto, R. M. Plenge, Genetics of rheumatoid arthritis contributes to biology and drug discovery. *Nature* **506**, 376–381 (2014).

116. I. Pappa, B. S. Pourcain, K. Benke, A. Cavadino, C. Hakulinen, M. G. Nivard, I. M. Nolte, C. M. Tiesler, M. J. Bakermans-Kranenburg, G. E. Davies, D. M. Evans, M. C. Geoffroy, H. Grallert, M. M. Groen-Blokhuis, J. J. Hudziak, J. P. Kemp, L. Keltikangas-Jarvinen, G. McMahon, V. R. Mileva-Seitz, E. Motazedi, C. Power, O. T. Raitakari, S. M. Ring, F. Rivadeneira, A. Rodriguez, P. A. Scheet, I. Seppala, H. Snieder, M. Standl, E. Thiering, N. J. Timpson, R. Veenstra, F. P. Velders, A. J. Whitehouse, G. D. Smith, J. Heinrich, E. Hypponen, T. Lehtimaki, C. M. Middeldorp, A. J. Oldehinkel, C. E. Pennell, D. I. Boomsma, H. Tiemeier, A genome-wide approach to children's aggressive behavior: The EAGLE consortium. *Am. J. Med. Genet. B Neuropsychiatr. Genet.* **171**, 562–572 (2016).

117. D. Demontis, R. K. Walters, J. Martin, M. Mattheisen, T. D. Als, E. Agerbo, G. Baldursson, R. Belliveau, J. Bybjerg-Grauholm, M. Baekvad-Hansen, F. Cerrato, K. Chambert, C. Churchhouse, A. Dumont, N. Eriksson, M. Gandal, J. I. Goldstein, K. L. Grasby, J. Grove, O. O. Gudmundsson, C. S. Hansen, M. E. Hauberg, M. V. Hollegaard, D. P. Howrigan, H. Huang, J. B. Maller, A. R. Martin, N. G. Martin, J. Moran, J. Pallesen, D. S. Palmer, C. B. Pedersen, M. G. Pedersen, T. Poterba, J. B. Poulsen, S. Ripke, E. B. Robinson, F. K. Satterstrom, H. Stefansson, C. Stevens, P. Turley, G. B. Walters, H. Won, M. J. Wright; ADHD Working Group of the Psychiatric Genomics Consortium (PGC); Early Lifecourse & Genetic Epidemiology (EAGLE) Consortium; 23andMe Research Team, O. A. Andreassen, P. Asherson, C. L. Burton, D. I. Boomsma, B. Cormand, S. Dalsgaard, B. Franke, J. Gelernter, D. Geschwind, H. Hakonarson, J. Haavik, H. R. Kranzler, J. Kuntsi, K. Langley, K. P. Lesch, C. Middeldorp, A. Reif, L. A. Rohde, P. Roussos, R. Schachar, P. Sklar, E. J. S. Sonuga-Barke, P. F. Sullivan, A. Thapar, J. Y. Tung, I. D. Waldman, S. E. Medland, K. Stefansson, M. Nordentoft, D. M. Hougaard, T. Werge, O. Mors, P. B. Mortensen, M. J. Daly, S. V. Faraone, A. D. Borglum, B. M. Neale, Discovery of the first genome-wide significant risk loci for attention deficit/hyperactivity disorder. *Nat. Genet.* **51**, 63–75 (2019).

118. J. Grove, S. Ripke, T. D. Als, M. Mattheisen, R. K. Walters, H. Won, J. Pallesen, E. Agerbo, O. A. Andreassen, R. Anney, S. Awashti, R. Belliveau, F. Bettella, J. D. Buxbaum, J. Bybjerg-Grauholm, M. Baekvad-Hansen, F. Cerrato, K. Chambert, J. H. Christensen, C. Churchhouse, K. Dellenvall, D. Demontis, S. De Rubeis, B. Devlin, S. Djurovic, A. L. Dumont, J. I. Goldstein, C. S. Hansen, M. E. Hauberg, M. V. Hollegaard, S. Hope, D. P.

Howrigan, H. Huang, C. M. Hultman, L. Klei, J. Maller, J. Martin, A. R. Martin, J. L. Moran, M. Nyegaard, T. Naerland, D. S. Palmer, A. Palotie, C. B. Pedersen, M. G. Pedersen, T. dPoterba, J. B. Poulsen, B. S. Pourcain, P. Qvist, K. Rehnstrom, A. Reichenberg, J. Reichert, E. B. Robinson, K. Roeder, P. Roussos, E. Saemundsen, S. Sandin, F. K. Satterstrom, G. D. Smith, H. Stefansson, S. Steinberg, C. R. Stevens, P. F. Sullivan, P. Turley, G. B. Walters, X. Xu; Autism Spectrum Disorder Working Group of the Psychiatric Genomics Consortium; BUPGEN; Major Depressive Disorder Working Group of the Psychiatric Genomics Consortium; 23andMe Research Team, K. Stefansson, D. H. Geschwind, M. Nordentoft, D. M. Hougaard, T. Werge, O. Mors, P. B. Mortensen, B. M. Neale, M. J. Daly, A. D. Borglum, Identification of common genetic risk variants for autism spectrum disorder. *Nat. Genet.* **51**, 431–444 (2019).

119. D. M. Howard, M. J. Adams, T. K. Clarke, J. D. Hafferty, J. Gibson, M. Shirali, J. R. I. Coleman, S. P. Hagenaars, J. Ward, E. M. Wigmore, C. Alloza, X. Shen, M. C. Barbu, E. Y. Xu, H. C. Whalley, R. E. Marioni, D. J. Porteous, G. Davies, I. J. Deary, G. Hemani, K. Berger, H. Teismann, R. Rawal, V. Arolt, B. T. Baune, U. Dannlowski, K. Domschke, C. Tian, D. A. Hinds; 23andMe Research Team; Major Depressive Disorder Working Group of the Psychiatric Genomics Consortium, M. Trzaskowski, E. M. Byrne, S. Ripke, D. J. Smith, P. F. Sullivan, N. R. Wray, G. Breen, C. M. Lewis, A. M. McIntosh, Genome-wide meta-analysis of depression identifies 102 independent variants and highlights the importance of the prefrontal brain regions. *Nat. Neurosci.* **22**, 343–352 (2019).

120. V. Trubetskoy, A. F. Pardinas, T. Qi, G. Panagiotaropoulou, S. Awasthi, T. B. Bigdeli, J. Bryois, C. Y. Chen, C. A. Dennison, L. S. Hall, M. Lam, K. Watanabe, O. Frei, T. Ge, J. C. Harwood, F. Koopmans, S. Magnusson, A. L. Richards, J. Sidorenko, Y. Wu, J. Zeng, J. Grove, M. Kim, Z. Li, G. Voloudakis, W. Zhang, M. Adams, I. Agartz, E. G. Atkinson, E. Agerbo, M. Al Eissa, M. Albus, M. Alexander, B. Z. Alizadeh, K. Alptekin, T. D. Als, F. Amin, V. Arolt, M. Arrojo, L. Athanasiu, M. H. Azevedo, S. A. Bacanu, N. J. Bass, M. Begemann, R. A. Belliveau, J. Bene, B. Benyamin, S. E. Bergen, G. Blasi, J. Bobes, S. Bonassi, A. Braun, R. A. Bressan, E. J. Bromet, R. Bruggeman, P. F. Buckley, R. L. Buckner, J. Bybjerg-Grauholm, W. Cahn, M. J. Cairns, M. E. Calkins, V. J. Carr, D. Castle, S. V. Catts, K. D. Chambert, R. C. K. Chan, B. Chaumette, W. Cheng, E. F. C. Cheung, S. A. Chong, D. Cohen, A. Consoli, Q. Cordeiro, J. Costas, C. Curtis, M. Davidson, K. L. Davis,

L. de Haan, F. Degenhardt, L. E. DeLisi, D. Demontis, F. Dickerson, D. Dikeos, T. Dinan, S. Djurovic, J. Duan, G. Ducci, F. Dudbridge, J. G. Eriksson, L. Fananas, S. V. Faraone, A. Fiorentino, A. Forstner, J. Frank, N. B. Freimer, M. Fromer, A. Frustaci, A. Gadelha, G. Genovese, E. S. Gershon, M. Giannitelli, I. Giegling, P. Giusti-Rodriguez, S. Godard, J. I. Goldstein, J. G. Penas, A. Gonzalez-Pinto, S. Gopal, J. Gratten, M. F. Green, T. A. Greenwood, O. Guillin, S. Guloksuz, R. E. Gur, R. C. Gur, B. Gutierrez, E. Hahn, H. Hakonarson, V. Haroutunian, A. M. Hartmann, C. Harvey, C. Hayward, F. A. Henskens, S. Herms, P. Hoffmann, D. P. Howrigan, M. Ikeda, C. Iyegbe, I. Joa, A. Julia, A. K. Kahler, T. Kam-Thong, Y. Kamatani, S. Karachanak-Yankova, O. Kebir, M. C. Keller, B. J. Kelly, A. Khrunin, S. W. Kim, J. Klovins, N. Kondratiev, B. Konte, J. Kraft, M. Kubo, V. Kucinskas, Z. A. Kucinskiene, A. Kusumawardhani, H. Kuzelova-Ptackova, S. Landi, L. C. Lazzeroni, P. H. Lee, S. E. Legge, D. S. Lehrer, R. Lencer, B. Lerer, M. Li, J. Lieberman, G. A. Light, S. Limborska, C. M. Liu, J. Lonnqvist, C. M. Loughland, J. Lubinski, J. J. Luykx, A. Lynham, M. Macek Jr., A. Mackinnon, P. K. E. Magnusson, B. S. Maher, W. Maier, D. Malaspina, J. Mallet, S. R. Marder, S. Marsal, A. R. Martin, L. Martorell, M. Mattheisen, R. W. McCarley, C. McDonald, J. J. McGrath, H. Medeiros, S. Meier, B. Melegh, I. Melle, R. I. Mesholam-Gately, A. Metspalu, P. T. Michie, L. Milani, V. Milanova, M. Mitjans, E. Molden, E. Molina, M. D. Molto, V. Mondelli, C. Moreno, C. P. Morley, G. Muntane, K. C. Murphy, I. Myin-Germeys, I. Nenadic, G. Nestadt, L. Nikitina-Zake, C. Noto, K. H. Nuechterlein, N. L. O'Brien, F. A. O'Neill, S. Y. Oh, A. Olincy, V. K. Ota, C. Pantelis, G. N. Papadimitriou, M. Parellada, T. Paunio, R. Pellegrino, S. Periyasamy, D. O. Perkins, B. Pfuhlmann, O. Pietilainen, J. Pimm, D. Porteous, J. Powell, D. Quattrone, D. Quested, A. D. Radant, A. Rampino, M. H. Rapaport, A. Rautanen, A. Reichenberg, C. Roe, J. L. Roffman, J. Roth, M. Rothermundt, B. P. F. Rutten, S. Saker-Delye, V. Salomaa, J. Sanjuan, M. L. Santoro, A. Savitz, U. Schall, R. J. Scott, L. J. Seidman, S. I. Sharp, J. Shi, L. J. Siever, E. Sigurdsson, K. Sim, N. Skarabis, P. Slominsky, H. C. So, J. L. Sobell, E. Soderman, H. J. Stain, N. E. Steen, A. A. Steixner-Kumar, E. Stogmann, W. S. Stone, R. E. Straub, F. Streit, E. Strengman, T. S. Stroup, M. Subramaniam, C. A. Sugar, J. Suvisaari, D. M. Svrakic, N. R. Swerdlow, J. P. Szatkiewicz, T. M. T. Ta, A. Takahashi, C. Terao, F. Thibaut, D. Toncheva, P. A. Tooney, S. Torretta, S. Tosato, G. B. Tura, B. I. Turetsky, A. Ucok, A. Vaaler, T. van Amelsvoort, R. van Winkel, J. Veijola, J. Waddington, H. Walter, A. Waterreus, B. T. Webb, M. Weiser, N. M. Williams, S. H. Witt, B. K. Wormley, J. Q. Wu, Z. Xu, R. Yolken, C. C. Zai,

W. Zhou, F. Zhu, F. Zimprich, E. C. Atbasoglu, M. Ayub, C. Benner, A. Bertolino, D. W. Black, N. J. Bray, G. Breen, N. G. Buccola, W. F. Byerley, W. J. Chen, C. R. Cloninger, B. Crespo-Facorro, G. Donohoe, R. Freedman, C. Galletly, M. J. Gandal, M. Gennarelli, D. M. Hougaard, H. G. Hwu, A. V. Jablensky, S. A. McCarroll, J. L. Moran, O. Mors, P. B. Mortensen, B. Muller-Myhsok, A. L. Neil, M. Nordentoft, M. T. Pato, T. L. Petryshen, M. Pirinen, A. E. Pulver, T. G. Schulze, J. M. Silverman, J. W. Smoller, E. A. Stahl, D. W. Tsuang, E. Vilella, S. H. Wang, S. Xu; Indonesia Schizophrenia Consortium; PsychENCODE; Psychosis Endophenotypes International Consortium; SynGO Consortium, R. Adolfsson, C. Arango, B. T. Baune, S. I. Belangero, A. D. Borglum, D. Braff, E. Bramon, J. D. Buxbaum, D. Campion, J. A. Cervilla, S. Cichon, D. A. Collier, A. Corvin, D. Curtis, M. D. Forti, E. Domenici, H. Ehrenreich, V. Escott-Price, T. Esko, A. H. Fanous, A. Gareeva, M. Gawlik, P. V. Gejman, M. Gill, S. J. Glatt, V. Golimbet, K. S. Hong, C. M. Hultman, S. E. Hyman, N. Iwata, E. G. Jonsson, R. S. Kahn, J. L. Kennedy, E. Khusnutdinova, G. Kirov, J. A. Knowles, M. O. Krebs, C. Laurent-Levinson, J. Lee, T. Lencz, D. F. Levinson, Q. S. Li, J. Liu, A. K. Malhotra, D. Malhotra, A. McIntosh, A. McQuillin, P. R. Menezes, V. A. Morgan, D. W. Morris, B. J. Mowry, R. M. Murray, V. Nimgaonkar, M. M. Nothen, R. A. Ophoff, S. A. Paciga, A. Palotie, C. N. Pato, S. Qin, M. Rietschel, B. P. Riley, M. Rivera, D. Rujescu, M. C. Saka, A. R. Sanders, S. G. Schwab, A. Serretti, P. C. Sham, Y. Shi, D. S. Clair, H. Stefansson, K. Stefansson, M. T. Tsuang, J. van Os, M. P. Vawter, D. R. Weinberger, T. Werge, D. B. Wildenauer, X. Yu, W. Yue, P. A. Holmans, A. J. Pocklington, P. Roussos, E. Vassos, M. Verhage, P. M. Visscher, J. Yang, D. Posthuma, O. A. Andreassen, K. S. Kendler, M. J. Owen, N. R. Wray, M. J. Daly, H. Huang, B. M. Neale, P. F. Sullivan, S. Ripke, J. T. R. Walters, M. C. O'Donovan; Schizophrenia Working Group of the Psychiatric Genomics Consortium, Mapping genomic loci implicates genes and synaptic biology in schizophrenia. *Nature* **604**, 502–508 (2022).

121. E. A. Stahl, G. Breen, A. J. Forstner, A. McQuillin, S. Ripke, V. Trubetskoy, M. Mattheisen, Y. Wang, J. R. I. Coleman, H. A. Gaspar, C. A. de Leeuw, S. Steinberg, J. M. W. Pavlides, M. Trzaskowski, E. M. Byrne, T. H. Pers, P. A. Holmans, A. L. Richards, L. Abbott, E. Agerbo, H. Akil, D. Albani, N. Alliey-Rodriguez, T. D. Als, A. Anjorin, V. Antilla, S. Awasthi, J. A. Badner, M. Baekvad-Hansen, J. D. Barchas, N. Bass, M. Bauer, R. Belliveau, S. E. Bergen, C. B. Pedersen, E. Boen, M. P. Boks, J. Boocock, M. Budde, W. Bunney, M. Burmeister, J. Bybjerg-Grauholm, W. Byerley, M. Casas, F. Cerrato, P. Cervantes, K.

Chambert, A. W. Charney, D. Chen, C. Churchhouse, T. K. Clarke, W. Coryell, D. W. Craig, C. Cruceanu, D. Curtis, P. M. Czerski, A. M. Dale, S. de Jong, F. Degenhardt, J. Del-Favero, J. R. DePaulo, S. Djurovic, A. L. Dobbyn, A. Dumont, T. Elvsashagen, V. Escott-Price, C. C. Fan, S. B. Fischer, M. Flickinger, T. M. Foroud, L. Forty, J. Frank, C. Fraser, N. B. Freimer, L. Frisen, K. Gade, D. Gage, J. Garnham, C. Giambartolomei, M. G. Pedersen, J. Goldstein, S. D. Gordon, K. Gordon-Smith, E. K. Green, M. J. Green, T. A. Greenwood, J. Grove, W. Guan, J. Guzman-Parra, M. L. Hamshere, M. Hautzinger, U. Heilbronner, S. Herms, M. Hipolito, P. Hoffmann, D. Holland, L. Huckins, S. Jamain, J. S. Johnson, A. Jureus, R. Kandaswamy, R. Karlsson, J. L. Kennedy, S. Kittel-Schneider, J. A. Knowles, M. Kogevinas, A. C. Koller, R. Kupka, C. Lavebratt, J. Lawrence, W. B. Lawson, M. Leber, P. H. Lee, S. E. Levy, J. Z. Li, C. Liu, S. Lucae, A. Maaser, D. J. MacIntyre, P. B. Mahon, W. Maier, L. Martinsson, S. McCarroll, P. McGuffin, M. G. McInnis, J. D. McKay, H. Medeiros, S. E. Medland, F. Meng, L. Milani, G. W. Montgomery, D. W. Morris, T. W. Muhleisen, N. Mullins, H. Nguyen, C. M. Nievergelt, A. N. Adolfsson, E. A. Nwulia, C. O'Donovan, L. M. O. Loohuis, A. P. S. Ori, L. Oruc, U. Osby, R. H. Perlis, A. Perry, A. Pfennig, J. B. Potash, S. M. Purcell, E. J. Regeer, A. Reif, C. S. Reinbold, J. P. Rice, F. Rivas, M. Rivera, P. Roussos, D. M. Ruderfer, E. Ryu, C. Sanchez-Mora, A. F. Schatzberg, W. A. Scheftner, N. J. Schork, C. S. Weickert, T. Shehktman, P. D. Shilling, E. Sigurdsson, C. Slaney, O. B. Smeland, J. L. Sobell, C. S. Hansen, A. T. Spijker, D. S. Clair, M. Steffens, J. S. Strauss, F. Streit, J. Strohmaier, S. Szelinger, R. C. Thompson, T. E. Thorgeirsson, J. Treutlein, H. Vedder, W. Wang, S. J. Watson, T. W. Weickert, S. H. Witt, S. Xi, W. Xu, A. H. Young, P. Zandi, P. Zhang, S. Zollner; eQTLGen Consortium; BIOS Consortium, R. Adolfsson, I. Agartz, M. Alda, L. Backlund, B. T. Baune, F. Bellivier, W. H. Berrettini, J. M. Biernacka, D. H. R. Blackwood, M. Boehnke, A. D. Borglum, A. Corvin, N. Craddock, M. J. Daly, U. Dannlowski, T. Esko, B. Etain, M. Frye, J. M. Fullerton, E. S. Gershon, M. Gill, F. Goes, M. Grigoroiu-Serbanescu, J. Hauser, D. M. Hougaard, C. M. Hultman, I. Jones, L. A. Jones, R. S. Kahn, G. Kirov, M. Landen, M. Leboyer, C. M. Lewis, Q. S. Li, J. Lissowska, N. G. Martin, F. Mayoral, S. L. McElroy, A. M. McIntosh, F. J. McMahon, I. Melle, A. Metspalu, P. B. Mitchell, G. Morken, O. Mors, P. B. Mortensen, B. Muller-Myhsok, R. M. Myers, B. M. Neale, V. Nimgaonkar, M. Nordentoft, M. M. Nothen, M. C. O'Donovan, K. J. Oedegaard, M. J. Owen, S. A. Paciga, C. Pato, M. T. Pato, D. Posthuma, J. A. Ramos-Quiroga, M. Ribases, M. Rietschel, G. A. Rouleau, M. Schalling, P. R. Schofield, T. G.

Schulze, A. Serretti, J. W. Smoller, H. Stefansson, K. Stefansson, E. Stordal, P. F. Sullivan, G. Turecki, A. E. Vaaler, E. Vieta, J. B. Vincent, T. Werge, J. I. Nurnberger, N. R. Wray, A. Di Florio, H. J. Edenberg, S. Cichon, R. A. Ophoff, L. J. Scott, O. A. Andreassen, J. Kelsoe, P. Sklar; the Bipolar Disorder Working Group of the Psychiatric Genomics Consortium, Genome-wide association study identifies 30 loci associated with bipolar disorder. *Nat. Genet.* **51**, 793–803 (2019).

122. C. Bellenguez, F. Kucukali, I. E. Jansen, L. Kleineidam, S. Moreno-Grau, N. Amin, A. C. Naj, R. Campos-Martin, B. Grenier-Boley, V. Andrade, P. A. Holmans, A. Boland, V. Damotte, S. J. van der Lee, M. R. Costa, T. Kuulasmaa, Q. Yang, I. de Rojas, J. C. Bis, A. Yaqub, I. Prokic, J. Chapuis, S. Ahmad, V. Giedraitis, D. Aarsland, P. Garcia-Gonzalez, C. Abdelnour, E. Alarcon-Martin, D. Alcolea, M. Alegret, I. Alvarez, V. Alvarez, N. J. Armstrong, A. Tsolaki, C. Antunez, I. Appollonio, M. Arcaro, S. Archetti, A. A. Pastor, B. Arosio, L. Athanasiu, H. Bailly, N. Banaj, M. Baquero, S. Barral, A. Beiser, A. B. Pastor, J. E. Below, P. Benchek, L. Benussi, C. Berr, C. Besse, V. Bessi, G. Binetti, A. Bizarro, R. Blesa, M. Boada, E. Boerwinkle, B. Borroni, S. Boschi, P. Bossu, G. Brathen, J. Bressler, C. Bresner, H. Brodaty, K. J. Brookes, L. I. Brusco, D. Buiza-Rueda, K. Burger, V. Burholt, W. S. Bush, M. Calero, L. B. Cantwell, G. Chene, J. Chung, M. L. Cuccaro, A. Carracedo, R. Cecchetti, L. Cervera-Carles, C. Charbonnier, H. H. Chen, C. Chillotti, S. Ciccone, J. Claassen, C. Clark, E. Conti, A. Corma-Gomez, E. Costantini, C. Custodero, D. Daian, M. C. Dalmasso, A. Daniele, E. Dardiotis, J. F. Dartigues, P. P. de Deyn, K. de Paiva Lopes, L. D. de Witte, S. Debette, J. Deckert, T. Del Ser, N. Denning, A. DeStefano, M. Dichgans, J. Diehl-Schmid, M. Diez-Fairen, P. D. Rossi, S. Djurovic, E. Duron, E. Duzel, C. Dufouil, G. Eiriksdottir, S. Engelborghs, V. Escott-Price, A. Espinosa, M. Ewers, K. M. Faber, T. Fabrizio, S. F. Nielsen, D. W. Fardo, L. Farotti, C. Fenoglio, M. Fernandez-Fuertes, R. Ferrari, C. B. Ferreira, E. Ferri, B. Fin, P. Fischer, T. Fladby, K. Fliessbach, B. Fongang, M. Fornage, J. Fortea, T. M. Foroud, S. Fostinelli, N. C. Fox, E. Franco-Macias, M. J. Bullido, A. Frank-Garcia, L. Froelich, B. Fulton-Howard, D. Galimberti, J. M. Garcia-Alberca, P. Garcia-Gonzalez, S. Garcia-Madrona, G. Garcia-Ribas, R. Ghidoni, I. Giegling, G. Giorgio, A. M. Goate, O. Goldhardt, D. Gomez-Fonseca, A. Gonzalez-Perez, C. Graff, G. Grande, E. Green, T. Grimmer, E. Grunblatt, M. Grunin, V. Gudnason, T. Guetta-Baranes, A. Haapasalo, G. Hadjigeorgiou, J. L. Haines, K. L. Hamilton-Nelson, H. Hampel, O. Hanon, J. Hardy, A. M. Hartmann, L. Hausner, J. Harwood, S. Heilmann-Heimbach, S. Helisalmi,

M. T. Heneka, I. Hernandez, M. J. Herrmann, P. Hoffmann, C. Holmes, H. Holstege, R. H. Vilas, M. Hulsman, J. Humphrey, G. J. Biessels, X. Jian, C. Johansson, G. R. Jun, Y. Kastumata, J. Kauwe, P. G. Kehoe, L. Kilander, A. K. Stahlbom, M. Kivipelto, A. Koivisto, J. Kornhuber, M. H. Kosmidis, W. A. Kukull, P. P. Kuksa, B. W. Kunkle, A. B. Kuzma, C. Lage, E. J. Laukka, L. Launer, A. Lauria, C. Y. Lee, J. Lehtisalo, O. Lerch, A. Lleo, W. Longstreth Jr., O. Lopez, A. L. de Munain, S. Love, M. Lowemark, L. Luckcuck, K. L. Lunetta, Y. Ma, J. Macias, C. A. MacLeod, W. Maier, F. Mangialasche, M. Spallazzi, M. Marquie, R. Marshall, E. R. Martin, A. M. Montes, C. M. Rodriguez, C. Masullo, R. Mayeux, S. Mead, P. Mecocci, M. Medina, A. Meggy, S. Mehrabian, S. Mendoza, M. Menendez-Gonzalez, P. Mir, S. Moebus, M. Mol, L. Molina-Porcel, L. Montrreal, L. Morelli, F. Moreno, K. Morgan, T. Mosley, M. M. Nothen, C. Muchnik, S. Mukherjee, B. Nacmias, T. Ngandu, G. Nicolas, B. G. Nordestgaard, R. Olaso, A. Orellana, M. Orsini, G. Ortega, A. Padovani, C. Paolo, G. Papenberg, L. Parnetti, F. Pasquier, P. Pastor, G. Peloso, A. Perez-Cordon, J. Perez-Tur, P. Pericard, O. Peters, Y. A. L. Pijnenburg, J. A. Pineda, G. Pinol-Ripoll, C. Pisanu, T. Polak, J. Popp, D. Posthuma, J. Priller, R. Puerta, O. Quenez, I. Quintela, J. Q. Thomassen, A. Rabano, I. Rainero, F. Rajabli, I. Ramakers, L. M. Real, M. J. T. Reinders, C. Reitz, D. Reyes-Dumeyer, P. Ridge, S. Riedel-Heller, P. Riederer, N. Roberto, E. Rodriguez-Rodriguez, A. Rongve, I. R. Allende, M. Rosende-Roca, J. L. Royo, E. Rubino, D. Rujescu, M. E. Saez, P. Sakka, I. Saltvedt, A. Sanabria, M. B. Sanchez-Arjona, F. Sanchez-Garcia, P. S. Juan, R. Sanchez-Valle, S. B. Sando, C. Sarnowski, C. L. Satizabal, M. Scamosci, N. Scarmeas, E. Scarpini, P. Scheltens, N. Scherbaum, M. Scherer, M. Schmid, A. Schneider, J. M. Schott, G. Selbaek, D. Seripa, M. Serrano, J. Sha, A. A. Shadrin, O. Skrobot, S. Slifer, G. J. L. Snijders, H. Soininen, V. Solfrizzi, A. Solomon, Y. Song, S. Sorbi, O. Sotolongo-Grau, G. Spalletta, A. Spottke, A. Squassina, E. Stordal, J. P. Tartan, L. Tarraga, N. Tesi, A. Thalamuthu, T. Thomas, G. Tosto, L. Traykov, L. Tremolizzo, A. Tybjaerg-Hansen, A. Uitterlinden, A. Ullgren, I. Ulstein, S. Valero, O. Valladares, C. V. Broeckhoven, J. Vance, B. N. Vardarajan, A. van der Lugt, J. V. Dongen, J. van Rooij, J. van Swieten, R. Vandenberghe, F. Verhey, J. S. Vidal, J. Vogelgsang, M. Vyhnalek, M. Wagner, D. Wallon, L. S. Wang, R. Wang, L. Weinhold, J. Wiltfang, G. Windle, B. Woods, M. Yannakoulia, H. Zare, Y. Zhao, X. Zhang, C. Zhu, M. Zulaica; EADB; GR@ACE; DEGESCO; EADI; GERAD; Demgene; FinnGen; ADGC; CHARGE, L. A. Farrer, B. M. Psaty, M. Ghanbari, T. Raj, P. Sachdev, K. Mather, F. Jessen, M. A. Ikram, A. de Mendonca,

J. Hort, M. Tsolaki, M. A. Pericak-Vance, P. Amouyel, J. Williams, R. Frikke-Schmidt, J. Clarimon, J. F. Deleuze, G. Rossi, S. Seshadri, O. A. Andreassen, M. Ingelsson, M. Hiltunen, K. Sleegers, G. D. Schellenberg, C. M. van Duijn, R. Sims, W. M. van der Flier, A. Ruiz, A. Ramirez, J. C. Lambert, New insights into the genetic etiology of Alzheimer's disease and related dementias. *Nat. Genet.* **54**, 412–436 (2022).

123. J. Ortuno-Sierra, E. Fonseca-Pedrero, M. Paino, S. S. i Riba, J. Muniz, Screening mental health problems during adolescence: Psychometric properties of the Spanish version of the strengths and difficulties questionnaire. *J. Adolesc.* **38**, 49–56 (2015).

124. K. Ni, Z. Huang, Y. Zhu, D. Xue, Q. Jin, C. Zhang, C. Gu, The lncRNA ADAMTS9-AS2 regulates RPL22 to modulate TNBC progression via controlling the TGF-β signaling pathway. *Front. Oncol.* **11**, 654472 (2021).

125. S. Martin, M. Cule, N. Basty, J. Tyrrell, R. N. Beaumont, A. R. Wood, T. M. Frayling, E. Sorokin, B. Whitcher, Y. Liu, J. D. Bell, E. L. Thomas, H. Yaghootkar, Genetic evidence for different adiposity phenotypes and their opposing influences on ectopic fat and risk of cardiometabolic disease. *Diabetes* **70**, 1843–1856 (2021).

126. D. van der Meer, A. A. Shadrin, K. O'Connell, F. Bettella, S. Djurovic, T. Wolfers, D. Alnaes, I. Agartz, O. B. Smeland, I. Melle, J. M. Sanchez, D. E. J. Linden, A. M. Dale, L. T. Westlye, O. A. Andreassen, O. Frei, T. Kaufmann, Boosting schizophrenia genetics by utilizing genetic overlap with brain morphology. *Biol. Psychiatry* **92**, 291–298 (2022).

127. G. Kichaev, G. Bhatia, P. R. Loh, S. Gazal, K. Burch, M. K. Freund, A. Schoech, B. Pasaniuc, A. L. Price, Leveraging polygenic functional enrichment to improve GWAS power. *Am. J. Hum. Genet.* **104**, 65–75 (2019).

128. G. Hindley, A. A. Shadrin, D. van der Meer, N. Parker, W. Cheng, K. S. O'Connell, S. Bahrami, A. Lin, N. Karadag, B. Holen, T. Bjella, I. J. Deary, G. Davies, W. D. Hill, J. Bressler, S. Seshadri, C. C. Fan, T. Ueland, S. Djurovic, O. B. Smeland, O. Frei, A. M. Dale, O. A. Andreassen, Multivariate genetic analysis of personality and cognitive traits reveals abundant pleiotropy. *Nat. Hum. Behav.* **7**, 1584–1600 (2023).

129. A. R. Smith, R. G. Smith, J. Burrage, C. Troakes, S. Al-Sarraj, R. N. Kalaria, C. Sloan, A. C. Robinson, J. Mill, K. Lunnon, A cross-brain regions study of ANK1 DNA methylation in different neurodegenerative diseases. *Neurobiol. Aging* **74**, 70–76 (2019).

130. M. Vujkovic, J. M. Keaton, J. A. Lynch, D. R. Miller, J. Zhou, C. Tcheandjieu, J. E. Huffman, T. L. Assimes, K. Lorenz, X. Zhu, A. T. Hilliard, R. L. Judy, J. Huang, K. M. Lee, D. Klarin, S. Pyarajan, J. Danesh, O. Melander, A. Rasheed, N. H. Mallick, S. Hameed, I. H. Qureshi, M. N. Afzal, U. Malik, A. Jalal, S. Abbas, X. Sheng, L. Gao, K. H. Kaestner, K. Susztak, Y. V. Sun, S. L. DuVall, K. Cho, J. S. Lee, J. M. Gaziano, L. S. Phillips, J. B. Meigs, P. D. Reaven, P. W. Wilson, T. L. Edwards, D. J. Rader, S. M. Damrauer, C. J. O'Donnell, P. S. Tsao; The HPAP Consortium; Regeneron Genetics Center; VA Million Veteran Program, K. M. Chang, B. F. Voight, D. Saleheen, Discovery of 318 new risk loci for type 2 diabetes and related vascular outcomes among 1.4 million participants in a multi-ancestry meta-analysis. *Nat. Genet.* **52**, 680–691 (2020).

131. M. H. Chen, L. M. Raffield, A. Mousas, S. Sakaue, J. E. Huffman, A. Moscati, B. Trivedi, T. Jiang, P. Akbari, D. Vuckovic, E. L. Bao, X. Zhong, R. Manansala, V. Laplante, M. Chen, K. S. Lo, H. Qian, C. A. Lareau, M. Beaudoin, K. A. Hunt, M. Akiyama, T. M. Bartz, Y. Ben-Shlomo, A. Beswick, J. Bork-Jensen, E. P. Bottinger, J. A. Brody, F. J. A. van Rooij, K. Chitrala, K. Cho, H. Choquet, A. Correa, J. Danesh, E. Di Angelantonio, N. Dimou, J. Ding, P. Elliott, T. Esko, M. K. Evans, J. S. Floyd, L. Broer, N. Grarup, M. H. Guo, A. Greinacher, J. Haessler, T. Hansen, J. M. M. Howson, Q. Q. Huang, W. Huang, E. Jorgenson, T. Kacprowski, M. Kahonen, Y. Kamatani, M. Kanai, S. Karthikeyan, F. Koskeridis, L. A. Lange, T. Lehtimaki, M. M. Lerch, A. Linneberg, Y. Liu, L. P. Lyytikainen, A. Manichaikul, H. C. Martin, K. Matsuda, K. L. Mohlke, N. Mononen, Y. Murakami, G. N. Nadkarni, M. Nauck, K. Nikus, W. H. Ouwehand, N. Pankratz, O. Pedersen, M. Preuss, B. M. Psaty, O. T. Raitakari, D. J. Roberts, S. S. Rich, B. A. T. Rodriguez, J. D. Rosen, J. I. Rotter, P. Schubert, C. N. Spracklen, P. Surendran, H. Tang, J. C. Tardif, R. C. Trembath, M. Ghanbari, U. Volker, H. Volzke, N. A. Watkins, A. B. Zonderman, V. A. M. V. Program, P. W. F. Wilson, Y. Li, A. S. Butterworth, J. F. Gauchat, C. W. K. Chiang, B. Li, R. J. F. Loos, W. J. Astle, E. Evangelou, D. A. van Heel, V. G. Sankaran, Y. Okada, N. Soranzo, A. D. Johnson, A. P. Reiner, P. L. Auer, G. Lettre, Trans-ethnic and ancestry-specific blood-cell genetics in 746,667 individuals from 5 global populations. *Cell* **182**, 1198–1213.e14 (2020).

132. S. Christakoudi, E. Evangelou, E. Riboli, K. K. Tsilidis, GWAS of allometric body-shape indices in UK Biobank identifies loci suggesting associations with morphogenesis, organogenesis, adrenal cell renewal and cancer. *Sci. Rep.* **11**, 10688 (2021).

133. F. Koskeridis, E. Evangelou, S. Said, J. J. Boyle, P. Elliott, A. Dehghan, I. Tzoulaki, Pleiotropic genetic architecture and novel loci for C-reactive protein levels. *Nat. Commun.* **13**, 6939 (2022).

134. M. Luciano, S. P. Hagenaars, G. Davies, W. D. Hill, T. K. Clarke, M. Shirali, S. E. Harris, R. E. Marioni, D. C. Liewald, C. Fawns-Ritchie, M. J. Adams, D. M. Howard, C. M. Lewis, C. R. Gale, A. M. McIntosh, I. J. Deary, Association analysis in over 329,000 individuals identifies 116 independent variants influencing neuroticism. *Nat. Genet.* **50**, 6–11 (2018).

135. T. Okabe, T. Nakamura, Y. N. Nishimura, K. Kohu, S. Ohwada, Y. Morishita, T. Akiyama, RICS, a novel GTPase-activating protein for Cdc42 and Rac1, is involved in the beta-catenin-N-cadherin and N-methyl-D-aspartate receptor signaling. *J. Biol. Chem.* **278**, 9920–9927 (2003).

136. L. Yengo, S. Vedantam, E. Marouli, J. Sidorenko, E. Bartell, S. Sakaue, M. Graff, A. U. Eliasen, Y. Jiang, S. Raghavan, J. Miao, J. D. Arias, S. E. Graham, R. E. Mukamel, C. N. Spracklen, X. Yin, S. H. Chen, T. Ferreira, H. H. Highland, Y. Ji, T. Karaderi, K. Lin, K. Lull, D. E. Malden, C. Medina-Gomez, M. Machado, A. Moore, S. Rueger, X. Sim, S. Vrieze, T. S. Ahluwalia, M. Akiyama, M. A. Allison, M. Alvarez, M. K. Andersen, A. Ani, V. Appadurai, L. Arbeeva, S. Bhaskar, L. F. Bielak, S. Bollepalli, L. L. Bonnycastle, J. Bork-Jensen, J. P. Bradfield, Y. Bradford, P. S. Braund, J. A. Brody, K. S. Burgdorf, B. E. Cade, H. Cai, Q. Cai, A. Campbell, M. Canadas-Garre, E. Catamo, J. F. Chai, X. Chai, L. C. Chang, Y. C. Chang, C. H. Chen, A. Chesi, S. H. Choi, R. H. Chung, M. Cocca, M. P. Concas, C. Couture, G. Cuellar-Partida, R. Danning, E. W. Daw, F. Degenhard, G. E. Delgado, A. Delitala, A. Demirkan, X. Deng, P. Devineni, A. Dietl, M. Dimitriou, L. Dimitrov, R. Dorajoo, A. B. Ekici, J. E. Engmann, Z. Fairhurst-Hunter, A. E. Farmaki, J. D. Faul, J. C. Fernandez-Lopez, L. Forer, M. Francescatto, S. Freitag-Wolf, C. Fuchsberger, T. E. Galesloot, Y. Gao, Z. Gao, F. Geller, O. Giannakopoulou, F. Giulianini, A. P. Gjesing, A. Goel, S. D. Gordon, M. Gorski, J. Grove, X. Guo, S. Gustafsson, J. Haessler, T. F. Hansen,

A. S. Havulinna, S. J. Haworth, J. He, N. Heard-Costa, P. Hebbar, G. Hindy, Y. A. Ho, E. Hofer, E. Holliday, K. Horn, W. E. Hornsby, J. J. Hottenga, H. Huang, J. Huang, A. Huerta-Chagoya, J. E. Huffman, Y. J. Hung, S. Huo, M. Y. Hwang, H. Iha, D. D. Ikeda, M. Isono, A. U. Jackson, S. Jager, I. E. Jansen, I. Johansson, J. B. Jonas, A. Jonsson, T. Jorgensen, I. P. Kalafati, M. Kanai, S. Kanoni, L. L. Karhus, A. Kasturiratne, T. Katsuya, T. Kawaguchi, R. L. Kember, K. A. Kentistou, H. N. Kim, Y. J. Kim, M. E. Kleber, M. J. Knol, A. Kurbasic, M. Lauzon, P. Le, R. Lea, J. Y. Lee, H. L. Leonard, S. A. Li, X. Li, X. Li, J. Liang, H. Lin, S. Y. Lin, J. Liu, X. Liu, K. S. Lo, J. Long, L. Lores-Motta, J. Luan, V. Lyssenko, L. P. Lyytikainen, A. Mahajan, V. Mamakou, M. Mangino, A. Manichaikul, J. Marten, M. Mattheisen, L. Mavarani, A. F. McDaid, K. Meidtner, T. L. Melendez, J. M. Mercader, Y. Milaneschi, J. E. Miller, I. Y. Millwood, P. P. Mishra, R. E. Mitchell, L. T. Mollehave, A. Morgan, S. Mucha, M. Munz, M. Nakatochi, C. P. Nelson, M. Nethander, C. W. Nho, A. A. Nielsen, I. M. Nolte, S. S. Nongmaithem, R. Noordam, I. Ntalla, T. Nutile, A. Pandit, P. Christofidou, K. Parna, M. Pauper, E. R. B. Petersen, L. V. Petersen, N. Pitkanen, O. Polasek, A. Poveda, M. H. Preuss, S. Pyarajan, L. M. Raffield, H. Rakugi, J. Ramirez, A. Rasheed, D. Raven, N. W. Rayner, C. Riveros, R. Rohde, D. Ruggiero, S. E. Ruotsalainen, K. A. Ryan, M. Sabater-Lleal, R. Saxena, M. Scholz, A. Sendamarai, B. Shen, J. Shi, J. H. Shin, C. Sidore, C. M. Sitlani, R. C. Slieker, R. A. J. Smit, A. V. Smith, J. A. Smith, L. J. Smyth, L. Southam, V. Steinthorsdottir, L. Sun, F. Takeuchi, D. S. P. Tallapragada, K. D. Taylor, B. O. Tayo, C. Tcheandjieu, N. Terzikhan, P. Tesolin, A. Teumer, E. Theusch, D. J. Thompson, G. Thorleifsson, P. Timmers, S. Trompet, C. Turman, S. Vaccargiu, S. W. van der Laan, P. J. van der Most, J. B. van Klinken, J. van Setten, S. S. Verma, N. Verweij, Y. Veturi, C. A. Wang, C. Wang, L. Wang, Z. Wang, H. R. Warren, W. B. Wei, A. R. Wickremasinghe, M. Wielscher, K. L. Wiggins, B. S. Winsvold, A. Wong, Y. Wu, M. Wuttke, R. Xia, T. Xie, K. Yamamoto, J. Yang, J. Yao, H. Young, N. A. Yousri, L. Yu, L. Zeng, W. Zhang, X. Zhang, J. H. Zhao, W. Zhao, W. Zhou, M. E. Zimmermann, M. Zoledziewska, L. S. Adair, H. H. H. Adams, C. A. Aguilar-Salinas, F. Al-Mulla, D. K. Arnett, F. W. Asselbergs, B. O. Asvold, J. Attia, B. Banas, S. Bandinelli, D. A. Bennett, T. Bergler, D. Bharadwaj, G. Biino, H. Bisgaard, E. Boerwinkle, C. A. Boger, K. Bonnelykke, D. I. Boomsma, A. D. Borglum, J. B. Borja, C. Bouchard, D. W. Bowden, I. Brandslund, B. Brumpton, J. E. Buring, M. J. Caulfield, J. C. Chambers, G. R. Chandak, S. J. Chanock, N. Chaturvedi, Y. I. Chen, Z. Chen, C. Y. Cheng, I. E. Christophersen, M. Ciullo, J. W. Cole, F.

S. Collins, R. S. Cooper, M. Cruz, F. Cucca, L. A. Cupples, M. J. Cutler, S. M. Damrauer, T. M. Dantoft, G. J. de Borst, L. de Groot, P. L. De Jager, D. P. V. de Kleijn, H. J. de Silva, G. V. Dedoussis, A. I. den Hollander, S. Du, D. F. Easton, P. J. M. Elders, A. H. Eliassen, P. T. Ellinor, S. Elmstahl, J. Erdmann, M. K. Evans, D. Fatkin, B. Feenstra, M. F. Feitosa, L. Ferrucci, I. Ford, M. Fornage, A. Franke, P. W. Franks, B. I. Freedman, P. Gasparini, C. Gieger, G. Girotto, M. E. Goddard, Y. M. Golightly, C. Gonzalez-Villalpando, P. Gordon-Larsen, H. Grallert, S. F. A. Grant, N. Grarup, L. Griffiths, V. Gudnason, C. Haiman, H. Hakonarson, T. Hansen, C. A. Hartman, A. T. Hattersley, C. Hayward, S. R. Heckbert, C. K. Heng, C. Hengstenberg, A. W. Hewitt, H. Hishigaki, C. B. Hoyng, P. L. Huang, W. Huang, S. C. Hunt, K. Hveem, E. Hypponen, W. G. Iacono, S. Ichihara, M. A. Ikram, C. R. Isasi, R. D. Jackson, M. R. Jarvelin, Z. B. Jin, K. H. Jockel, P. K. Joshi, P. Jousilahti, J. W. Jukema, M. Kahonen, Y. Kamatani, K. D. Kang, J. Kaprio, S. L. R. Kardia, F. Karpe, N. Kato, F. Kee, T. Kessler, A. V. Khera, C. C. Khor, L. Kiemeney, B. J. Kim, E. K. Kim, H. L. Kim, P. Kirchhof, M. Kivimaki, W. P. Koh, H. A. Koistinen, G. D. Kolovou, J. S. Kooner, C. Kooperberg, A. Kottgen, P. Kovacs, A. Kraaijeveld, P. Kraft, R. M. Krauss, M. Kumari, Z. Kutalik, M. Laakso, L. A. Lange, C. Langenberg, L. J. Launer, L. Le Marchand, H. Lee, N. R. Lee, T. Lehtimaki, H. Li, L. Li, W. Lieb, X. Lin, L. Lind, A. Linneberg, C. T. Liu, J. Liu, M. Loeffler, B. London, S. A. Lubitz, S. J. Lye, D. A. Mackey, R. Magi, P. K. E. Magnusson, G. M. Marcus, P. M. Vidal, N. G. Martin, W. Marz, F. Matsuda, R. W. McGarrah, M. McGue, A. J. McKnight, S. E. Medland, D. Mellstrom, A. Metspalu, B. D. Mitchell, P. Mitchell, D. O. Mook-Kanamori, A. D. Morris, L. A. Mucci, P. B. Munroe, M. A. Nalls, S. Nazarian, A. E. Nelson, M. J. Neville, C. Newton-Cheh, C. S. Nielsen, M. M. Nothen, C. Ohlsson, A. J. Oldehinkel, L. Orozco, K. Pahkala, P. Pajukanta, C. N. A. Palmer, E. J. Parra, C. Pattaro, O. Pedersen, C. E. Pennell, B. Penninx, L. Perusse, A. Peters, P. A. Peyser, D. J. Porteous, D. Posthuma, C. Power, P. P. Pramstaller, M. A. Province, Q. Qi, J. Qu, D. J. Rader, O. T. Raitakari, S. Ralhan, L. S. Rallidis, D. C. Rao, S. Redline, D. F. Reilly, A. P. Reiner, S. Y. Rhee, P. M. Ridker, M. Rienstra, S. Ripatti, M. D. Ritchie, D. M. Roden, F. R. Rosendaal, J. I. Rotter, I. Rudan, F. Rutters, C. Sabanayagam, D. Saleheen, V. Salomaa, N. J. Samani, D. K. Sanghera, N. Sattar, B. Schmidt, H. Schmidt, R. Schmidt, M. B. Schulze, H. Schunkert, L. J. Scott, R. J. Scott, P. Sever, E. J. Shiroma, M. B. Shoemaker, X. O. Shu, E. M. Simonsick, M. Sims, J. R. Singh, A. B. Singleton, M. F. Sinner, J. G. Smith, H. Snieder, T. D. Spector, M. J. Stampfer, K. J. Stark, D. P. Strachan, L. M. Hart, Y. Tabara, H.

Tang, J. C. Tardif, T. A. Thanaraj, N. J. Timpson, A. Tonjes, A. Tremblay, T. Tuomi, J. Tuomilehto, M. T. Tusie-Luna, A. G. Uitterlinden, R. M. van Dam, P. van der Harst, N. Van der Velde, C. M. van Duijn, N. M. van Schoor, V. Vitart, U. Volker, P. Vollenweider, H. Volzke, N. H. Wacher-Rodarte, M. Walker, Y. X. Wang, N. J. Wareham, R. M. Watanabe, H. Watkins, D. R. Weir, T. M. Werge, E. Widen, L. R. Wilkens, G. Willemsen, W. C. Willett, J. F. Wilson, T. Y. Wong, J. T. Woo, A. F. Wright, J. Y. Wu, H. Xu, C. S. Yajnik, M. Yokota, J. M. Yuan, E. Zeggini, B. S. Zemel, W. Zheng, X. Zhu, J. M. Zmuda, A. B. Zonderman, J. A. Zwart; 23andMe Research Team; VA Million Veteran Program; DiscovEHR (DiscovEHR and MyCode Community Health Initiative); eMERGE (Electronic Medical Records and Genomics Network); Lifelines Cohort Study; The PRACTICAL Consortium; Understanding Society Scientific Group, D. I. Chasman, Y. S. Cho, I. M. Heid, M. I. McCarthy, M. C. Y. Ng, C. J. O'Donnell, F. Rivadeneira, U. Thorsteinsdottir, Y. V. Sun, E. S. Tai, M. Boehnke, P. Deloukas, A. E. Justice, C. M. Lindgren, R. J. F. Loos, K. L. Mohlke, K. E. North, K. Stefansson, R. G. Walters, T. W. Winkler, K. L. Young, P. R. Loh, J. Yang, T. Esko, T. L. Assimes, A. Auton, G. R. Abecasis, C. J. Willer, A. E. Locke, S. I. Berndt, G. Lettre, T. M. Frayling, Y. Okada, A. R. Wood, P. M. Visscher, J. N. Hirschhorn, A saturated map of common genetic variants associated with human height. *Nature* **610**, 704–712 (2022).

137. G. R. Uhl, M. J. Martinez, PTPRD: Neurobiology, genetics, and initial pharmacology of a pleiotropic contributor to brain phenotypes. *Ann. N. Y. Acad. Sci.* **1451**, 112–129 (2019).

138. S. Rajakulendran, D. Kaski, M. G. Hanna, Neuronal P/Q-type calcium channel dysfunction in inherited disorders of the CNS. *Nat. Rev. Neurol.* **8**, 86–96 (2012).

139. S. Naqvi, Y. Sleyp, H. Hoskens, K. Indencleef, J. P. Spence, R. Bruffaerts, A. Radwan, R. J. Eller, S. Richmond, M. D. Shriver, J. R. Shaffer, S. M. Weinberg, S. Walsh, J. Thompson, J. K. Pritchard, S. Sunaert, H. Peeters, J. Wysocka, P. Claes, Shared heritability of human face and brain shape. *Nat. Genet.* **53**, 830–839 (2021).

140. B. Zhao, T. Luo, T. Li, Y. Li, J. Zhang, Y. Shan, X. Wang, L. Yang, F. Zhou, Z. Zhu; Alzheimer's Disease Neuroimaging Initiative; Pediatric Imaging, Neurocognition and Genetics, H. Zhu, Genome-wide association analysis of 19,629 individuals identifies

variants influencing regional brain volumes and refines their genetic co- architecture with cognitive and mental health traits. *Nat. Genet.* **51**, 1637–1644 (2019).

141. G. Lauc, J. E. Huffman, M. Pucic, L. Zgaga, B. Adamczyk, A. Muzinic, M. Novokmet, O. Polasek, O. Gornik, J. Kristic, T. Keser, V. Vitart, B. Scheijen, H. W. Uh, M. Molokhia, A. L. Patrick, P. McKeigue, I. Kolcic, I. K. Lukic, O. Swann, F. N. van Leeuwen, L. R. Ruhaak, J. J. Houwing-Duistermaat, P. E. Slagboom, M. Beekman, A. J. de Craen, A. M. Deelder, Q. Zeng, W. Wang, N. D. Hastie, U. Gyllensten, J. F. Wilson, M. Wuhrer, A. F. Wright, P. M. Rudd, C. Hayward, Y. Aulchenko, H. Campbell, I. Rudan, Loci associated with N-glycosylation of human immunoglobulin G show pleiotropy with autoimmune diseases and haematological cancers. *PLOS Genet.* **9**, e1003225 (2013).

142. J. Huang, J. E. Huffman, Y. Huang, I. Do Valle, T. L. Assimes, S. Raghavan, B. F. Voight, C. Liu, A. L. Barabasi, R. D. L. Huang, Q. Hui, X. T. Nguyen, Y. L. Ho, L. Djousse, J. A. Lynch, M. Vujkovic, C. Tcheandjieu, H. Tang, S. M. Damrauer, P. D. Reaven, D. Miller, L. S. Phillips, M. C. Y. Ng, M. Graff, C. A. Haiman, R. J. F. Loos, K. E. North, L. Yengo, G. D. Smith, D. Saleheen, J. M. Gaziano, D. J. Rader, P. S. Tsao, K. Cho, K. M. Chang, P. W. F. Wilson, V. A. M. V. Program, Y. V. Sun, C. J. O'Donnell, Genomics and phenomics of body mass index reveals a complex disease network. *Nat. Commun.* **13**, 7973 (2022).

143. K. L. Grasby, N. Jahanshad, J. N. Painter, L. Colodro-Conde, J. Bralten, D. P. Hibar, P. A. Lind, F. Pizzagalli, C. R. K. Ching, M. A. B. McMahon, N. Shatokhina, L. C. P. Zsembik, S. I. Thomopoulos, A. H. Zhu, L. T. Strike, I. Agartz, S. Alhusaini, M. A. A. Almeida, D. Alnaes, I. K. Amlien, M. Andersson, T. Ard, N. J. Armstrong, A. Ashley-Koch, J. R. Atkins, M. Bernard, R. M. Brouwer, E. E. L. Buimer, R. Bulow, C. Burger, D. M. Cannon, M. Chakravarty, Q. Chen, J. W. Cheung, B. Couvy-Duchesne, A. M. Dale, S. Dalvie, T. K. de Araujo, G. I. de Zubicaray, S. M. C. de Zwarte, A. den Braber, N. T. Doan, K. Dohm, S. Ehrlich, H. R. Engelbrecht, S. Erk, C. C. Fan, I. O. Fedko, S. F. Foley, J. M. Ford, M. Fukunaga, M. E. Garrett, T. Ge, S. Giddaluru, A. L. Goldman, M. J. Green, N. A. Groenewold, D. Grotegerd, T. P. Gurholt, B. A. Gutman, N. K. Hansell, M. A. Harris, M. B. Harrison, C. C. Haswell, M. Hauser, S. Herms, D. J. Heslenfeld, N. F. Ho, D. Hoehn, P. Hoffmann, L. Holleran, M. Hoogman, J. J. Hottenga, M. Ikeda, D. Janowitz, I. E. Jansen, T. Jia, C. Jockwitz, R. Kanai, S. Karama, D. Kasperaviciute, T. Kaufmann, S. Kelly, M.

Kikuchi, M. Klein, M. Knapp, A. R. Knodt, B. Kramer, M. Lam, T. M. Lancaster, P. H. Lee, T. A. Lett, L. B. Lewis, I. Lopes-Cendes, M. Luciano, F. Macciardi, A. F. Marquand, S. R. Mathias, T. R. Melzer, Y. Milaneschi, N. Mirza-Schreiber, J. C. V. Moreira, T. W. Muhleisen, B. Muller-Myhsok, P. Najt, S. Nakahara, K. Nho, L. M. O. Loohuis, D. P. Orfanos, J. F. Pearson, T. L. Pitcher, B. Putz, Y. Quide, A. Ragothaman, F. M. Rashid, W. R. Reay, R. Redlich, C. S. Reinbold, J. Repple, G. Richard, B. C. Riedel, S. L. Risacher, C. S. Rocha, N. R. Mota, L. Salminen, A. Saremi, A. J. Saykin, F. Schlag, L. Schmaal, P. R. Schofield, R. Secolin, C. Y. Shapland, L. Shen, J. Shin, E. Shumskaya, I. E. Sonderby, E. Sprooten, K. E. Tansey, A. Teumer, A. Thalamuthu, D. Tordesillas-Gutierrez, J. A. Turner, A. Uhlmann, C. L. Vallerga, D. van der Meer, M. M. J. van Donkelaar, L. van Eijk, T. G. M. van Erp, N. E. M. van Haren, D. van Rooij, M. J. van Tol, J. H. Veldink, E. Verhoef, E. Walton, M. Wang, Y. Wang, J. M. Wardlaw, W. Wen, L. T. Westlye, C. D. Whelan, S. H. Witt, K. Wittfeld, C. Wolf, T. Wolfers, J. Q. Wu, C. L. Yasuda, D. Zaremba, Z. Zhang, M. P. Zwiers, E. Artiges, A. A. Assareh, R. Ayesa-Arriola, A. Belger, C. L. Brandt, G. G. Brown, S. Cichon, J. E. Curran, G. E. Davies, F. Degenhardt, M. F. Dennis, B. Dietsche, S. Djurovic, C. P. Doherty, R. Espiritu, D. Garijo, Y. Gil, P. A. Gowland, R. C. Green, A. N. Hausler, W. Heindel, B. C. Ho, W. U. Hoffmann, F. Holsboer, G. Homuth, N. Hosten, C. R. Jack Jr., M. Jang, A. Jansen, N. A. Kimbrel, K. Kolskar, S. Koops, A. Krug, K. O. Lim, J. J. Luykx, D. H. Mathalon, K. A. Mather, V. S. Mattay, S. Matthews, J. M. Van Son, S. C. McEwen, I. Melle, D. W. Morris, B. A. Mueller, M. Nauck, J. E. Nordvik, M. M. Nothen, D. S. O'Leary, N. Opel, M. P. Martinot, G. B. Pike, A. Preda, E. B. Quinlan, P. E. Rasser, V. Ratnakar, S. Reppermund, V. M. Steen, P. A. Tooney, F. R. Torres, D. J. Veltman, J. T. Voyvodic, R. Whelan, T. White, H. Yamamori, H. H. H. Adams, J. C. Bis, S. Debette, C. Decarli, M. Fornage, V. Gudnason, E. Hofer, M. A. Ikram, L. Launer, W. T. Longstreth, O. L. Lopez, B. Mazoyer, T. H. Mosley, G. V. Roshchupkin, C. L. Satizabal, R. Schmidt, S. Seshadri, Q. Yang; Alzheimer's Disease Neuroimaging Initiative; CHARGE Consortium; EPIGEN Consortium; IMAGEN Consortium; SYS Consortium; Parkinson's Progression Markers Initiative, M. K. M. Alvim, D. Ames, T. J. Anderson, O. A. Andreassen, A. Arias-Vasquez, M. E. Bastin, B. T. Baune, J. C. Beckham, J. Blangero, D. I. Boomsma, H. Brodaty, H. G. Brunner, R. L. Buckner, J. K. Buitelaar, J. R. Bustillo, W. Cahn, M. J. Cairns, V. Calhoun, V. J. Carr, X. Caseras, S. Caspers, G. L. Cavalleri, F. Cendes, A. Corvin, B. Crespo-Facorro, J. C. Dalrymple-Alford, U. Dannlowski, E. J. C. de Geus, I. J. Deary, N. Delanty, C. Depondt,

S. Desrivieres, G. Donohoe, T. Espeseth, G. Fernandez, S. E. Fisher, H. Flor, A. J. Forstner, C. Francks, B. Franke, D. C. Glahn, R. L. Gollub, H. J. Grabe, O. Gruber, A. K. Haberg, A. R. Hariri, C. A. Hartman, R. Hashimoto, A. Heinz, F. A. Henskens, M. H. J. Hillegers, P. J. Hoekstra, A. J. Holmes, L. E. Hong, W. D. Hopkins, H. E. H. Pol, T. L. Jernigan, E. G. Jonsson, R. S. Kahn, M. A. Kennedy, T. T. J. Kircher, P. Kochunov, J. B. J. Kwok, S. Le Hellard, C. M. Loughland, N. G. Martin, J. L. Martinot, C. McDonald, K. L. McMahon, A. Meyer-Lindenberg, P. T. Michie, R. A. Morey, B. Mowry, L. Nyberg, J. Oosterlaan, R. A. Ophoff, C. Pantelis, T. Paus, Z. Pausova, B. Penninx, T. J. C. Polderman, D. Posthuma, M. Rietschel, J. L. Roffman, L. M. Rowland, P. S. Sachdev, P. G. Samann, U. Schall, G. Schumann, R. J. Scott, K. Sim, S. M. Sisodiya, J. W. Smoller, I. E. Sommer, B. S. Pourcain, D. J. Stein, A. W. Toga, J. N. Trollor, N. J. A. Van der Wee, D. van't Ent, H. Volzke, H. Walter, B. Weber, D. R. Weinberger, M. J. Wright, J. Zhou, J. L. Stein, P. M. Thompson, S. E. Medland; Enhancing NeuroImaging Genetics through Meta-Analysis Consortium (ENIGMA)—Genetics working group, The genetic architecture of the human cerebral cortex. *Science* **367**, eaay6690 (2020).

144. A. A. Shadrin, T. Kaufmann, D. van der Meer, C. E. Palmer, C. Makowski, R. Loughnan, T. L. Jernigan, T. M. Seibert, D. J. Hagler, O. B. Smeland, E. Motazedi, Y. Chu, A. Lin, W. Cheng, G. Hindley, W. K. Thompson, C. C. Fan, D. Holland, L. T. Westlye, O. Frei, O. A. Andreassen, A. M. Dale, Vertex-wise multivariate genome-wide association study identifies 780 unique genetic loci associated with cortical morphology. *Neuroimage* **244**, 118603 (2021).

145. T. Alam, M. R. An, J. Papaconstantinou, Differential expression of three C/EBP isoforms in multiple tissues during the acute phase response. *J. Biol. Chem.* **267**, 5021–5024 (1992).

146. K. M. de Lange, L. Moutsianas, J. C. Lee, C. A. Lamb, Y. Luo, N. A. Kennedy, L. Jostins, D. L. Rice, J. Gutierrez-Achury, S. G. Ji, G. Heap, E. R. Nimmo, C. Edwards, P. Henderson, C. Mowat, J. Sanderson, J. Satsangi, A. Simmons, D. C. Wilson, M. Tremelling, A. Hart, C. G. Mathew, W. G. Newman, M. Parkes, C. W. Lees, H. Uhlig, C. Hawkey, N. J. Prescott, T. Ahmad, J. C. Mansfield, C. A. Anderson, J. C. Barrett, Genome-wide association study implicates immune activation of multiple integrin genes in inflammatory bowel disease. *Nat. Genet.* **49**, 256–261 (2017).

147. B. M. Javierre, O. S. Burren, S. P. Wilder, R. Kreuzhuber, S. M. Hill, S. Sewitz, J. Cairns, S. W. Wingett, C. Varnai, M. J. Thiecke, F. Burden, S. Farrow, A. J. Cutler, K. Rehnstrom, K. Downes, L. Grassi, M. Kostadima, P. Freire-Pritchett, F. Wang; BLUEPRINT Consortium, H. G. Stunnenberg, J. A. Todd, D. R. Zerbino, O. Stegle, W. H. Ouwehand, M. Frontini, C. Wallace, M. Spivakov, P. Fraser, Lineage-specific genome architecture links enhancers and non-coding disease variants to target gene promoters. *Cell* **167**, 1369–1384.e19 (2016).

148. K. P. Lesch, N. Timmesfeld, T. J. Renner, R. Halperin, C. Roser, T. T. Nguyen, D. W. Craig, J. Romanos, M. Heine, J. Meyer, C. Freitag, A. Warnke, M. Romanos, H. Schafer, S. Walitza, A. Reif, D. A. Stephan, C. Jacob, Molecular genetics of adult ADHD: Converging evidence from genome-wide association and extended pedigree linkage studies. *J. Neural Transm.* **115**, 1573–1585 (2008).

149. T. T. Chu, Y. Liu, An integrated genomic analysis of gene-function correlation on schizophrenia susceptibility genes. *J. Hum. Genet.* **55**, 285–292 (2010).

150. L. J. Scott, P. Muglia, X. Q. Kong, W. Guan, M. Flickinger, R. Upmanyu, F. Tozzi, J. Z. Li, M. Burmeister, D. Absher, R. C. Thompson, C. Francks, F. Meng, A. Antoniades, A. M. Southwick, A. F. Schatzberg, W. E. Bunney, J. D. Barchas, E. G. Jones, R. Day, K. Matthews, P. McGuffin, J. S. Strauss, J. L. Kennedy, L. Middleton, A. D. Roses, S. J. Watson, J. B. Vincent, R. M. Myers, A. E. Farmer, H. Akil, D. K. Burns, M. Boehnke, Genome-wide association and meta-analysis of bipolar disorder in individuals of European ancestry. *Proc. Natl. Acad. Sci. U.S.A.* **106**, 7501–7506 (2009).

151. The Schizophrenia Psychiatric Genome-Wide Association Study (GWAS) Consortium, Genome-wide association study identifies five new schizophrenia loci. *Nat. Genet.* **43**, 969–976 (2011).

152. T. W. Muhleisen, M. Leber, T. G. Schulze, J. Strohmaier, F. Degenhardt, J. Treutlein, M. Mattheisen, A. J. Forstner, J. Schumacher, R. Breuer, S. Meier, S. Herms, P. Hoffmann, A. Lacour, S. H. Witt, A. Reif, B. Muller-Myhsok, S. Lucae, W. Maier, M. Schwarz, H. Vedder, J. Kammerer-Ciernioch, A. Pfennig, M. Bauer, M. Hautzinger, S. Moebus, L. Priebe, P. M. Czerski, J. Hauser, J. Lissowska, N. Szeszenia-Dabrowska, P. Brennan, J. D. McKay, A. Wright, P. B. Mitchell, J. M. Fullerton, P. R. Schofield, G. W. Montgomery, S. E. Medland, S. D. Gordon, N. G. Martin, V. Krasnow, A. Chuchalin, G. Babadjanova, G. Pantelejeva, L. I. Abramova, A. S. Tiganov, A. Polonikov, E. Khusnutdinova, M. Alda, P. Grof, G. A. Rouleau,

G. Turecki, C. Laprise, F. Rivas, F. Mayoral, M. Kogevinas, M. Grigoroiu-Serbanescu, P. Propping, T. Becker, M. Rietschel, M. M. Nothen, S. Cichon, Genome-wide association study reveals two new risk loci for bipolar disorder. *Nat. Commun.* **5**, 3339 (2014).

153. Cross-Disorder Group of the Psychiatric Genomics Consortium, Identification of risk loci with shared effects on five major psychiatric disorders: A genome-wide analysis. *Lancet* **381**, 1371–1379 (2013).

154. S. M. Smith, G. Douaud, W. Chen, T. Hanayik, F. Alfaro-Almagro, K. Sharp, L. T. Elliott, An expanded set of genome-wide association studies of brain imaging phenotypes in UK Biobank. *Nat. Neurosci.* **24**, 737–745 (2021).

155. A. G. Comuzzie, S. A. Cole, S. L. Laston, V. S. Voruganti, K. Haack, R. A. Gibbs, N. F. Butte, Novel genetic loci identified for the pathophysiology of childhood obesity in the Hispanic population. *PLOS ONE* **7**, e51954 (2012).

156. A. Subramanian, P. Tamayo, V. K. Mootha, S. Mukherjee, B. L. Ebert, M. A. Gillette, A. Paulovich, S. L. Pomeroy, T. R. Golub, E. S. Lander, J. P. Mesirov, Gene set enrichment analysis: A knowledge-based approach for interpreting genome-wide expression profiles. *Proc. Natl. Acad. Sci. U.S.A.* **102**, 15545–15550 (2005).
